# Genetic and Transcriptomic Background of Oxidative Stress and Antioxidative Therapies in Late Complications of Type 2 Diabetes Mellitus: A Systematic Review

**DOI:** 10.3390/antiox13030277

**Published:** 2024-02-24

**Authors:** Gašper Tonin, Vita Dolžan, Jasna Klen

**Affiliations:** 1 Faculty of Medicine, University of Ljubljana, 1000 Ljubljana, Slovenia; toningasper@gmail.si; 2 Faculty of Arts, University of Ljubljana, 1000 Ljubljana, Slovenia; 3 Pharmacogenetics Laboratory, Institute of Biochemistry and Molecular Genetics, Faculty of Medicine, University of Ljubljana, 1000 Ljubljana, Slovenia; 4 Division of Surgery, Department of Abdominal Surgery, University Medical Centre Ljubljana, 1000 Ljubljana, Slovenia; 5 Department of Internal Medicine, Faculty of Medicine, University of Ljubljana, 1000 Ljubljana, Slovenia

**Keywords:** type 2 diabetes mellitus, microvascular complications, macrovascular complications, genetic polymorphisms, transcriptomics, antioxidants, oxidative stress

## Abstract

This systematic review extensively investigated the role of the genetic and transcriptomic factors in late complications of type 2 diabetes mellitus (T2DM) and the current approaches targeting oxidative-stress-related pathways with antioxidant therapies. To cover our broad research area, we have conducted two systematic searches, the first focusing on genetic and transcriptomic factors affecting oxidative stress and the second one focusing on the antioxidant therapies in late complications of T2DM. The final review included 33 genetic and transcriptomic studies and 23 interventional randomized clinical trials. The conducted systematic review highlights the important role of oxidative stress in the development of late complications in T2DM patients. However, the current level of evidence does not support the use of genetic and transcriptomic factors as predictive and prognostic biomarkers for the development of T2DM late complications. Further studies are needed to elucidate the potential of targeting oxidative-stress-related pathways for novel preventative and therapeutic approaches. Additionally, antioxidants both in dietary and supplement form have been shown to improve different metabolic and biochemical parameters in T2DM patients with developed late complications. In recent years, studies have improved in methodological quality despite still mainly focusing on microvascular late complications of T2DM. Furthermore, the observed interventional studies suggest non-homogeneity in the duration of observation. As many studies do not provide post-intervention follow-up testing, it is difficult to assess the long-term health benefits of antioxidant supplementation.

## 1. Introduction

It is known that 541 million adults have impaired glucose tolerance, which could lead to type 2 diabetes mellitus (T2DM). Furthermore, it is estimated that 232 million patients are underdiagnosed. T2DM is one of the most common chronic metabolic diseases. Several environmental and genetic factors might lead to impaired insulin production by pancreatic β-cells and impaired insulin sensitivity in tissues, resulting in the clinical manifestation of hyperglycemia [1]. Macrovascular and microvascular complications play a crucial role in the 15% increase in all-cause mortality and morbidity in this group of patients. Moreover, they present extreme physical and psychological distress to patients and caregivers and an enormous burden on the healthcare system [2].

Among microvascular complications, the most often is diabetic retinopathy (DR). The retina is vulnerable to oxidative stress since it is constantly exposed to reactive oxygen species (ROS) producing visible light or UV. Moreover, in the outer photoreceptor segment membranes of the retina, there are plenty of easily oxidizable poly-unsaturated fatty acids (PUFAs), such as docosahexaenoic acid (DHA), arachidonic acid, and oleic acid. Oxidative stress is very important in the pathogenesis of DR, and four irregularities have been reported to be associated with hyperglycemia-induced oxidative damage in the retina: activation of the protein kinase C (PKC) pathway; polyol flow; activation of the hexosamine pathway; and intracellular formation of advanced glycation end products (AGEs). In addition, oxidative stress also leads to mitochondrial defects, cell apoptosis, inflammation, lipid peroxidation, microcirculatory abnormalities, and neurodegeneration. Globally, DR is the major cause of diabetes-related visual impairment or loss in working-age adults and the elderly. The number of patients affected by DR is expected to rise to 191 million by 2030. The basic pathophysiology of DR is described by hyperglycemia, which causes alterations such as the thickening of the retinal capillary basement membrane, increased retinal vascular permeability, tissue ischemia, and the release of numerous vasoactive chemicals, which leads to neovascularization. There are different stages of DR. Although non-proliferative diabetic retinopathy (NPDR) is a subclass without neovascularization, it is characterized by microaneurysms and small dilation of retinal blood vessels. On the other hand, in proliferative diabetic retinopathy (PDR) new blood vessels grow on the surface of the retina, and since they are unstable, blood and extracellular fluid easily leak out, resulting in vitreous hemorrhage and retinal detachment [3].

Around 20–40% of T2DM patients develop diabetic kidney disease (DKD). Furthermore, in those with end-stage kidney disease, 80% of the time, the culprit is a combination of hyperglycemia and hypertension. The combination of several elements, including glycolipid metabolic disorder and hemodynamic alteration, triggers pathways such as polyols and hexosamines pathways to produce large amounts of ROS, resulting in an oxidative–antioxidant imbalance in the kidney and the induction of oxidative stress responses. This, in turn, triggers the activation of downstream cellular signaling pathways, resulting in the induction of inflammatory responses, autophagy, and fibrosis, accelerating the pathological alterations and functional abnormalities that lead to DKD [4]. DKD has been linked to hypoxia and the production of angiotensin II (AngII). All the processes result in the restructuring of the actin cytoskeleton inside podocytes, producing podocyte damage, affecting the integrity of the glomerular filtration barrier, and eventually causing proteinuria. Furthermore, AngII inhibits ROS synthesis via the PKC/NADPH oxidase pathway, and decreasing PKC expression inhibits ROS generation [5].

Approximately 50% of patients with T2DM are affected by diabetic neuropathy, which could lead to an increased number of falls, pain, and a reduction in the quality of life. It is the main cause of foot ulcers and contributes to 50–75% of non-traumatic amputations. Distal symmetric polyneuropathy (DSPN) is the most common presentation of diabetic neuropathy that preferentially targets sensory and autonomic axons and, in later progression, motoric axons. In T2DM, oxidative phosphorylation becomes compromised because of an abundance of the substrate. This leads to a decrease in ATP production and an increase in ROS, which contributes to the failure of mitochondria and the onset of oxidative and metabolic harm to Schwann cells and dorsal root ganglion neurons. Malfunctioning mitochondria provide insufficient energy and lose their ability to move along axons effectively, aggravating axonal disturbances and damage [6].

Roughly 50% of diabetic patients’ deaths can be attributed to cardiovascular disease (CVD), and those with T2DM have a twofold risk of cardiovascular mortality compared to those without the illness. The most common type of CVD is coronary artery disease. Insulin resistance promotes endothelial dysfunction and atherosclerosis by suppressing the PI3K-NO pathway while increasing the activation of the MAPK-ET-1 pathway. Within endothelial cells, insulin resistance stimulates inflammation in vascular tissues, boosts prothrombotic factor levels, increases the presence of ROS, and selectively inhibits insulin-induced nitric oxide generation [7,8].

Oxidative stress occurs when there is an imbalance between the production of ROS species and the cell’s ability to counteract them with antioxidants [9]. Superoxide (O_2_^•−^), hydrogen peroxide (H_2_O_2_), hydroxyl radical (^•^OH), ozone, and singlet oxygen are only a few of the chemicals that fall under the umbrella of ROS [10]. The pathogenesis and progression of various diseases are notably influenced by oxidative stress, which has two main mechanisms. The first process entails the production of reactive species, such as ^•^OH, ONOO-, and HOCL. Membrane lipids, structural proteins, enzymes, and nucleic acids are directly oxidized by ROS. This oxidation process, therefore, causes cellular dysfunction and cell death. The second pathway involves abnormal redox signaling [10]. The accumulation of AGEs and abnormal activation of stress signaling pathways may lead to late diabetic complications [11]. The increased levels of oxidants in T2DM are caused by defective mitochondria and NOX1, which are triggered by hyperglycemia and dyslipidemia, two important pathological states associated with diabetes [12]. A crucial defensive mechanism against ROS is provided by antioxidant enzymes such as superoxide dismutase (SOD), catalase (CAT), and glutathione peroxidase (GPx) [13]. Three variations of SOD multimeric metalloenzymes exist in humans, and they convert the O_2_^•−^ into hydrogen peroxide (H_2_O_2_) and O_2_. Furthermore, H_2_O_2_, which is one of the most stable oxidants, could be converted into water via CAT and GPx [13,14]. SOD1 (Cu/Zn) is located within the cytoplasm and controls the baseline levels of oxidative stress brought on by the generation of superoxide (O_2_^•−^) in the mitochondria and cytoplasm [15]. It could regulate signal transduction pathways involving ROS and starting gene transcription after exposure to neurotoxic stimuli [16]. SOD2 (Mn) resides within the mitochondria, and SOD3 is found extracellularly in the lungs, kidneys, and vessels with the function of O_2_^•−^ dismutation. GPx is a relatively stable substrate-specific enzyme assembled from four identical subunits of 21 kDa containing a selenocysteine residue, needing reduced glutathione, which is an electron donor. CAT is a major tetrameric antioxidant enzyme made up of four tetrahedral subunits containing an active heme group and NADPH [17]. Recently, new findings describing antioxidative defense in T2DM have come to light. Dworzanski et al. found the decreased activity of SOD and GPx in T2DM patients. SOD may be inactivated with hydrogen peroxidase due to the autooxidation of glucose. Additionally, even GPx could be inactivated. Moreover, hyperglycemia and excessive generation of ROS suppress the body’s natural antioxidant defenses and expose cells to oxidative stress damage, which can lead to diabetes late complications [18]. In patients with a long duration of T2DM, the decreased activity of antioxidant enzymes was observed [19].

It is widely acknowledged that genetic variability, as well as environmental factors, including modifiable lifestyle factors such as diet, influence the activity and/or the expression levels of oxidative-stress-related enzymes, thus, contributing to the individual differences observed in antioxidant defense mechanisms and susceptibility to oxidative stress [20]. Therefore, genetic variability and alterations in gene expression levels may also play a role in the development of T2DM late complications [21,22,23].

Taking all of the above into consideration, some of the leading therapeutic prospects in the field of antioxidant research in T2DM are the prevention of oxidant generation that directly damages macromolecules, the inhibition of oxidant-induced downstream signaling that causes inflammation or cell death, and the stimulation of antioxidant enzymes and their substrates [24]. Extensive research on the antioxidant effect of numerous compounds, including natural antioxidants of plant origin, has been carried out. Thus, findings suggesting the significance of different antioxidants in recovering insulin sensitivity were obtained. Many phytochemicals from food and medicinal plants regulate the activities of glucosidase and lipase, lower glycemia, improve pancreatic function, have a synergistic effect with antihyperglycemic drugs, and are, thus, highly effective in the treatment of diabetes [25]. Natural products such as taxifolin, resveratrol, and sulforaphane can protect the diabetic heart [26,27,28,29]. Such effects can also be attributed to N-acetylcysteine (NAC), which may increase plasma cysteine levels and consequently affect the biosynthesis of glutathione [30].

The purpose of our article is to highlight the crucial role of oxidative stress in the development and severity of late complications in patients with T2DM while providing insight into the underlying genetic mechanisms. We have systematically reviewed the published data on various aspects of the role of genetic variability and gene expression levels of oxidative and antioxidative enzymes in the development of late complications of T2DM. We also conducted a comprehensive systematic review of published randomized clinical trials that investigated potential antioxidant therapies in patients with late complications of T2DM.

## 2. Materials and Methods

**Figure 1:**
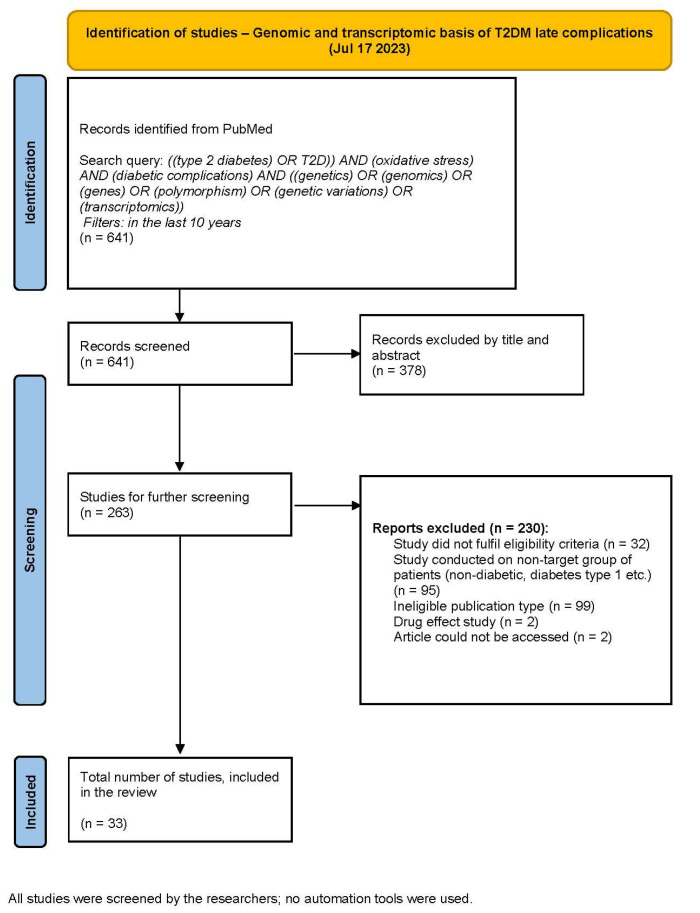
PRISMA flow diagram for the first search.

**Figure 2:**
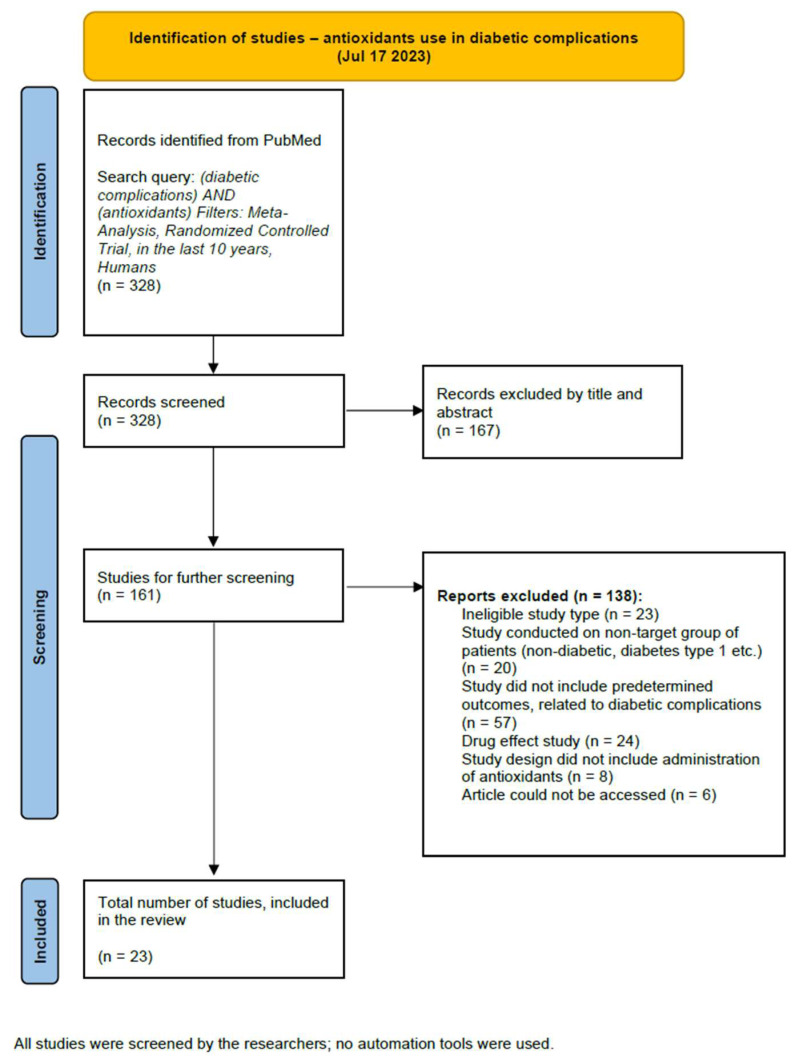
PRISMA flow diagram for the second search.

We conducted the review in accordance with the Preferred Reporting Items for Systematic Review and Meta-Analysis (PRISMA) statement. We have registered our study protocol at INPLASY, International Platform of Registered Systematic Review and Meta-analysis Protocols. Our protocol registration number is INPLASY202420095 (doi: 10.37766/inplasy2024.2.0095). Moreover, we followed the method suggested by Page et al. to visually represent the two systematic searches performed in this review [31]. We have conducted two independent searches as we were not only interested in the genetic background of oxidative stress in the development of T2DM late complications (first search) but also in the various antioxidants that have been administered and tested in clinical trials with T2DM patients (second search). Both protocols are represented in Figure 1 and Figure 2. We have included a more detailed description of the methodology of both of the literature searches in Supplementary File S1.

### 2.1. Eligibility Criteria

In both searches, we focused on the late complications of T2DM. First, we searched for genetic and transcriptomic studies that focused on the role of oxidative stress in the development of microvascular and macrovascular late complications of T2DM (DR, DKD, diabetic neuropathy; cerebrovascular disease, CVD, and PVD). In the second search, we reviewed the interventional randomized clinical trials with antioxidants in patients with T2DM late complications. The eligibility criteria for both of the literature searches are presented more extensively in Supplementary File S1.

### 2.2. Search Strategy

We conducted both the first and second search on 17 July 2023. We decided to include only scientific articles indexed by PubMed as it primarily includes peer-reviewed journals and publications that are more likely to meet quality and reliability standards. Moreover, the field of antioxidants research is prone to unreliable research practices, and the research articles published in PubMed are mostly peer-reviewed, thus, possibly improving the quality of the published research [32,33,34,35]. Nevertheless, we are aware of the publication bias that might arise by choosing only one database. Additionally, there could be some research with valuable information in the gray literature or non-peer-reviewed sources.

The search terms in both search queries were determined by the agreement of all authors and based on a brief literature review. We deliberately conducted a broad search strategy to include as many studies as possible.

In the first search, we used the search query ((type 2 diabetes) OR T2D) AND (oxidative stress) AND (diabetic complications) AND ((genetics) OR (genomics) OR (genes) OR (polymorphism) OR (genetic variations) OR (transcriptomics)) to target genetic association studies and transcriptomic studies focusing on genetic variations in antioxidative enzymes and oxidative-stress-related pathways in T2DM and late complications. We also used the following filters: in the last 10 years, humans. To ensure the accuracy of the gene names in the original papers, we cross-referenced them with the HUGO Gene Nomenclature Committee (HGNC) database.

For the second search, we performed a PubMed search with the search query (diabetic complications) AND (antioxidants). We have also applied these filters to the search: randomized controlled trial, meta-analysis, in the last 10 years, humans.

### 2.3. Study Selection

The study selection process was similar in both the first and second searches. No automation tools were used in the study selection process. We conducted two levels of screening, first by title and abstract (Level 1) and then by full text (Level 2), following the predetermined inclusion and exclusion criteria, described in the section about eligibility criteria in Supplementary File S1. Level 1 screening was conducted cooperatively in a meeting with all of the authors who screened titles and abstracts of the articles, retrieved by using the search strategy. Level 2 screening was performed independently by at least two authors, who reviewed and assessed the full text of the relevant articles. Potentially relevant studies in the Level 2 screening were retained, flagged, and then later discussed, along with any other discrepancies between reviewers. We also more profoundly analyzed the exclusion criteria for all of the excluded reports in the Level 2 screening (Supplementary Files S2 and S3).

### 2.4. Data Collection and Extraction

All of the studies obtained by the search strategy and selection process were examined to extract details regarding basic publication metadata and vital data about research methodology, results, and key findings. All the data extraction and collection were performed independently by one reviewer and then assessed by both the remaining reviewers to warrant exactness (Supplementary File S1).

### 2.5. Risk of Bias Assessment

We conducted a risk of bias assessment for the studies obtained by the second search and selection process. The risk of bias was analyzed using the Cochrane Risk of Bias tool (RoB2) for randomized controlled studies. Each study was assigned a judgement of (a) low risk of bias, (b) some concerns, or (c) high risk of bias for each of the five categories included in the RoB2 tool [36]. Those categories are (1) risk of bias arising from the randomization process, (2) risk of bias due to deviations from the intended interventions, (3) missing outcome data, (4) risk of bias in the measurement of the outcome, and (5) risk of bias in the selection of the reported result. Based on the risk of bias in the named categories, the overall risk of bias was assessed for each study. The risk of bias was conducted independently by two reviewers, and any discrepancies in assessment were resolved with consensus. No automation tools were used in the risk of bias assessment.

### 2.6. Data Synthesis Methodology

As both searches varied greatly in their purpose and the retrieved studies were extremely non-homogeneous regarding the type of late complications, used intervention, and measured outcomes (different even amongst the same type of late complication), we did not conduct a meta-analysis and have, therefore, not included summary statistics or effect size estimates in this systematic review. We carried out narrative data synthesis instead, grouping studies according to the experimental approach (genetic variability, gene expression studies; first search) and antioxidants that were used as an intervention (second search). We present the crucial study characteristics, methodology, and findings in the main text and summary tables. As the main text narrative reflects the results of the first and second searches, we have designed the headings and subheadings accordingly.

## 3. Results

We carried out two searches with different intentions. For the genomic and transcriptomic basis of T2DM late complications (first search), the PRISMA flow diagram is presented in Figure 1, and for antioxidants as an intervention in T2DM late complications (second search), in Figure 2. With full-text screening, we determined thirty-three studies, focusing on the genomic and transcriptomic basis of T2DM late complications development, and twenty-three interventional randomized controlled trials that focused on the role of antioxidants as a treatment in patients with T2DM late complications. We conducted data extraction and synthesis on these studies.

The studies that were excluded during Level 2 screening are listed in the Supplementary Files (Supplementary File S2 for the first search, and Supplementary File S3 for the second search), along with the exclusion criteria used. Some studies have fulfilled multiple exclusion criteria but we counted each study only once with the exclusion criteria that was first noted by the reviewer.

### 3.1. Genomic and Transcriptomic Basis of T2DM Late Complications—Overview of the Studies’ Characteristics

In our review, we identified 27 genetic association studies focusing on the role of genetic variability in the development of T2DM late complications and 6 transcriptomic studies investigating gene expression levels in T2DM late complications that fulfilled our eligibility and exclusion criteria.

The characteristics and the key findings of the genetic studies are presented in Table 1 and those of the transcriptomic studies in Table 2.

The eligible genetic association studies were conducted in different world regions and populations. Most of them included Caucasian subjects from Europe (n = 13) or from India (N = 4), Iran (n = 3), and Pakistan (N = 1). With regards to European countries, five studies were performed in Slovenia, two in Greece, two in Poland and there was also one study per country from France, Romania, Turkey, and Russia. With regards to East Asia, two eligible studies included Japanese subjects, one included Korean, and one Han Chinese. Of the other world regions, one study included Tunisian, and one study included Brazilian subjects. The majority of the studies were of a moderate or small size. Only four studies included more than 1000 participants: a French study that focused on SOD3 locus included 3137, a Japanese study that focused on candidate antioxidative genes included 1977, a Russian study that focused on RAC1 included 1470, and a Brazilian study that focused on AKR1B1 included 1005 subjects. The majority of the other studies included between 400–999 subjects (N = 9) or between 200 and 399 subjects (N = 8), mostly further divided into patients and controls. Only six studies included fewer than 200 subjects.

Most of the studies focused on microvascular T2DM complications (n = 15), namely, DKD (n = 6), DR (n = 4), and diabetic peripheral neuropathy (n = 3); one study included any microvascular complication and one study focused on albuminuria only. The studies focusing on macrovascular T2DM complications (n = 8) investigated PVD (mainly DFU) (n = 2), one study investigated risk factors for the development of any macrovascular complication, and one looked for associations with the thickness of carotid plaques.

Only one study used the whole genome data, although they extracted only the information on 1449 SNPs from the oxidative-stress-related pathways. Among the other studies, only 2 investigated from 6 to 9 genes, while 11 studies investigated from 2 to 5 genes, and 13 studies focused on a single candidate gene, investigating either one common polymorphism only (N = 8) or more than one polymorphism (N = 5) within the candidate gene or gene locus. The genes studied most frequently were the ones coding for antioxidative enzymes: SOD isoenzymes (N = 6), GPx isoenzymes (N = 4), CAT (N = 3), thioredoxin system (N = 2); glutathione metabolism: GSTs (N = 6), GCLM (N = 2), GCLC (N = 1); enzymes involved in the generation of ROS and RNS: NOS3 (N = 4), MPO (N = 2), and CYBA (N = 3); and proteins involved in DNA repair: XRCC1 (N = 3), XRCC3 (N = 1), PARP1 (N = 1). It needs to be pointed out that a total of 20 genes were investigated in only a single study. Furthermore, 16 studies included only genotype information, while only a few studies also provided information on plasma or urine levels of biomarkers of oxidative stress (N = 5), antioxidative enzyme levels (N = 4), or both (N = 2).

Among the six transcriptomics studies that fulfilled all the inclusion criteria, two studies were conducted with subjects from India, while other studies were conducted in different regions of the world: Egypt, Mexico, Saudi Arabia, and Spain. Only one (Indian) study investigated associations with both microvascular and macrovascular T2DM late complications, while the other five studies focused on microvascular complications only: two on DKD, two on DR, and one on albuminuria. All the eligible studies investigated gene expression in peripheral blood (PB) and used either whole blood (N = 3) or peripheral blood mononuclear cells (PBMCs) for RNA extraction. Only one study employed microarray expression analysis to investigate the expression levels of more than 50,000 unique mRNA and long non-coding RNA (lncRNA) transcripts and validated the most differentially expressed mRNAs using quantitative PCR (qPCR). Two studies analyzed the expression levels of three transcripts; however, only one of them used qPCR, while the other used a semiquantitative analysis of PCR amplicons on the agarose gel, thus, leading to a high risk of bias in interpreting the results of the study. Two studies analyzed the expression level of only one transcript; both used qPCR. Two studies were limited to transcriptome analysis, while three studies correlated transcript levels with plasma or urine levels of biomarkers of oxidative stress, and one study correlated the transcript levels with the respective protein levels.

### 3.2. Antioxidants as an Intervention in T2DM Late Complications—Overview of the Studies’ Characteristics

In our review, we identified 23 studies focusing on the role of antioxidants as a treatment in T2DM late complications that fulfilled our eligibility and exclusion criteria. The basic characteristics of the reviewed studies are presented in Table 3.

The acquired studies were conducted in 8 different countries but mainly in Iran (n = 12), Mexico (n = 3), Malaysia (n = 3), and Korea (n = 2). There was also one study per country from Egypt, China, and Greece. The studies also varied in duration, from short-term interventions of 3 weeks to extended monitoring of up to 12 months. Only one of the studies lasted fewer than 8 weeks with the majority of the studies lasting 8–14 weeks. Most of the studies precalculated the needed sample size estimation. Some studies had as few as 25 participants, while the largest number of participants was included in the study by Essmat et al. (194 participants) [74]. The majority of the studies had an average range between 40 and 80 participants (n = 15), that were divided into two or rarely more groups.

Most of the studies focused on microvascular T2DM complications (n = 18), namely, diabetic peripheral neuropathy (n = 9), diabetic kidney disease (n = 6), and *DR* (n = 2). The studies researching diabetic peripheral neuropathy mainly focused on symptoms of pain, nerve conduction, and peripheral neuropathy and not on autonomic neuropathy. In *DR*, both studies included patients with *NPDR*. The studies focusing on macrovascular T2DM complications (n = 6) have investigated *PVD* (mainly *DFU*) (n = 3), *CVD* (coronary heart disease) (n = 2), and cerebrovascular disease (acute cerebral infarction) (n = 1). The most often used outcome measures were anthropometric, metabolic, and biochemical parameters, addressing characteristics of T2DM complications or biomarkers of antioxidant stress. Some of the used outcomes, focusing on oxidative stress (directly or indirectly), are levels of applied antioxidant, total antioxidant capacity (TAC), prooxidant–antioxidant balance (PAB), uric acid levels, changes in superoxide dismutase (SOD), malondialdehyde (MDA) levels, nuclear factor erythroid 2-related factor 2 (Nrf2) levels, microRNA levels, homocysteine levels, glutathione peroxidase (GPx) activity, glutathione reductase activity (GR), serum tumor growth factor β (TGF-β) levels, VEGF-A levels, nerve growth factor (NGF) levels, thromboxane B2 (TXB2) levels, C-reactive protein (CRP) levels, vascular cell adhesion molecule 1 (VCAM-1) levels, and tumor necrosis factor receptor 1 (TNFR-1) levels.

The studies implemented carotenoids (crocin), vitamins and their derivatives (vitamin E, vitamin C, tocotrienol-rich vitamin E from palm oil (Tocovid), coenzyme Q10 (ubiquinone)), polyphenolic compounds (flavonoids, green tea extracts, grape seed proanthocyanidin extracts, resveratrol, curcumin, nano curcumin), fatty acids (α-lipoic acid, γ-linolenic acid, omega-3 fatty acids from flaxseed oil), melatonin, and well-described antioxidant combinations.

#### Risk of Bias Assessment

**Figure 3:**
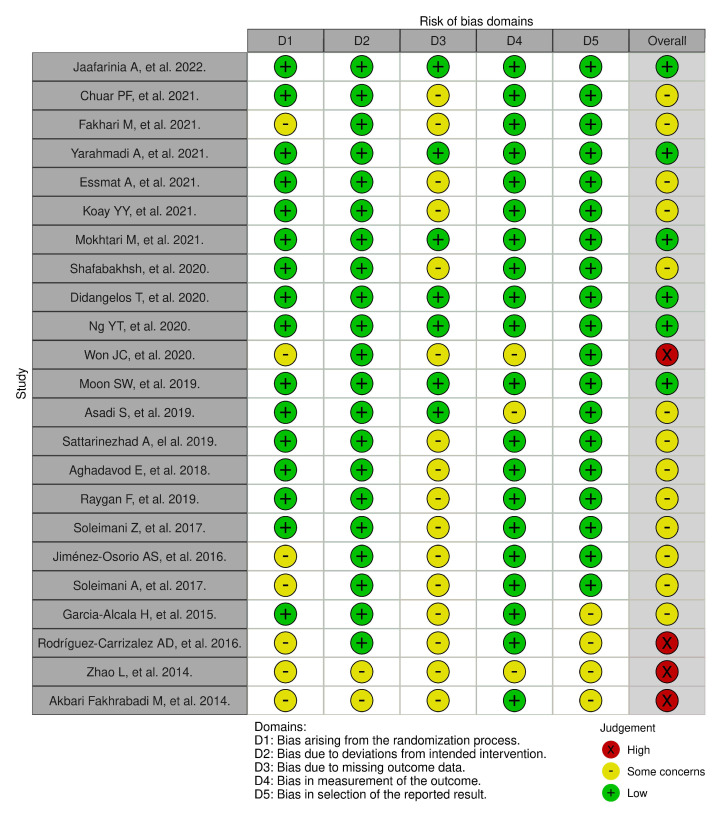
Risk of bias assessment (second search) [70,71,72,73,74,75,76,77,78,79,80,81,82,83,84,85,86,87,88,89,90,91,92,93].

We assessed the risk of bias for the studies, researching antioxidants as an intervention in T2DM late complications (second search), as described in the methodology section, using the Cochrane RoB2 tool. A detailed summary of the risk of bias is available in Figure 3. We also provide the overall risk of bias in the summarization of Table 3. The consent between researchers evaluating the risk of bias was more than 80% and any discrepancies have been discussed and resolved. There were a few studies that received a “high” overall risk of bias rating (n = 4). A significant number of studies were given “Some concerns” overall risk of bias (n = 13), and some studies received “low” overall risk of bias (n = 6).

## 4. Discussion

### 4.1. Genetic Background of T2DM Late Complications

Genetic variability may determine the balance between the ROS and RNS-producing systems, antioxidant systems, and tissue-related factors and repair mechanisms. The genetic variability in genes involved in these mechanisms was the focus of genetic association studies focusing on individual candidate genes or a small number of genes in these candidate pathways. Although some of the studies investigated the associations of genetic polymorphisms in genes coding for the enzymes generating ROS and RNS as part of their physiological function or as metabolic by-products, such as *MPO*, *CYBA*, and *NOS3*, the majority of the studies focused on the genetic factors determining the capacity of antioxidant defense systems. Among them, genetic variants in *SOD*, *CAT*, and *GPX* were the most frequently studied, followed by the enzymes involved in glutathione metabolism and thioredoxin redox system. Tissue-related genetic factors and genetic variability in other genes involved in antioxidative and inflammation-related pathways, polyol and hexosamine metabolism, as well as the formation of advanced glycosylation end-products (AGEs), were more or less the subject of only individual studies.

#### 4.1.1. Genetic Variability in Antioxidant Systems and T2DM Late Complications

SOD, CAT, and GPX constitute the first-line defense system against ROS, thus, having an indispensable role in the antioxidant protective capacity of biological systems from oxidative stress. SOD converts the superoxide radical into hydrogen peroxide, while CAT and GPX break down hydrogen peroxide and hydroperoxides into harmless molecules of water and alcohol. GPX also plays a crucial role in converting lipid peroxides to their corresponding alcohols, mainly in the mitochondria and sometimes in the cytosol, thus, inhibiting the lipid peroxidation process.

SOD has three distinct forms, encoded by different genes. *SOD1* gene codes for cytosolic Cu/Zn-SOD, *SOD2* gene codes for mitochondrial MnSOD, and *SOD3* gene codes for extracellular Cu-Zn SOD. SOD3 is expressed in most tissues and is considered the major enzymatic antioxidant defense against vascular and cardiovascular diseases. The genetic variability in these genes is known to be associated with decreased gene expression levels and/or enzyme activity and results in a decreased capacity of antioxidative defense [94,95].

Despite their primary role in superoxide scavenging, *SOD1* and *SOD3* polymorphisms were not found to play a major role in the development of late T2DM complications. Although decreased plasma SOD1 and GPX1 levels were reported in T2DM patients with DSPN, no association was observed between common functional genetic polymorphisms in these genes and DSPN risk [38]. Similarly, *SOD2* Val16Ala polymorphism was not found to play a major stand-alone role in protection against oxidative stress or the development of late complications of T2DM despite its crucial role in primary defense against mitochondrial superoxide radicals [40]. However, when *SOD2* Val16Ala was included in the composite 5 polymorphic gene score, along with *GCLM* C-588T, *NOS3* G894T, *CYBA* C242T, and *MPO* G-463A, carriers of ≥8 pro-oxidant alleles had a significantly increased risk for coronary heart disease (CHD) compared to the carriers of <8 pro-oxidant alleles; this association was found to be significant with or without adjustment for patients clinical characteristics [39]. The protective effect of *SOD2* T47C (Val16Ala), but not *SOD1* or *CAT* polymorphism, against the development of late complications was observed also in Tunisian T2DM patients but only in a subset of patients who consumed more than three cups of green tea per day. The interaction between *SOD2* T47C (Val16Ala) polymorphism and polyphenol consumption was confirmed also by the detection of significantly higher urinary polyphenol derivatives (UPDs) among individuals consuming more than three cups of tea per day who were *SOD2* 47 CC genotype carriers compared to non-carriers [41].

Positive findings were reported also in a large study that investigated several polymorphisms within the SOD3 gene locus in 3137 French T2DM patients from the DIABHYCAR study cohort and found the association of polymorphic *SOD3* rs2284659 T allele with decreased incidence of myocardial infarction, as well as with cardiovascular and total mortality during the 5-year follow-up period [37].

Common polymorphisms in antioxidant genes (*SOD2*, *CAT*, *GPX1*, *GSTP1*, *GSTM1*, *GSTT1*, *GCLC*, and *GCLM*) were investigated also in a small cohort of clinically well-defined Slovenian T2DM patients, with *CAT* rs1001179 and *GSTP1* rs1138272 showing the strongest association with the risk for end-stage kidney failure, while *GSTP1* rs1695 and *GSTP1* haplotypes influenced the risk of moderately increased albuminuria [40].

*GPX1* polymorphism resulted in decreased GPX1 protein levels but was not found to be associated with DSPN in T2DM patients [38]. This polymorphism was also not associated with either macrovascular or microvascular complications in Slovenian T2DM patients [40].

The availability of GSH as a substrate for GPX depends on the activity of other enzymes that use glutathione, such as glutathione S-transferases (GSTs), and the rate of GSH biosynthesis. GSH is synthesized in two steps that are catalyzed, successively, by two polymorphic enzymes: γ-glutamylcysteine synthetase (glutamate-cysteine ligase, GCL) and GSH synthetase. GCL is the rate-limiting enzyme in glutathione biosynthesis and genetic polymorphisms in its catalytic (GCLC) and modifying (GCLM) subunits were associated with increased susceptibility to oxidative stress and with increased MI risk in the general population and T2D patients [96]. Although no associations with either *GCLC* or *GCLM* polymorphisms as stand-alone risk factors for T2DM complications were observed within Slovenian T2DM patients [40], in Japanese patients, *GCLM* C-588T polymorphism was included in the composite 5 polymorphic gene score associated with increased risk for CHD [39]. The availability of glutamate for GSH synthesis may be also modulated by genetic variability in other enzymes that use glutamate as a substrate, such as glutamate–ammonia ligase (GLUL, also known as glutamine synthetase (GLNS)) which converts glutamate to glutamine. A Pakistani study observed an association of *GLUL* rs10911021 with CHD among patients with T2DM but not among the non-diabetic CHD patients. Furthermore, *GLUL* polymorphism was also associated with plasma levels of oxidative stress markers. Namely, in the carriers of polymorphic *GLUL* allele, the plasma levels of MDA and GSSG increased, while GSH levels decreased depending on the number of polymorphic alleles [44].

GSTs are using GSH in a variety of conjugation reactions that also include the detoxification of xenobiotics and secondary metabolites of ROS. *GSTM1*, *GSTT1*, and *GSTP1* polymorphisms were not associated with DN in Romanian T2DM patients [45]. Neither were the *GST* polymorphisms associated with any microvascular complications among Slovenian T2DM patients, while *GSTP1* rs1138272 showed a nominal association with the risk for end-stage renal failure due to DKD. Furthermore, in T2DM patients without end-stage renal failure, *GSTP1* rs1695 and predicted GSTP1 activity were nominally associated with the development and severity of albuminuria [40].

Thioredoxins (TXNs) and their corresponding thioredoxin reductases (TXNRDs) constitute another important system for ROS scavenging. TXNs serve as electron donors for the reduction of H_2_O_2_ and a variety of other substrates and are regenerated by their respective NADPH-dependent thioredoxin reductases (TXNRD). Thioredoxin-1 (TXN1) and thioredoxin reductase-1 (TXNRD1) are primarily localized in the cytosol, while thioredoxin-2 (TXN2) and thioredoxin reductase-2 (TXNRD2) are both expressed in the mitochondria [97,98]. In Slovenian T2DM patients, one study reported the association of *TrxR2* rs4485648 polymorphism with increased risk for DR [51], while the other study reported a decreased risk for MI in carriers of *TXNRD2* rs1548357 CC + CT genotypes [52].

Another antioxidant enzyme studied with regards to a potential role in the development of T2DM complications is NAD(P)H dehydrogenase, quinone 1 (NQO1), a cytoplasmic enzyme that catalyzes the reduction of quinones to hydroquinones by using NADH as an electron donor, thus, increasing intracellular NAD^+^ levels. It was investigated in only one of the eligible studies, suggesting that the NQO1*2 allele is associated with a higher risk for DKD and lower plasma NQO1 levels in Indian T2DM patients [50].

Heme oxygenase 1 (*HMOX1* or *HO-1*) is another gene investigated in a single study. It codes for the rate-limiting enzyme in heme catabolism and has an important role in reducing oxidative stress levels at minimizing the oxidative damage carried out by the heme molecule. The *HMOX1* -413 TT genotype, but not the promotor (GT)n microsatellite repeat, was associated with albuminuria, especially in patients with longer T2DM duration and poor glycemic control [53].

#### 4.1.2. Genetic Variability in ROS-Generating Enzymes and T2DM Late Complications

ROS and RNS are generated as products of physiological metabolic reactions; however, their production may increase in response to particular stress conditions.

Membrane-bound NADPH oxidase (NOX) is one of the major sources of superoxide anion in phagocytes and vascular endothelial cells. The polymorphic cytochrome b-245 alpha chain gene (*CYBA*) encodes the alpha subunit, also known as the light chain of cytochrome b(-245), or p22-phox, while *CYBB* gene encodes the cytochrome b beta subunit, also known as the heavy chain or p91-phox component of NOX2. *CYPBA* polymorphism was not associated with the risk for the development of microvascular T2DM complications in two Indian studies: the first investigated genetic risk factors for DKD [46] and the second the risk for DR [49]. However, in Japanese T2DM patients, *CYBA* C242T polymorphism was included in the composite pro-oxidant gene score for increased coronary heart disease (CHD) risk [39].

Another ROS-producing enzyme, encoded by a polymorphic gene that was also included in the composite pro-oxidant risk score [39], is myeloperoxidase (MPO), which produces hypochlorous acid (HOCl) in the lysosomes of monocytes and neutrophil cells. In a large group of Han Chinese T2DM patients, *MPO* rs2107545 polymorphism was associated with an increased risk of carotid plaques [42].

Lipoxygenases (LOXs) are another family of enzymes with an important role in the oxidative metabolism of polyunsaturated free fatty acids, such as arachidonic acid, and are also involved in oxidative stress, inflammation, and atherosclerosis. In humans, LOX12 and 15 mainly metabolize (AA). Three polymorphic enzymes from the LOX family were investigated in relation to macrovascular complications of T2DM. *ALOX12* rs14309 was associated with an increased risk for MI and CV events, as well as an increased risk for cardiovascular and overall mortality in a small group of Greek T2DM patients [43]. On the other hand, polymorphisms in genes coding for arachidonate 5-lipoxygenase (*ALOX5*) and arachidonate 5-lipoxygenase activating protein (*ALOX5AP*) were not found to be associated with the increased risk of carotid plaques in a large cohort of Chinese T2DM patients [42].

#### 4.1.3. Genetic Variability in RNS Generating Enzymes and T2DM Late Complications

Nitric oxide (NO) can have a double role and can result in antioxidative and vasoprotective effects or pro-oxidant and pro-inflammatory action. It is synthesized from L-arginine by three isoforms of nitric oxide synthase (NOS). Neuronal NOS (NOS1) and endothelial NOS (NOS3, also known as eNOS) are expressed constitutively and regulated by the interaction of Ca^2+^ with calmodulin. NOS2 (iNOS) is inducible and expressed at a higher level in response to infection or inflammation. NO produced by NOS3 has an important vasoprotective role as it causes smooth-muscle relaxation and inhibition of platelet and leukocyte adherence and aggregation to the vascular endothelium. Furthermore, it helps to reduce oxidative stress by inhibiting lipid peroxidation and by scavenging superoxide anion. Three small studies investigated *NOS3* polymorphisms in relation to different T2DM complications with inconclusive results: a study in Iranian patients observed a protective effect of the *eNOS* 298T allele against DFU [48], a study in Indian T2DM patients observed no association with the risk for DR [49], while another Indian study observed association with increased risk for DKD [46]. However, in Japanese patients, *NOS3* polymorphism was included in the composite 5 polymorphic gene score associated with increased risk for CHD [39].

#### 4.1.4. Genomic Approaches in Elucidating the Genetic Basis of T2DM Late Complications

As clearly shown above, candidate gene studies have an important limitation as both T2DM and its late complications are complex traits, and the contribution of each genetic factor is rather small. These limitations of the candidate gene approach may be overcome by genome-wide association studies (GWAS), which allow for the analysis of a large number of polymorphisms across the entire genome but require large study cohorts to reach statistical significance. We retrieved only one study that performed a chip-based whole genome genotyping, albeit in a relatively small sample of 131 Greek T2DM patients with DKD and 220 T2DM patients without DKD. However, in the next step, the data analysis followed a candidate gene approach and focused on 1449 SNPs in 94 genes in the oxidative-stress-related pathways. The study reported that 43 SNPs in 21 genes were associated with DKD. Some of these SNPs were previously associated with DKD, but several novel associations that need to be still validated were also reported. Genetic polymorphisms in genes coding for secreted phosphoprotein 1 (SPP1), thyroid peroxidase (TPO), copper chaperone for superoxide dismutase (CCS), clusterin (CLU), arachidonate 12-lipoxygenase (ALOX12), interaction protein for cytohesin exchange factors 1 (IPCEF1), oxidation resistance 1 (OXR1), aldehyde oxidase 1 (AOX1), nitric oxide synthase 3 (NOS3), and in prion protein (PRNP) conferred protection against development of DKD. On the other hand, some of the polymorphisms in genes coding for TPO, titin (TTN), Shugoshin 2 (SGO2); NOS3; PDZ and LIM domain 1 (PDLIM1), CLU, glutathione peroxidase 4 (GPX4), thioredoxin reductase 2 (TXNRD2), epoxide hydroxylase 2 (EPHX2), metallothionein-like protein 5 (MTL5), eosinophil peroxidase (EPX), glutathione peroxidase 3 (GPX3), soluble glutathione serine transferase alpha 7Ρ (GSTA7P), glutathione peroxidase 6 (GPX6), OXR1; and glutathione S-transferase A4 (GSTA4) conferred increased risk for DKD [47].

#### 4.1.5. Transcriptome and Gene Expression Studies in Patients with T2DM Late Complications

The interplay of metabolic changes, oxidative stress, as well as the resulting inflammatory and tissue response may lead to increased or decreased gene expression, thus, adding an additional level to the genetically determined interindividual variability in the risk of developing T2DM late complications. Transcriptomics enables the identification and relative quantification of a large number of transcripts in a given sample compared to gene expression analysis using quantitative PCR (qPCR) approaches that enable relative quantification, or digital PCR (dPCR) that also enables absolute quantification, of individual transcripts.

Our literature search retrieved only one study that performed microarray-based transcriptome analysis in RNA samples extracted from peripheral blood (PB) of 15 Mexican patients with T2DM, 15 patients with DKD, 15 prediabetic patients, and 15 controls. Only six samples per group were used for the analysis on the microarray that covered more than 26,800 unique gene transcripts and over 30,000 long noncoding RNAs (LncRNAs). A total of 351 genes were differentially expressed (345 up-regulated and 6 down-regulated) in the group of DKD patients compared to the group of T2DM patients. The fourteen most differentially expressed genes were analyzed by qPCR in the entire study group, but the qPCR results were concordant with the microarray data only for four genes: phosphofructokinase (*PFKL*), coding for the most important glycolytic enzyme; cyclin B1 (*CCBN1*), involved in the regulation of cell cycle; caspase 2 (*CASP2*), involved in proteolysis and apoptosis; and methyltransferase 22, KIN27 lysine (*METTL22*), a non-histone lysine methyltransferases involved that can be activated by oxidative-stress-related DNA damage. The authors have proposed that the expression signature of these four transcripts could be used for early-stage diagnosis of DKD before the onset of albuminuria; however, these results need to be validated in a much larger study [62].

The other study that investigated gene expression profiles in Indian T2DM patients with DKD was only slightly larger and included 30 patients with T2DM, 30 patients with DKD, and 30 healthy controls. They have shown higher expression levels of 9 out of 10 candidate genes related to oxidative stress, endoplasmic reticulum (ER) stress, and crosstalk between these processes in both T2DM and DKD patients when compared to controls. The authors have validated their results in a cell-culture system, confirming the cross-talk between oxidative stress and ER stress in patients with DKD [65].

Two other studies focused on the expression profiles of candidate genes in patients with DR. A Spanish study investigated the expression levels of MMP9, related to extracellular matrix remodeling; SLC23A2, involved in the transport of antioxidant vitamins; and MMP9, related to extracellular matrix remodeling as potential blood biomarkers of DR. Their study included 14 T2DM patients with DR, 35 T2DM patients without DR, and 32 as control. Although MMP9 was up-regulated and SLC23A2 down-regulated in T2DM patients when compared to controls, the expression profiles were not specific for patients with DR [63].

T2DM patients with (N = 22) and without DR (N = 45) were also included in a Saudi Arabian study that investigated the expression levels of three major antioxidative enzymes: SOD, GPX, and CAT. Their semiquantitative analysis of qPCR products on agarose gel that suggests lower expression levels of these enzymes in DR patients may be biased, but they confirmed their observation with the analysis of the respective plasma protein levels [65].

An Egyptian study investigated NLRP3 expression levels in peripheral blood in T2DM patients with normo-, micro-, and macroalbuminuria and healthy controls (15 subjects per each group) and correlated them with the serum marker of oxidative-stress-related DNA damage 8-OHdG, a marker of inflammation IL1B and urinary heat shock protein 72 (uHSP72), and found them all increased in macroalbuminuric T2DM patients when compared to the other groups [66].

Another study focusing on a single gene transcript, albeit correlating the data with the serum levels of oxidative stress markers, investigated the expression levels of advanced glycosylation end-product-specific receptor (RAGE) in relation to T2DM micro and macrovascular complications. They reported increased AGER expression levels in T2DM patients with late complications when compared to T2DM patients without vascular complications and controls, Furthermore, the circulating AGE levels showed a significant positive correlation with RAGE m-RNA expression and oxidative stress markers.

#### 4.1.6. Genetic and Transcriptomic Biomarkers of T2DM Late Complications—Commentary on Studies’ Characteristics and Limitations

The last decade has not brought much progress into the elucidation of genomic and transcriptomic biomarkers of T2DM late complications. Despite the rapid progress in omics technologies, we retrieved only one eligible study that used the GWAS approach and one study that analyzed transcriptome using microarrays. Most of the studies focused on candidate genes only, both in genetic and gene expression analyses. The study groups were often of medium size for genetic studies, there were only three studies with more than 1000 subjects included. For transcriptomic studies, the sample sizes were predominantly small. The largest limitation of most of the eligible studies is, however, a lack of validation in independent patient groups or other populations. This may be particularly problematic in genetic variability studies as differences in genetic background may influence the genotype distributions. Another limitation is the lack of replication, both in genetic and gene expression studies. In genetic studies, only the classic antioxidant enzymes were investigated in more than one study, but often in relation to different late complications, and often the key findings were not consistent between studies. Therefore, neither the so-far investigated genetic variants, nor the investigated gene transcript levels can be used as predictive or prognostic biomarkers at this stage.

#### 4.1.7. Future Perspectives on Genetic and Transcriptomic Studies of T2DM Late Complications

In the last two decades, the establishment of international consortia and biobanks enabled large GWAS studies that have so far identified more than 400 variants associated with the risk of T2DM in different ethnic groups [99]. Furthermore, with the rapidly increasing clinical implementation of next-generation sequencing (NGS), approaches such as whole-exome sequencing (WES) and whole-genome sequencing (WGS) could also be used to improve our understanding of the genomic basis of T2DM late complications. While the majority of T2DM risk variants detected by GWAS were located in intergenic and intronic regions, and the coding variants had to be imputed by bioinformatic analysis, WES has the advantage of being able to detect variants in the coding regions. Furthermore, it has been shown that the WES approach enables the identification of coding variants associated with T2DM risk also in small populations [100]. However, additional effort is needed toward obtaining better clinical and phenotypic information before GWAS or WES data can be used to reveal the genomic basis of T2DM late complications.

Another rapid development that could support multi-omics approaches in investigating the basis of T2DM late complications is the research into extracellular vesicles (EVs). EVs are membrane-bound structures that are released from many different cell types and serve as mediators of cell-to-cell communication. EVs are heterogeneous in size, subcellular origin, and molecular composition. EVs transport different molecules such as nucleic acids (miRNA, mRNA, and DNA), proteins, and metabolites characteristic of their cells of origin on their surface and in their lumen, accumulate in all body fluids to high concentration, are often dysregulated in various diseases, and can be enriched from plasma or urine for biomarker discoveries [101]. Furthermore, EVs present an enriched source of potential genomic, transcriptomic, and metabolomic biomarkers that can be analyzed by various multi-omics approaches. Such approaches were already used in two pilot studies that, however, require further validation. The first study investigated EVs in paired plasma and urine samples from five patients with T2DM and DKD and four healthy subjects and found 13 common differentially expressed EV miRNAs in both fluids. The bioinformatic analysis of target mRNAs supported by the measurement of urine metabolite levels in a larger validation study led to the suggestion that urinary succinate and adenosine levels could be used as a non-invasive biomarker of DKD [102]. In another pilot study that included eight T2DM patients with DKD and eight controls without T2DM, EVs were isolated from the urine, and their miRNA content was sequenced by NGS. The bioinformatic analysis of the most differentially expressed miRNA predicted the involvement of molecular processes related to inflammation and apoptosis in the development of DKD [103].

### 4.2. Antioxidants as an Intervention in T2DM Late Complications

#### 4.2.1. Dietary Antioxidants and Lifestyle

A diet rich in antioxidants improves glycemic status, lowers oxidative stress biomarkers, and raises serum levels of antioxidant enzymes in T2DM patients.

##### Polyphenols

Polyphenols are natural compounds, namely, secondary metabolites of plants, found primarily in fruits like grapes, pear, and berries, vegetables, grains, and beverages, such as red wine and a cup of tea, which contain approximately 100 mg of polyphenols. They can be classified based on the number of phenol rings they contain and the structural elements that connect these rings. The group of polyphenols contains various subgroups, such as phenolic acids, flavonoids, stilbenes, and lignans. The majority of the polyphenols are absorbed in the gastrointestinal tract. There are large seasonal differences in the consumption of polyphenols between countries, as in those that have all four seasons, a higher consumption of fruits and vegetables was observed, especially in spring and summer [104,105].

Almost ten years ago, it was found that cocoa beans and their derivatives are rich in flavonoids, which can scavenge free radicals, reduce ROS production, strengthen the endogenous antioxidant defense system, and improve antioxidant equilibrium. Moderate and regular exercise can be considered good antioxidants with up-regulation of antioxidant enzymes. It reduces oxidative stress and improves glycemia. One of the exercises that represents the balance between body and mind is Pilates, which can, as a form of exercise, lead to improvement in joint movement, balance, and postural control. A combination of Pilates and cocoa in females with T2DM and DPN can lead to an increase in TAC [72].

In patients with mild to moderate DPN after 16 weeks of treatment with green tea extract, the clinical picture and neurophysiological characteristics were notably improved. Three mechanisms can explain the improvement of DPN. Due to the similar insulin metabolism in the peripheral sensory nerves compared to other tissues, green tea extract had a positive effect on insulin resistance and fasting insulin levels in those with baseline elevated values. Furthermore, green tea extract significantly lowers serum levels of TC and LDL-C, which is not negligible since it has been proven that dyslipidemia affects the thickness and size of the nerves’ myelin sheath. Moreover, it was also shown that it has an anti-inflammatory effect [74].

Curcumin is another polyphenol from turmeric root with antioxidant properties that nullifies harmful effects of ROS and nitrogen and promotes induction of glutathione reductase, SOD, catalase, and GPx. Additionally, curcumin inhibits ROS-generating enzymes such as xanthine hydrogenase/oxidase and lipoxygenase/cyclooxygenase and it may bind redox-active metals, thus, preventing reactions that generate radicals when metal ions are present, resulting in a reduction of oxidative damage. Nanocurcumin, which has better bioavailability than curcumin, may in a brief time reduce the severity of diabetic sensorimotor polyneuropathy [82]. In T2DM patients with diabetic foot ulcer (DFU), supplementation with nano curcumin (80 mg/day) has resulted in a significant improvement in serum levels of the total- and LDL-cholesterol, and TAC and glutathione (GSH) levels; however, it did not improve nitric oxide (NO) and malondialdehyde (MDA) levels [76]. Since curcumin has so much potential in the treatment of patients with T2DM, another trial was planned and carried out. They found that T2DM patients with 2- and 3-vessel coronary artery disease (CHD), diagnosed with angiography, who consumed 1000 mg of curcumin/day for 12 weeks also improved their TAC, GSH, and MDA levels [77]. Nevertheless, there was no effect of curcumin (350 mg/day for 8 weeks) on the activity of the antioxidant enzymes in T2DM patients with diabetic proteinuric chronic kidney disease; however, it did enhance the TAC in this group of patients [88].

Another type of plant flavonoids is proanthocyanidins. Grape seed skin extracts are one of the commercially accessible oligomeric proanthocyanidins, which have a few very interesting effects, among others, also antioxidant and radical scavenging activities. One of the trials found that after 12 months of treatment with grape seed proanthocyanidin extract (GSPE) (150 mg/day), hard exudates severity decreased in 43.9% of T2DM patients with mild to moderate NPDR with a macular thickness less than 300 μm [81].

##### Carotenoids

Crocin is a bioactive ingredient found in the flowers of the Crocus and Gardenia. As a carotenoid, it contains four major components, which are crocin, crocetin, picrocrocin, and safranal. In addition to the direct antioxidative effect, carotenoids also activate Nrf2, a master regulator gene that activates genes, involved in the oxidative stress response [23]. A recent 90-day trial (15 mg of crocin/day) in a group of T2DM patients with microalbuminuria showed that crocin reduced body mass index (BMI) and systolic and diastolic blood pressure. However, among the biochemical and serum parameters, only changes in the serum TG levels were statistically significant. This phenomenon could be explained by inhibition of the absorption of fat and cholesterol and their intensified excretion with feces and probably with pancreatic lipase inhibition [70].

#### 4.2.2. Antioxidant Supplements

Despite the increased availability, purified form, and accurate dosing of modern antioxidants, there is no clear evidence that they can fully reach similar or even greater effects than dietary antioxidants, which consist of a mixture of protective substances.

##### Resveratrol

Resveratrol, which has many different potential effects, such as antioxidant, antiviral, antitumor, and phytoestrogens, could be introduced to standard antihyperglycemic therapy. It belongs to the class of non-flavonoid stilbene derivate polyphenols with a 3,5,4-trihydroxystilbene structure. A variety of fully pigmented fruits and vegetables are sources of resveratrol [106]. Brown et al. shows found that a dose of resveratrol up to 1 g is generally safe [107]. Until recently, only a few small studies about the efficacy of resveratrol on glycemic control were conducted, and the data were very controversial [108]. Nevertheless, studies in the last years, where they used approximately 1000 mg of resveratrol, did not show any decrease in serum creatinine or eGFR. On the other hand, Kumar et al. and Hausenblas et al. trials found that resveratrol, along with pharmaceutical intervention, may reduce serum creatinine. Resveratrol can also significantly increase serum levels of NO, SOD, GSH-Px, and CAT. Consequently, every 1-mmol/l increase in NO was associated with a 4.0-mg/g decrease in urinary albumin excretion [83]. Interestingly, some preclinical studies reported that resveratrol had more protective effects on late complications of T2DM than metformin [109,110,111].

##### γ-Linolenic Acid

γ-linolenic acid (GLA) or 18:3n-6 is an essential omega-6, 18-carbon polyunsaturated fatty acid (PUFA). Although it can be found in seed oils, it can be ingested in the form of dietary supplements. The T2DM patients with DPN treated over 12 weeks with γ-linolenic acid (GLA) manifest changes in terms of pain severity by the visual analog scale and in the total symptom scores. In comparison with α-lipoic acid (ALA), GLA was not inferior regarding the predetermined outcomes [80]. On the other hand, α-linolenic acid from plant sources is part of the omega-3 fatty acids, which also contain eicosapentaenoic acid (EPA) and DHA mainly from fish and fish oils. In T2DM patients with DKD, consumption of omega-3 fatty acid from flaxseed oil (1000 mg/day for 12 weeks) may decrease serum levels of TG and VLDL-cholesterol by influencing cell membrane structure and function, modulation of lipid mediators, and also by regulating the expression of genes related to fatty acids metabolism. Nevertheless, it did not influence oxidative stress [86].

##### Vitamin E

Four isomers such as alpha, beta, gamma, and delta in vitamin E tocopherols and tocotrienols, which are more potent, can be found in grains, nuts, wheat germ, barley, and vegetable oils. A recent trial has shown that administration of oral tocotrienol-rich vitamin E for 8 months has significantly improved serum creatinine and eGFR, despite not affecting urine-to-albumin creatinine ratio (UACR). Its use can be more beneficial for DKD patients with an eGFR of 30–60 mL/min/1.73 m^2^ in 12 months. After 6 months of discontinuation of therapy, values of serum creatinine and eGFR have remained the same [75]. It was demonstrated that a daily administration of 900 mg of synthetic vitamin E for six months leads to a significant enhancement in the median sensory nerve conduction velocity and tibial motor nerve distal latency. Furthermore, supplementation of vitamin E (400 mg) improved neuropathy pain and also reduced physical scores from the RAND-36 questionnaire. Chuar et al. have conducted 12 12-month RCT trials, in which they have shown that vitamin E improves nerve conduction velocities by restoring compromised myelin protective layers around nerve fibers. Nevertheless, they did not show any significant differences in blood TGF-1 levels between placebo and treatment groups [71]. In contrast, a similar trial did not show that tocotrienol-rich vitamin E has any effect on the amplitude of action potentials [79]. A high dose of vitamin E (800 IU) supplementation significantly reduces levels of serum TC, LDL-cholesterol, and the ratio of TC/HDL cholesterol. In addition, it elevates vitamin E and HDL-cholesterol levels and levels of plasma GSH [84].

Platelet-rich plasma fibrin glue (PRP-FG) is an effective wound dressing (used three times per week). If it is combined with oral vitamin E (200 IU/2 days) and C (250 IU/2 days) in patients with non-healing DFU that arises due to DPN and PVD, its administration could significantly improve the wound healing rate. Vitamin C is known as a very strong antioxidant that can preserve the balance between pro- and antioxidants. On the other hand, vitamin E is the most important peroxyl radical scavenger, and its antioxidant activity is related to its ability to act chemically as a free radical that protects cells from oxidative damage caused by ROS. Furthermore, vitamin C can indirectly or directly decrease vitamin E radicals and renew its antioxidant activity after oxidation [73].

The antioxidant coenzyme Q10 with large molecular weight is essential for the effective transport of electrons throughout the mitochondrial respiratory chain and the creation of ATP. It is reduced to ubiquinol in the intestine and later transported by lymph to the circulation, where it has an important role in the protection of lipoproteins from oxidation. In the randomized, double-blind, placebo-controlled trial, they observed high baseline activity of catalase and GPx, but after treatment with coenzyme Q10, the activity of the mentioned enzymes decreased significantly in T2DM patients with NPDR [91]. Supplementation with 200 mg of ubiquinone in 12 weeks has been shown to increase TAC but does not improve symptoms of DPN [93].

##### α-Lipoic Acid

Lipoic acid can extract oxidative stress products such as hydroxyl radicals, singlet oxygen, nitric oxide radicals, and hydroperoxides, as well as regenerate vitamins C, glutathione, etc., to maintain normal antioxidant capacity. Most likely, lipoic acid increases the expression of Nrf-2-mediated antioxidant genes and peroxisome proliferator-activated receptor-regulated genes, hence, improving the antioxidant defense system. Moreover, some of the therapeutic benefits of a medium chain fatty α- lipoic acid (ALA) in the treatment of DPN may be ascribed to its ability to increase blood flow to the nerves via antioxidant processes and to reduce endothelial dysfunction by lowering plasma levels of interleukin 6 and plasminogen activator 1. In combination with (SOD 10 mg, N-acetyl-carnitine 300 mg, and vitamin B12 250 mcg in a single tablet prepared with specific technology, named Multiform Administration Timed Release Ingredients System (M.A.T.R.I.S.^®^), the improvement in BIO, MNSIQ, QL, PAIN, and SNCV, SNAP, and B12 levels can also be seen. Three isoforms, SOD1, SOD2, and SOD3, are metalloenzymes, and their leading mechanism is the neutralization of superoxide and the prevention of the formation of free radicals. Another included substance, n-acetyl-carnitine, has a neurotrophic activity [78]. Because of the limitation factors, related to pharmacokinetics, such as short half-life and 30% bioavailability, the therapeutic efficacy of oral ALA is questionable. However, in the trial from Garcia-Alcala et al., it was shown that a high oral dose of ALA (600 mg for 20 weeks) may decline neuropathic symptoms in patients that respond well to the initial 4-week therapy [90]. In older T2DM patients with acute cerebral infarction, the administration of ALA may significantly decrease MDA levels and increase plasma levels of SOD and GSH-Px. Furthermore, it can significantly decrease TG, TC, LDL-C, and free fatty acids (FFA). ALA may protect against brain cell damage, which can be a result of the elevated levels of oxygen free radicals and lipid peroxidation during cerebral ischemia-reperfusion. Consequently, the NIHSS score exhibits a substantial reduction while the deficiency improved [92].

##### Melatonin

Melatonin suppresses oxidation reactions, catalyzed by ROS, lowering lipid peroxidation. Raygan et al. proved in overweight T2DM patients with 2- and 3-vessel coronary heart disease that supplementation with melatonin (10 mg for 12 weeks) significantly increased plasma levels of GSH, NO, and decreased levels of lipid peroxidation products MDA, protein carbonyl (PCO) and serum levels of hs-CRP. Moreover, melatonin can increase HDL-cholesterol through cholesterol esterification, which is mediated by increased lecithin-cholesterol acyltransferase activity, total-/HDL-cholesterol ratio, and blood pressure. Nevertheless, melatonin did not affect levels of TAC, serum levels of TG, VLDL, TC, and LDL-cholesterol [85].

#### 4.2.3. Antioxidants as Treatment in T2DM Late Complications—Commentary on Studies’ Characteristics and Limitations

The uneven distribution of studies across these countries suggests non-homogeneity in the field of antioxidant research in late T2DM complications. As the majority of the interventional studies were carried out in Iran, this might be due to the particular interest or funding available for this type of research. Nevertheless, this could suggest an imbalance in the available data and potentially diminish generalizability to the other populations due to the differences in genetic background, as well as cultural and dietary characteristics.

Additionally, it is notable that some older interventional studies are rated worse in terms of risk of bias, while some newer studies were less prone to bias. Therefore, we might conclude that the reporting and methodological quality of studies in this research field improved over time. However, the assessment of temporal evolution of methodological quality of research in this field was not the primary objective of this report. Therefore, a more extensive quality assessment with a broader time range would be more appropriate to assess changes in research quality over time.

The majority of the reviewed interventional studies (17 out of 23) focused on microvascular complications (with special emphasis on diabetic peripheral neuropathy and diabetic kidney disease). This might be attributed to several factors. Outcomes, measured in the research of microvascular complications and antioxidants, could be interpreted as easily measurable and quantifiable (for example kidney parameters in diabetic kidney disease or nerve conduction in diabetic peripheral neuropathy) and more independent from other pathogenic mechanisms. Moreover, they are more prevalent and also usually develop before macrovascular complications and are, therefore, an important sign of T2DM progression [112]. In addition, microvascular complications offer a wide range of research dimensions, from symptom management and pain relief to interventions, targeting specific mechanisms. Macrovascular complications (e.g., *CVD*) might be perceived as more challenging to manage with antioxidant intervention when fully developed. Moreover, microvascular complications have a gradual and progressive course, and they may develop over an extended period. This opens the possibility for the duration of interventions, starting with the first symptoms and signs of the disease (e.g., with antioxidant supplements).

The duration of the interventional studies and follow-up and assessment intervals also display heterogeneity as some investigations involved periodic evaluations and others follow-up visits post-intervention. On the other hand, the temporal heterogeneity of the studies opens the possibility of a more nuanced exploration of the impact of antioxidants on diabetes complications over the varying timeframes.

#### 4.2.4. Future Perspectives on the Use of Antioxidant Therapy in T2DM Late Complications

Taking the above into consideration, possible future research might include the use of antioxidants in macrovascular complications and different ethnic backgrounds. From the methodological perspective, the researchers in this field need to pay attention to report missing data in their studies. Moreover, the positive findings should be validated in other studies from the same and different populations. There are also few data available comparing different dosages of antioxidants and different types of them.

Regarding the scope of the studied antioxidants, there might exist a knowledge gap about the use of novel, synthetic antioxidant derivatives. Novel mitochondrial-targeting synthetic antioxidants have been developed recently (e.g., synthetic analogs of coenzyme Q10, idebenone, and mitoquinone). As they have been shown to improve mitochondrial ROS and prevent DNA damage, they might be useful as a therapy in T2DM late complications [113,114,115]. Moreover, the usage of various nanoparticle-based methodologies and encapsulation in food has been shown to improve systemic and local delivery of some antioxidants, namely, polyphenols. However, further sufficiently large clinical studies need to be conducted to validate the safety and efficacy of nano-based antioxidants since the metal- and lipid-based nanoparticles may cause oxidative stress themselves [116,117,118,119,120,121].

It should be also pointed out that although it has been proven that antioxidant therapy may be effective in treating late complications of T2DM, we have to be very cautious when introducing the therapy. We should be guided by the principles of personalized medicine, which means the careful selection of appropriate patients and dosages. Additionally, we need to determine the concentration of, for example, vitamins in the blood or trace elements before incorporating them into therapy. Furthermore, one needs to be aware that antioxidant therapy, like other medications, can also cause side effects.

## 5. Conclusions

The conducted systematic review highlights the important role of oxidative stress in the development of late complications in T2DM patients. The current level of evidence does not support the use of genetic and transcriptomic factors as predictive and prognostic biomarkers for the development of T2DM late complications. Further studies are needed to elucidate the potential of targeting oxidative-stress-related pathways for novel preventative and therapeutic approaches. Antioxidants both in dietary and supplement form have shown improvements in individual metabolic and biochemical parameters. However, due to the relatively short duration of the interventional studies, it is difficult to assess the long-term health benefits of antioxidant supplementation.

## Figures and Tables

**Table 1:** Overview of genetic association studies on oxidative stress and T2DM late complications (first search).

Gene/Biomarker	Participants	Ethnicity	Type of T2D Late Complications	Key Findings	Reference Year
*SOD3* locus: rs2284659, rs2695234, rs17552548, rs758946, rs2270224, rs1799895 (Arg213Gly)	3137 T2DM patients from DIABHYCAR study; 5 year follow-up	French	Macrovascular (MI)	*SOD3* rs2284659 T allele associated with lower incidence of myocardial infarction.	[37] 2015
*GPX1* Pro197Leu, *SOD1* +35A/C, *CAT* -262C/T; SOD1, GPX1 and CAT plasma level	401 T2DM patients: 110 with DSPN, 135 without DSPN, 156 controls without T2DM and DSPN	Polish	Microvascular (DSPN)	No association with DSPN among T2DM patients; GPX1 and SOD1 levels were decreased in T2DM + DSPN patients compared to controls.	[38] 2016
*GCLM* C-588T, *SOD2* Val16Ala, *NOS3* G894T, *CYBA* C242T, *MPO* G-463A	1977 T2DM patients without a history of CVD; 85 had CHD events within 7.5 years (median) follow-up	Japanese	Macrovascular (CHD)	Carriers of ≥8 pro-oxidant alleles had an increased risk for CHD compared to the carriers of <8 pro-oxidant alleles, with or without adjustments for clinical variables.	[39] 2014
*SOD2*, *CAT*, *GPX1, GSTP1*, GSTM1*0, GSTT1*0, *GCLC*, and *GCLM*	181 T2DM patients	Slovenian	Macrovascular and microvascular	*CAT* rs1001179 and *GSTP1* rs1138272 associated with end-stage kidney failure, *GSTP1* rs1695, and *GSTP1* haplotypes associated with moderately increased albuminuria.	[40] 2015
*SOD1* +35A/C, *SOD2* T47C, and *CAT* -21A/T; Plasma MDA; Urine UPDs	366 T2DM patients; consumption of ≥ or < than 3 cups of green tea per day	Tunisian	Macrovascular and microvascular	*SOD2* 47 CC is associated with a lower risk of T2DM complications and higher UPD levels are protective in high tea consumers (>3 cups/day).	[41] 2015
*ALOX5* rs10900213, *ALOX5AP* rs4293222, *GPX1* rs1050450, *GPX3* rs3828599, and *MPO* rs2107545	396 T2DM patients and 678 controls	Han Chinese	Macrovascular (carotid plaques)	*GPX1* rs1050450 and *MPO* rs2107545 are associated with an increased risk of carotid plaques in T2DM patients.	[42] 2015
*ALOX12* rs14309	145 T2DM patients: 108 with DN and 37 without DN	Greek	Macrovascular (MI, stroke, or PAD), CV- and overall mortality	*ALOX12* rs14309 GG genotype is associated with MI, higher cIMT, increased CV events, CV- and overall mortality.	[43] 2018
*GLUL* rs10911021 Plasma levels of MDA, GSH and GSSG	650 CHD patients (275 with T2DM) and 225 controls (30 with T2DM)	Pakistani	Macrovascular (CHD)	*GLUL* rs10911021 is associated with CHD only among patients with T2DM but is associated with MDA, GSH, and GSSG levels.	[44] 2018
*GSTM1*, *GSTT1*, and *GSTP1*	84 T2DM patients: 42 with DN and 42 without DN; 98 healthy controls	Romanian	Microvascular (DSPN)	No association with DN in T2DM patients.	[45] 2015
*NOS3* 894G>T, *CYBA* C242T, *PARP-1* Val762Ala, and *XRCC1*	217 T2DM patients: 55 with DKD and 162 without DKD	South Indian	Microvascular (DKD)	*NOS3* 894T allele associated with increased risk for DKD; *PARP-1* 762Ala allele protected against DKD.	[46] 2014
1449 SNPs in OS pathway analyzed on Illumina Human PsychArray-24 v.1.1 BeadChip	331 T2DM patients: 121 with DKD, 220 without DKD	Greek	Microvascular (DKD)	43 SNPs in 21 genes in the OS pathway associated with DKD: *SPP1*, *TPO*, *TTN*, *SGO2*, *NOS3*, *PDLIM1*, *CLU, CCS*, *GPX4*, *TXNRD2*, *EPHX2*, *MTL5*, *EPX*, *GPX3*, *ALOX12*, *IPCEF1*, *GSTA1*, *OXR1*, *GPX6*, *AOX1*, and *PRN*.	[47] 2021
*NOS3* Glu298Asp	257 T2DM patients: 123 with DFU, 134 without DFU	Iranian	PVD (DFU)	*NOS3* 298T allele protective against DFU.	[48] 2018
*ACE* ins/del, *NOS3* 4a4b, *CYBA* C242T, *PARP-1* Val762Ala, and *XRCC1* Arg399Gln	460 T2DM patients: 149 with DR and 162 with no evidence of DR.	Indian	Microvascular (DR)	*PARP-1* 762Ala allele protected against DR, while *XRCC1* 399Gln allele increased the risk for DR.	[49] 2016
NQO1*2 (rs1800566) and plasma NQO1 levels	400 T2DM patients: 200 with DN and 200 without complications and 200 healthy controls	North Indian	Microvascular (DKD)	NQO1*2 allele is associated with a higher risk for DKD and lower plasma NQO1 levels.	[50] 2016
*Trx2*/*TXNIP* and *TrxR2* rs8140110, rs7211, rs7212, rs4755, rs1548357, rs4485648 and rs5748469	802 T2DM patients: 277 with DR and 525 with no DR	Slovenian	Microvascular (DR)	*TrxR2* rs4485648 polymorphism associated with increased risk for DR.	[51] 2016
*TXNRD2* rs1548357, rs4485648, and rs5748469	972 T2DM patients: 161 patients with MI and 811 patients with no history of CAD	Slovenian	Macrovascular (MI)	*TXNRD2* rs 1548357 CC+CT genotypes are associated with a lower prevalence of MI compared with TT genotype group.	[52] 2015
*HO-1* T(-413)A SNP and (GT)n repeat	536 T2DM patients	Korean	Microvascular (albuminuria)	*HO-1* -413 TT genotype associated with albuminuria compared to A allele carriers, especially with longer T2DM duration and poor glycemic control.	[53] 2015
*ROMO-1* rs6060566	806 T2DM patients: 278 with DR and 528 without clinical signs of DR	Slovenian	Microvascular (DR)	*ROMO-1* rs6060566 CC genotype associated with increased risk for DR.	[54] 2015
*TNF-α*-308 G/C and *IL-1α* -889 C/T; plasma MDA, GSH, SOD, and CAT levels	402 T2DM patients: 188 with DN and 214 without DN; 188 patients with NDN; 235 controls	West Indian	Microvascular (DKD)	*IL-1α* -889 C/T polymorphism may be associated with diabetic nephropathy. MDA was increased, while GSH and SOD were decreased in the patients group compared to controls.	[55] 2015
*NLRP3* rs35829419, *CARD8* rs2043211	181 clinically well-characterized T2DM patients	Slovenian	Macrovascular (MI, PAD, stroke)	*NLRP3* rs35829419 is associated with an increased risk for macrovascular complications, in particular with MI.	[56] 2015
*RAC1* rs4724800, rs7784465, rs10951982, rs10238136, rs836478, and rs9374; plasma H_2_O_2_ levels in 489, and GSSG levels in 258 T2DM patients	1470 T2DM patients; 1007 with and 407 without DR; 553 with and 887 without DKD; 1309 with and 109 without DN; 968 with and 502 without DA; 107 with and 1309 without DFS	Russian	Microvascular (DKD, DR) Macrovascular (DA, DFU)	*RAC1* rs7784465 T/C was associated with increased risk of DR and DA in males, and DR in females. RAC1 rs836478 was associated with DR and DN in males, whereas SNP rs10238136 was associated with DA in females.	[57] 2023
*KEAP1* rs11085735; GSH, GPX, MDA, and TAC	300 T2DM patients: 100 with DR, 100 with DN, 100 without complication; and 100 healthy controls	Iranian	Microvascular (DN, DR)	*KEAP1* rs11085735 AA genotype associated with DN and lower GPX activity compared to CC genotype.	[58] 2022
ALDH2*1/*2 alleles; drinking habits, serum GGT	234 T2DM patients with no DR at baseline; follow-up 5.5 ± 2.5 years	Japanese	Microvascular (DR)	The incidence of DR is significantly higher in the ALDH2*2 allele carriers with a high GGT level than in the non-carriers with a high or low GGT level.	[59] 2013
*AKR1B1* rs759853	1005 T2DM patients: 695 with DKD and 310 patients without DKD	Brazilian	Microvascular (DKD)	A/A genotype was associated with risk for DKD after adjustment for gender, triglycerides, BMI, presence of hypertension and DR, and duration of DM.	[60] 2022
*VDR* ApaI, TaqI, BsmI, and FokI variants, plasma vitamin D, homocysteine, and TBARS levels	262 2DM patients: 127 with DFU and 135 without DFU	Iranian	Macrovascular PVD (DFU)	*VDR* ApaI associated with DFU; ApaI GG and BsmI CC carriers had lower levels of TBARS compared to other genotypes.	[61] 2022
*XRCC3* T241M and *XRCC1* A399G	238 subjects: 116 with T2DM, 50 with DKD, and 72 with normal glucose metabolism	Turkish	Microvascular (DKD)	*XRCC1* Gln allele associated with T2DM and DKD.	[62] 2019
*ADPRT* 726 Val/Ala, *MUTYH* 324 His/Glu, *APE* 148 Asp/Glu; comet assay	355 subjects: 89 with T2DM + DSPN, 120 with T2DM and without DSPN; 146 healthy subjects without T2DM and neuropathy	Polish	Microvascular (DSPN)	None of the 3 polymorphisms associated with the risk of DSPN; *ADPRT* 726 Ala allele increased T2DM risk.	[63] 2015

Abbreviations: ADPRT—poly(ADP-ribose) polymerase 2 (PARP2); AKR1B1—aldo-keto reductase family 1, member B1; ALOX5—arachidonate 5-lipoxygenase; ALOX5AP—arachidonate 5-lipoxygenase activating protein; ALOX12—arachidonate 12-oxidoreductase; AOX1—aldehyde oxidase 1; APE—APEX nuclease 1 (APEX1); CAN—cardiovascular autonomic neuropathy; CARD8—caspase recruitment domain-containing protein 8; CAT—catalase; CHD—coronary heart disease; CCS—copper chaperone for superoxide dismutase; CLU—clusterin; CV—cardiovascular; CVD—coronary vascular disease; CYBA—NAD(P)H oxidase p22phox; DA—diabetic angiopathy; DIABHYCAR—type2 DIABetes, Hypertension, Cardiovascular Events, and Ramipril; DN—diabetic nephropathy; DFU—diabetic foot ulcer; DKD—diabetic Kidney Disease; DPN—diabetic peripheral neuropathy; DR—diabetic retinopathy; DSPN—distal symmetric polyneuropathy; EPX—eosinophil peroxidase; EPHX2—epoxide hydrolase 2, cytosolic; GCL—glutamate–cysteine ligase; GCLC—glutamate–cysteine ligase catalytic subunit; GCLM—glutamate–cysteine ligase modifying subunit; GGT—serum γ-glutamyltransferase; GLUL—glutamate–ammonia ligase (glutamine synthetase—GLNS); GPx—glutathione peroxidase; GPX1—glutathione peroxidase 1; GPX3—glutathione peroxidase 3; GPX4—glutathione peroxidase 4; GPX6—glutathione peroxidase 6; GSH—glutathione; GSSG—glutathione disulfide; GSTA1—glutathione S-transferase A1; GSTM1—glutathione S-transferase M1; GSTP1—glutathione S-transferase P1; GSTT1—glutathione S-transferase T1; GWAS—genome wide association study; HO-1—heme oxygenase 1 (HMOX1); IHD—ischemic heart disease; IL-1α—interleukin 1-alpha; IPCEF1—interaction protein for cytohesin exchange factors 1; KEAP1—Kelch-like ECH-associated protein 1; MDA—malondialdehyde; MI—myocardial infarction; MPO—myeloperoxidase; MTL5—metallothionein-like 5, testis-specific; MUTYH—MutY DNA glycosylase; NDN—non-diabetic nephropathy; NLRP3—NLR family, pyrin domain-containing 3; NOS3—endothelial nitric oxide synthase; NQO1—NAD(P)H dehydrogenase, quinone 1; OS—oxidative stress; OXR1—oxidation resistance 1; PAD—peripheral artery disease; PARP-1—poly(ADP-ribose) polymerase; PDLIM1—PDZ and LIM domain protein 1 (elfin); PRN—thyroid carcinoma, papillary, with papillary renal neoplasia; PVD—peripheral vascular disease; RAC1—RAS-related C3 botulinum toxin substrate 1; ROMO1—reactive oxygen species modulator 1; SOD—superoxide dismutase; SOD1—cytosolic Cu/Zn superoxide dismutase; SOD2—manganese superoxide dismutase; SPP1—secreted phosphoprotein 1; SGO2—shugoshin-like 2; T2DM—Type 2 diabetes mellitus; TAC—total antioxidant capacity; TBARS—thiobarbituric acid reactive substances; TNF-α—tumor necrosis factor (TNF); TPO—thyroid peroxidase; TRX2—lysine-specific methyltransferase 2B (KMT2B); TTN—titin; TXNIP—thioredoxin-interacting protein; TXNRD2—thioredoxin reductase 2; UPDs—urinary polyphenol derivatives; VDR—vitamin D receptor; XRCC1—X-ray repair cross complementing 1; XRCC3—X-ray repair cross complementing 3. Gene symbols are written in italics.

**Table 2:** Overview of transcriptomic studies on oxidative stress and T2DM late complications (first search).

Biomarkers Studied	Participants and Design	Ethnicity/Region	Type of T2D Late Complications	Key Findings	Reference Year
Microarray expression analysis (26,803 unique gene sequences and 30,606 lncRNA), validation by qPCR	Total RNA from PB of 15 patients with T2DM, 15 with DKD, 15 with prediabetes, and 15 controls	Mexican	Microvascular (DKD)	300 genes higher expressed in DKD compared to other groups. qPCR confirmed higher *METLL22*, *PFKL*, *CCNB1*, and *CASP2* expression in DKD compared to other groups	[64] 2023
qPCR analysis of expression of *TP53*, *MMP9*, and *SLC23A2*	Total RNA from PBMCs in 49 patients with T2DM: 14 with DR, 35 without DR, and 32 controls	Spanish	Microvascular (DR)	Expression of *MMP9* was higher and *SLC23A2* was lower in T2DM compared to controls. *TP53*, *MMP9*, and *SLC23A2* expression differed between the T2DM-RD, T2DM+RD, and controls	[65] 2018
mRNA levels of endoplasmic reticulum stress, oxidative stress, and crosstalk markers	Total RNA from PBMCs in 30 patients with T2DM, 30 patients with DKD, and 30 healthy controls	Indian	Microvascular (DKD)	*ATF6*, *IRE1α*, *PERK*, *CHOP*, *GRP78*, *CYBA*, *TXNIP*, *PDI*, and *ERO1A* expression is higher in T2DM and DKD patients compared to other groups	[66] 2021
Serum *SOD*, *GPX,* and *CAT* gene expression and enzyme level	Total RNA from PB of 67 patients with T2DM: 22 with DR, and 45 without DR	Saudi Arabian	Microvascular (DR)	Poor glycemic control and altered *SOD*, *GPX*, and *CAT* expression associated with DR	[67] 2013
*NLRP3* mRNA expression—qPCR; Serum 8-OHdG, IL-1β, uHSP72 levels	Total RNA from PB of 45 T2DM patients: 15 normoalbuminuric, 15 microalbuminuric, 15 macroalbuminuric; and 15 healthy controls	Egyptian	Microvascular (albuminuria)	*NLRP3* mRNA expression, serum 8-OHdG, IL-1β, and uHSP72, and CHIT 1 activity increased in macroalbuminuric patients compared to other groups	[68] 2017
*RAGE* qPCR and WB, serum AGEs; PCO, AOP, and MDA	Total RNA from PBMCs of 75 T2DM patients: 25 without vascular complications, 25 with microvascular complications; 25 with macrovascular complications, and 25 healthy controls	Indian	Microvascular and macrovascular	*RAGE* mRNA expression and serum AGE levels are higher in T2DM patients with microvascular and macrovascular complications compared to T2DM without complications and controls	[69] 2014

Abbreviations: AGE—advanced glycosylation end products; AOPP—advanced oxidation protein products; ATF6—activating transcription factor 6; CAT—catalase; CASP2—caspase 2, apoptosis-related cysteine protease; CCNB1—cyclin B1; CHIT 1—chitinase 1; CHOP—C/EBP homologous protein (DNA damage-inducible transcript 3—DDIT3); CYBA—NAD(P)H oxidase p22phox (cytochrome b(-245), alpha subunit); DKD—diabetic kidney disease; DR—diabetic retinopathy; ELISA—enzyme-linked immunosorbent assay; ERO1A—ER oxidase 1α; GRP78—glucose-regulated protein-78; GPx—glutathione peroxidase; IL-1ß—interleukin 1-beta (IL1B); IRE1α—inositol-requiring enzyme 1α; LncRNA—long non-coding RNA; METTL22—methyltransferase 22, KIN27 lysine; MDA—malondialdehyde; MMP9—matrix metalloproteinase 9; NLRP3—NLR family, pyrin domain-containing 3; 8-OHdG—8-hydroxy-2′-deoxyguanosine; p22phox—cytochrome b(-245), alpha subunit (CYBA); PB—peripheral blood; PBMCs—peripheral blood mononuclear cells; PCO—protein carbonyl; PDI—protein disulfide isomerase; PERK—eukaryotic translation initiation factor 2-alpha kinase 3 (EIF2AK3); PFKL—phosphofructokinase, liver type; RAGE—advanced glycosylation end product-specific receptor (AGER); RNA—ribonucleic acid; SOD—superoxide dismutase; SLC23A2—solute carrier family 23 (nucleobase transporter), member 2; T2DM—type 2 diabetes mellitus; TP53—tumor protein p53; TXNIP—thioredoxin-interacting protein; uHSP72—urinary heat shock protein 72; WB—Western blot; qPCR—quantitative PCR. Gene symbols are written in italics.

**Table 3:** Characteristics of the studies researching antioxidants as an intervention in T2DM late complications (second search).

Type of T2D Late Complications	Antioxidant	Comparator	Duration	Participants and Design	Key Findings	The Overall Risk of Bias	Reference Year
Microvascular DKD	crocin tablets (15 mg/day)	control group (placebo pill)	90 days	44 Iranian patients (52% men) were randomly assigned to the crocin (22) or placebo (22) group, and 40 participants completed the trial	No statistically significant differences in terms of baseline anthropometric, clinical, and biochemical parameters.	low risk	[70] 2022
Microvascular DPN	200 mg Tocovid capsule (400 mg/day)	control group (placebo capsules; identical in size, shape, and color)	12 months, followed by a 6-month washout period	70 of 88 Malaysian patients completed this study treatment (33 vs. 37 in the placebo group); 72 participants were included in the washout analysis	Highly significant improvements in the CV of both median and sural sensory nerves. No significant differences from baseline between groups in nerve conduction parameters of all three nerves after 6 months of washout. No significant changes in TGFβ-1, or VEGF-A.	some concerns	[71] 2021
Microvascular DPN	two Pilates groups consumed 25 g dark (450 mg flavonoids per dose) or white chocolate 10 min before each Pilates training session (60 min), three times per week	control group consumed 25 g of the same dark chocolate at a pre-determined time 3 times per week, without any regular exercise	8 weeks (Pilates three times a week)	36 female Iranian patients (no other diabetic complications) in three groups: control (12); Pilates and dark chocolate (12); Pilates and flavanol-free white chocolate (12)	Significant increases in the TAC status in all groups. The Post hoc Bonferroni test showed a statistically significant increase in TAC for the dark chocolate group.	some concerns	[72] 2021
Macrovascular PVD (non-healing DFU)	PRP-FG dressing plus oral vitamin E (200 IU/2 Day) and C (250 IU/2Day), the dressing was changed 3 times a week	PRP-FG dressing plus oral placebo of vitamin E and C	8 weeks	25 Iranian patients with non-healing DFU, 13 in the intervention group, 12 in a placebo group	Significant wound size reduction and decrease in PAB, ESR, and hs-CRP were observed in the intervention group compared to the control group (*p* < 0.05).	low risk	[73] 2021
Microvascular DPN (mild-to-moderate)	decaffeinated GTE capsules 500 mg 30 min after meals three times daily	placebo material in a similar package	16 weeks (assessment made at baseline and after 4, 8, and 16 weeks of treatment)	194 Egyptian patients, 96 in GTE, and 98 in placebo group	After 8 weeks of treatment, patients in the GTE group expressed lower VAS scores, significantly lower TCSS scores and significantly lower VPT. The difference became more evident at 16 weeks.	some concerns	[74] 2021
Microvascular DKD	tocotrienol-rich vitamin E 200 mg (Tocovid SupraBioTM; Hovid Berhad, Ipoh, Malaysia) twice daily (400 mg/day)	identical-looking capsules (tocotrienol-free palm oil capsules) twice daily	12 months (2-month interval visits)	31 Malaysian patients in the intervention group and 28 in the control group	Reduced serum creatinine levels (*p* = 0.029) and significantly improved eGFR (*p* = 0.011) after eight months. Subgroup analysis (patients with stage 3 CKD) demonstrated persistent renoprotective effects over 12 months; Tocovid improved eGFR (*p* = 0.022) and serum creatinine (*p* = 0.042) but not UACR.	some concerns	[75] 2021
Macrovascular PVD (non-healing DFU)	nano curcumin supplements (80 mg/day)	tablet with placebo, similar in size and shape and odor	12 weeks	50 Iranian patients, 25 in curcumin and 25 in a placebo group	Significant improvement of TAC, GSH, total and LDL-cholesterol, and glycemic control but did not affect the ulcer size.	low risk	[76] 2021
Macrovascular CHD	curcumin (1000 mg/day)	placebo tablet (starch)	12 weeks	60 Iranian patients with T2DM and CHD, aged 45–85 years with 2- and 3-vessel CHD	Reduced MDA (*p* = 0.01), significantly increased TAC (*p* = 0.04) and GSH levels (*p* = 0.001); curcumin intake upregulated PPAR-γ and decreased PSQI (*p* = 0.01).	some concerns	[77] 2020
Microvascular DPN	tablet with a combination of four elements (SOD 10 mg, ALA 570 mg, B12 250 mcg, and Acetyl L-Carnitine 300 mg)	placebo (tablet)	12 months	85 Greek patients, 43 in the intervention group, 42 in the placebo group	BIO, MNSIQ, QL, pain, and SNCV, SNAP, and B12 levels had significantly improved in the intervention group, whereas in the placebo group, MCR and pain deteriorated. The changes in MNSIQ, QL, SNCV, BIO, and pain differed significantly between groups.	low risk	[78] 2020
Microvascular DPN	200 mg of Tocovid (tocotrienol-rich vitamin E) capsule twice a day	placebo capsule	8 weeks	80 Malaysian patients, 39 in the intervention group, 41 in a placebo group	Tocovid significantly improves the CV of all observed nerves (*p* < 0.001). Significantly higher levels of serum NGF.	low risk	[79] 2020
Microvascular DPN	GLA (320 mg/day)	ALA (600 mg/day)	12 weeks	Out of 100 Korean patients, 73 patients were included in the final analysis, 35 in the GLA group and 38 in the ALA group	Per-protocol analyses revealed significant decreases in the mean VAS and TSS scores compared to baseline in both groups, but there were no significant differences between the groups.	some concerns	[80] 2020
Microvascular NPDR	grape seed proanthocyanidin extract (GSPE) (150 mg/day in three tablets) or calcium dobesilate (CD; 750 mg/day)	placebo tablets, similar to GSPE tablets	12 months (follow-up visits at 3, 6, 9, and 12 months)	Korean patients with retinal thickening with HEs; 32 in the GSPE group, 35 in the CD group, and 19 in the placebo group were included in the analysis	Significantly greater improvement in HE severity in the GSPE group compared to placebo or CD group. In the GSPE group, TMV after 9 months of treatment was significantly decreased compared to baseline.	low risk	[81] 2019
Microvascular DPN	nanocurcumin (72% curcumin, 25% demethoxycurcumin, and bisdesmethoxycurcumin 3%) supplements (80 mg/day)	placebo capsules (polysorbate 80)	8 weeks	80 Iranian patients with non–insulin-dependent T2DM, 40 in the intervention group and 40 in the control group	Significant reduction in HbA1c (*p* < 0.001) and FBS (*p* = 0.004), total score of neuropathy (*p* < 0.001), total reflex score (*p* = 0.04), and temperature sensation (*p* = 0.01) compared to the placebo group.	some concerns	[82] 2019
Microvascular DKD	Resveratrol (500 mg/day)	carboxymethylcellulose as placebo; capsules were identical to the resveratrol ones	90 days	Out of 64 Iranian patients, 60 patients were analyzed, 30 in the intervention group and 30 in the placebo group.	The mean urine albumin/creatinine ratio was significantly reduced (*p* < 0.001), whereas eGFR (*p* = 0.08) and serum creatinine (*p* = 0.13) were unchanged. Serum antioxidant enzymes were significantly increased with resveratrol. After adjusting for confounding variables, the reduction in urinary albumin excretion was still significant.	some concern	[83] 2019
Microvascular DKD	vitamin E supplement (800 IU/day)	placebo, similar in shape and size capsule	12 weeks	27 Iranian patients in the intervention group and 27 in the placebo group	Significant elevation in vitamin E levels *p* < 0.001) and plasma glutathione levels. Significant reduction in serum total cholesterol (*p* = 0.03), LDL-cholesterol (*p* = 0.01), and ratio of total cholesterol to HDL-cholesterol ratio (*p* = 0.001), and HDL-cholesterol levels (*p* = 0.006). No favorable effect for TGA, very low-density lipoprotein cholesterol, NO, and TAC levels.	some concerns	[84] 2018
Macrovascular CHD	melatonin (10 mg/day)	placebo	12 weeks	Iranian patients with CHD, 30 in an intervention group, 30 in a placebo group	Significant increases in plasma GSH, NO, and HDL-cholesterol. Significant decreases in MDA, PCO, and serum hs-CRP levels, total-/HDL-cholesterol ratio, and systolic and diastolic blood pressure.	some concerns	[85] 2019
Macrovascular PVD (grade 3 DFU)	omega-3 fatty acids from flaxseed oil supplements (1000 mg/day)	placebo	12 weeks	60 Iranian patients (aged 40–85 years old) with grade 3 DFU; 30 in an intervention group and 30 in a placebo group.	Significant decreases in ulcer length (*p* = 0.03), width (*p* = 0.02), and depth (*p* = 0.01). Significant increase in plasma TAC (*p* < 0.001) and GSH concentrations (*p* = 0.03) compared to the placebo.	some concerns	[86] 2017
Microvascular DKD	200 mL/ day probiotic (Lactobacillus plantarum A7) fortified soy milk	soy milk in the control group	8 weeks	Out of 48 randomized Iranian patients with stage 1 or 2 nephropathy, 20 in the intervention group and 20 in the control group were included in the final analysis	Higher mean value of GSH, and increase in activity of GSH-Px and GR. Oxidized GSH concentration was significantly reduced in the probiotic soy milk group. However, no significant reduction of the serum 8-iso-prostaglandin F2a or MDA and no induction of TAC concentrations within and between the 2 groups.	some concerns	[87] 2017
Microvascular DKD	CUR (320 mg/day)	placebo supplement containing starch	8 weeks	51 Mexican patients with diabetic proteinuric CKD, 28 in an intervention group and 23 in a placebo group	No improvement in proteinuria, estimated GFR, or lipid profile. Enhanced antioxidant capacity in subjects with diabetic proteinuric CKD. No effect of CUR was observed on the antioxidant enzyme activities or Nrf2 activation.	some concerns	[88] 2016
Microvascular DKD	omega-3 fatty acid (1000 mg/day) from flaxseed oil	placebo	12 weeks	Out of 60 Iranian patients with DN, 30 were analyzed in the intervention group and 30 in the placebo group.	No significant effects on other lipid subfractions, biomarkers of inflammation, and oxidative stress compared with the placebo.	some concerns	[89] 2017
Microvascular DPN (symptomatic)	Two phases. First phase: ALA (600 mg tid); second phase: ALA (600 mg qd).	First phase: no comparator; Second phase: ALA withdrawal	First phase: 4 weeks; Second phase: 16 weeks	45 Mexican patients with polyneuropathy in the first phase and 16 patients in the intervention group and 17 patients in the control group in the second phase.	In patients who responded to the initial 4-week high-dose administration of ALA, subsequent treatment with ALA improved neuropathic symptoms, whereas ALA withdrawal was associated with a higher use of rescue analgesic drugs.	some concerns	[90] 2015
Microvascular DR (non-proliferative)	Group 1: Coenzyme Q10 (400 mg/day); Group 2: combined antioxidant therapy (composed of 10 mg of lutein, 4 mg of astaxanthin, 1 mg of zeaxanthin, 180 mg of vitamin C, 30 mg of vitamin E, 20 mg of zinc, and 1 mg of copper) once per day	Group 3: placebo; Group 4: without intervention.	6 months	60 Mexican patients in Group 1 (n = 20), Group 2 (n = 20), and Group 3 (n = 20), but healthy subjects of similar age and gender (blood donors) in Group 4 (n = 20).	LPO levels decreased significantly in groups exposed to antioxidant therapy (*p* < 0.0001). Levels of nitrites/nitrates decreased significantly (*p* < 0.0001) in the coenzyme Q10 group and CAT group. TAC increased significantly in groups subjected to pharmacological antioxidant therapy (*p* < 0.0001). The activity of CAT decreased significantly (*p* < 0.002) in groups exposed to antioxidant therapy. The activity of the GSH-px was decreased significantly with a tendency to normalize in groups subjected to antioxidant therapy (*p* < 0.0001).	high risk	[91] 2016
Macrovascular cerebrovascular complications (acute cerebral infarction)	ALA injection (600 mg in 250 mL 0.9% sodium chloride, iv. gtt once per day)	Vitamin C (3 g, solved in 250 mL 0.9% sodium chloride for iv. gtt injection, once per day)	3 weeks	90 Chinese patients, 46 patients in the intervention group and 44 in the control group.	After the treatment, the plasma SOD and GSH-Px levels increased, while MDA decreased (*p* < 0.05), compared to the control group (*p* < 0.01). NIHSS score, blood glucose, blood lipids, and HOMA-IA of the experiment group decreased significantly (*p* < 0.01).	high risk	[92] 2014
Microvascular DPN	Coenzyme Q10 (200 mg/day); as a 100 mg capsule taken twice daily	placebo	12 weeks	Out of 74 Iranian patients, 32 were analyzed in an intervention group and 30 in a placebo group.	CoQ10 did not improve the neuropathy signs compared to placebo, although it had some beneficial effects on TAC and hsCRP.	high risk	[93] 2014

Abbreviations: ALA—α-lipoic acid; ALT—alanine aminotransferase; AST—aspartate aminotransferase; BCVA—best-corrected visual acuity; BIO—vibration perception threshold with biothesiometer; BMI—body-mass index; BP—blood pressure; BUN—blood urea nitrogen; CARTs—cardiovascular autonomic reflex tests; CAT—catalase; CD—calcium dobesilate; CHD—coronary heart disease; CKD—chronic kidney disease; CPT—current perception threshold; CRP—C-reactive protein; CSMT—central subfield macular thickness; CUR—curcumin; DR—diabetic retinopathy, DKD—diabetic kidney disease; DFU—diabetic foot ulcer; DPN—diabetic peripheral neuropathy; DSPN—distal symmetric peripheral neuropathy; eGFR—estimated glomerular filtration rate; EQ 5D—EuroQol-5 dimensions; ESR—erythrocyte sedimentation rate; FA—fluorescein angiography; FBG—fasting blood glucose; FBS—fasting blood sugar; GGT—gamma-glutamyl transferase; GLA—γ-linolenic acid; GR—glutathione reductase; GSH—glutathione; GSH-Px—glutathione peroxidase; GSPE—grape seed proanthocya-nidin extract; GSSG—glutathione disulfide; GTE—green tea extract; HbA1c—glycated hemoglobin; HEs—hard exudates; HOMA-IA—Homeostatic Model Assessment for Insulin Resistance; hs-CRP—high sensitivity C-reactive protein; LDL—low-density lipoproteins; LPO—lipid peroxidation; mBPI-DPN—modified Brief Pain Inventory for DPN; MCR—mean circular resultant; MDA—malondialdehyde; MNSIE—Michigan Neuropathy Screening Instrument Examination; MNSIQ—Michigan Neuropathy Screening Instrument Questionnaire; MPO—myeloperoxidase; NA—not available; NCS—nerve conduction studies; NPDR—non-proliferative diabetic retinopathy; NGF—nerve growth factor; NIHSS—National Institutes of Health Stroke Scale; NO—nitric oxide; Nrf2—nuclear factor erythroid 2-related factor 2; PAB—prooxidant–antioxidant balance; PCO—protein carbonyl; PVD—peripheral vascular disease; PPAR-γ—peroxisome proliferator-activated receptor gamma; PRP-FG—platelet-rich plasma-fibrin glue; PSQI—Pittsburgh Sleep Quality Index; QL—quality of life; SCr—serum creatinine; SNAP—sural nerve conduction amplitude; SNCV—sural nerve conduction velocity; SOD—superoxide dismutase; T2DM—type 2 diabetes mellitus; TAC—total antioxidant capacity; TCSS—Toronto Clinical Scoring System; TGF-β—transforming growth factor beta; TMV—total macular volume; TNFR-1—tumor necrosis factor receptor 1; TSS—total symptom score; TXB2—thromboxane B2; uACR—urine albumin-creatinine ratio; VAS—visual analogue scale; VCAM1—vascular cell adhesion molecule 1; VEGF-A—vascular endothelial growth factor A; VPT—vibration perception threshold; 8-isoPGF2a—8-Iso-Prostaglandin F2α.

## Data Availability

All the used data are provided in the Supplementary Material.

## Supplementary Materials

The following supporting information can be downloaded at: https://www.mdpi.com/article/10.3390/antiox13030277/s1, Supplementary File S1: Methodology of Systematic Review; Supplementary File S2: Excluded Studies (first search); Supplementary File S3: Excluded Studies (second search).
