# Supplementary material for: Genetic and Transcriptomic Background of Oxidative Stress and Antioxidative Therapies in Late Complications of Type 2 Diabetes Mellitus: A Systematic Review
Source: Antioxidants (Basel). 2024 Feb 24;13(3):277. doi: 10.3390/antiox13030277 (PMC10967328; doi:10.3390/antiox13030277)
Supplement: Supplementary file 1 [file antioxidants-13-00277-s001.zip › Supplementary file 1_Methodology of Systematic Review.pdf]

Supplementary File S1: Methodology of the Systematic Review

S1 Genomic and transcriptomic basis of T2DM late complications (1st search)

S1.1. Eligibility Criteria

We have included clinical studies that focused on association of genetic variants and gene expression levels with T2DM late complications. As we tried to include a broad scope of the reports and since this field is not well researched, we have used a broad search strategy and did not limit the search to specific molecular genetic methods. After performing a broad search, we have filtered the retrieved studies with rigorous selection process. Studies that have not focused on associations with late T2DM complications but on other pathologies or development of the disease, or associations with oxidative stress biomarkers irrespective of T2DM complications were excluded. Moreover, studies on T1DM, T2DM without complications, or multimorbid patients were excluded from further analysis. Furthermore, review articles, systematic reviews, editorials, and reports that have not been written in the English language or could not be accessed in full text were also not included in the final analysis. Eligibility criteria (including subject characteristics, outcome, and study design criteria) for search 1 are summarized in Table S1. Genetic association studies and transcriptomic studies, included in the final review, were evaluated separately.

Table S1. Eligibility criteria regarding genetic and transcriptomic studies in type 2 diabetes mellitus late complications (1st search).

Eligibility criteria category	Inclusion criteria
Research question	Study focuses on genetic and transcriptomic factors associated with T2DM late complications.
Study subjects	Adults with T2DM that have developed T2DM late complications and respective controls.
Study design	Genetic association study or gene expression analysis
Outcome	Association with T2DM late complications
Year of publication	Last 10 years

S1.2. Data collection and extraction

Apart from publication metadata (authors, journal, year of publication), studies, obtained by the search strategy and selection process, were examined to extract details regarding study design (gene association or gene expression analysis), country of study, genes or transcripts studied, study participants characteristics (number of participants, inclusion and characteristics of control subjects), type of late T2DM complication assessed, key findings, and potential risk of bias.

S1.3. Data Synthesis Methodology

We have decided to group analysis of studies, included in the 1nd search, into genetic association studies and gene expression studies. Moreover, we have compiled a table presenting all the basic characteristics of the studies: genes or transcripts analyzed, study participants, country, type of T2DM late complications investigated, and key findings.

S2 Antioxidants as an intervention in type 2 diabetes mellitus late complications (2nd search)

S2.1. Eligibility Criteria

We have included studies that focused on randomized controlled trials using antioxidants as an intervention. Only the randomized controlled trials that focused on the role of antioxidants as an intervention in the population of patients with late T2DM complications were analyzed. As we tried to include a broad scope of the reports and since this field is not well researched, we have used a broad

search strategy and relied on filtering study reports with rigorous selection process. Studies that have not focused on late T2DM complications but on other pathologies or development of the disease, were excluded. Moreover, studies on T1DM, T2DM patients without complications, healthy population or multimorbid patients, were excluded from further analysis. Additionally, studies that have not used antioxidants as an intervention but used drugs, vitamins, or other substances with only possible indirect antioxidant mechanism were also excluded (similarly, the studies that have tested the efficiency of poor defined mix of antioxidants or possible antioxidants were ruled out). Furthermore, review articles, systematic reviews, editorials, and reports that have not be written in the English language or could not be accessed in full text were also not included in the final analysis. Eligibility criteria (including population, intervention, comparator, outcome, and study design criteria) for second search are summarized in Table S2. The studies, included in the final review, have been grouped by type of antioxidant used as an intervention.

Table S2. Eligibility criteria regarding antioxidants as an intervention in type 2 diabetes mellitus late complications (2nd search).

Eligibility criteria category	Inclusion criteria
Research question	Study focuses on determining the role of antioxidants as an intervention in the late complications of T2DM.
Population	Adults with T2DM that have developed late complications of diabetes.
Intervention	Administration of non-drug well-described substances with direct antioxidative effect.
Comparator	Eligible comparators included all of the following:<br>• treatment group(s) vs. control group(s),<br>• > 2 intervention groups (receiving different interventions, one of those antioxidants).
Outcome	Changes in the state of late complication, or changes of the antioxidant systems (i. e., total antioxidants capacity (TAC))
Study design	Peer-reviewed randomized controlled trials (RCT)

S2.2. Data collection and extraction

Apart from publication metadata (authors, journal, year of publication), studies, obtained by the search strategy and selection process, were examined to extract details regarding study design, country of study, intervention (type and dosage of antioxidants administered), comparator, study duration, study participants characteristics (number of participants, age of participants, and duration of T2DM), type of late T2DM complication assessed, predetermined outcome variables, key findings, and overall risk of bias.

S2.3. Data Synthesis Methodology

We have decided to group analysis of studies, included in the 2nd search, by the type of the antioxidant administered for the review. Moreover, we have compiled a table presenting all the basic characteristics of the studies (study design, country, intervention, comparator, duration, number of participants, their age and duration of diabetes, type of T2DM complication researched), their outcomes, key findings and corresponding determined overall risk of bias. In addition, we have also assessed the quality of the studies and included overall risk of bias in the summary tables.
