# Supplementary material for: Genetic and Transcriptomic Background of Oxidative Stress and Antioxidative Therapies in Late Complications of Type 2 Diabetes Mellitus: A Systematic Review
Source: Antioxidants (Basel). 2024 Feb 24;13(3):277. doi: 10.3390/antiox13030277 (PMC10967328; doi:10.3390/antiox13030277)
Supplement: Supplementary file 1 [file antioxidants-13-00277-s001.zip › Supplementary file 2_excluded studies (1st search).pdf]

**Supplementary file 2:** Studies excluded from the 1st search	**Exclusion criteria**
Duisenbek A, Lopez-Armas GC, Pérez M, Avilés Pérez MD, Aguilar Benitez JM, Pereira Pérez VR, Gorts Ortega J, Yessenbekova A, Ablaikhanova N, Escames G, Acuña-Castroviejo D, Rusanova I. Insights into the Role of Plasmatic and Exosomal microRNAs in Oxidative Stress-Related Metabolic Diseases. Antioxidants (Basel). 2023 Jun 16;12(6):1290. doi: 10.3390/antiox12061290. PMID: 37372020	Ineligible publication type
Zhang C, Li H, Wang S. Common gene signatures and molecular mechanisms of diabetic nephropathy and metabolic syndrome. Front Public Health. 2023 Mar 30;11:1150122. doi: 10.3389/fpubh.2023.1150122. eCollection 2023. PMID: 37143982	Study did not fulfil eligibility criteria
Gandhi GR, Hillary VE, Antony PJ, Zhong LLD, Yogesh D, Krishnakumar NM, Ceasar SA, Gan RY. A systematic review on anti-diabetic plant essential oil compounds: Dietary sources, effects, molecular mechanisms, and safety. Crit Rev Food Sci Nutr. 2023 Jan 28:1-20. doi: 10.1080/10408398.2023.2170320. Online ahead of print. PMID: 36708221	Ineligible publication type
Abdel Mageed SS, Doghish AS, Ismail A, El-Husseiny AA, Fawzi SF, Mahmoud AMA, El-Mahdy HA. The role of miRNAs in insulin resistance and diabetic macrovascular complications - A review. Int J Biol Macromol. 2023 Mar 1;230:123189. doi: 10.1016/j.ijbiomac.2023.123189. Epub 2023 Jan 7. PMID: 36623613	Ineligible publication type
Mirahmad M, Mohseni S, Tabatabaei-Malazy O, Esmaeili F, Alatab S, Bahramsoltani R, Ejtahed HS, Qulami H, Bitarafan Z, Arjmand B, Nazeri E. Antioxidative hypoglycemic herbal medicines with in vivo and in vitro activity against C-reactive protein; a systematic review. Phytomedicine. 2023 Jan;109:154615. doi: 10.1016/j.phymed.2022.154615. Epub 2022 Dec 18. PMID: 36610136	Ineligible publication type
Sahakyan G, Vejux A, Sahakyan N. The Role of Oxidative Stress-Mediated Inflammation in the Development of T2DM-Induced Diabetic Nephropathy: Possible Preventive Action of Tannins and Other Oligomeric Polyphenols. Molecules. 2022 Dec 18;27(24):9035. doi: 10.3390/molecules27249035. PMID: 36558167	Ineligible publication type
Zhang Y, Cao Y, Zheng R, Xiong Z, Zhu Z, Gao F, Man W, Duan Y, Lin J, Zhang X, Wu D, Jiang M, Zhang X, Li C, Gu X, Fan Y, Sun D. Fibroblast-specific activation of Rnd3 protects against cardiac remodeling in diabetic cardiomyopathy via suppression of Notch and TGF-β signaling. Theranostics. 2022 Oct 17;12(17):7250-7266. doi: 10.7150/thno.77043. eCollection 2022. PMID: 36438502	Study conducted on non-target group of patients (non-diabetic, diabetes type 1 etc.)
Sonthalia M, Roy BS, Chandrawanshi D, Ganesh GV, Jayasuriya R, Mohandas S, Rajagopal S, Ramkumar KM. Histone deacetylase inhibitors as antidiabetic agents: Advances and opportunities. Eur J Pharmacol. 2022 Nov 15;935:175328. doi: 10.1016/j.ejphar.2022.175328. Epub 2022 Oct 17. PMID: 36257383	Ineligible publication type
Marchelek-Mysliwiec M, Nalewajska M, Turoń-Skrzypińska A, Kotrych K, Dziedziejko V, Sulikowski T, Pawlik A. The Role of Forkhead Box O in Pathogenesis and Therapy of Diabetes Mellitus. Int J Mol Sci. 2022 Oct 1;23(19):11611. doi: 10.3390/ijms231911611. PMID: 36232910	Ineligible publication type
Waldman M, Singh SP, Shen HH, Alex R, Rezzani R, Favero G, Hochhauser E, Kornowski R, Arad M, Peterson SJ. Silencing the Adipocytokine NOV: A Novel Approach to Reversing Oxidative Stress-Induced Cardiometabolic Dysfunction. Cells. 2022 Sep 29;11(19):3060. doi: 10.3390/cells11193060. PMID: 36231029	Study conducted on non-target group of patients (non-diabetic, diabetes type 1 etc.)

Yu M, Sun Y, Shan X, Yang F, Chu G, Chen Q, Han L, Guo Z, Wang G. Therapeutic overexpression of miR-92a-2-5p ameliorated cardiomyocyte oxidative stress injury in the development of diabetic cardiomyopathy. Cell Mol Biol Lett. 2022 Oct 8;27(1):85. doi: 10.1186/s11658-022-00379-9. PMID: 36209049	Study conducted on non-target group of patients (non-diabetic, diabetes type 1 etc.)
Li H, Song D, Liu Q, Li L, Sun X, Guo J, Li D, Li P. miR-351 promotes atherosclerosis in diabetes by inhibiting the ITGB3/PIK3R1/Akt pathway and induces endothelial cell injury and lipid accumulation. Mol Med. 2022 Sep 30;28(1):120. doi: 10.1186/s10020-022-00547-9. PMID: 36180828	Study conducted on non-target group of patients (non-diabetic, diabetes type 1 etc.)
Stanigut AM, Pana C, Enciu M, Deacu M, Cimpineanu B, Tuta LA. Hypoxia-Inducible Factors and Diabetic Kidney Disease-How Deep Can We Go?. Int J Mol Sci. 2022 Sep 8;23(18):10413. doi: 10.3390/ijms231810413. PMID: 36142323	Ineligible publication type
Li H, Yang Q, Huang Z, Liang C, Zhang DH, Shi HT, Du JQ, Du BB, Zhang YZ. Dual-specificity phosphatase 12 attenuates oxidative stress injury and apoptosis in diabetic cardiomyopathy via the ASK1-JNK/p38 signaling pathway. Free Radic Biol Med. 2022 Nov 1;192:13-24. doi: 10.1016/j.freeradbiomed.2022.09.004. Epub 2022 Sep 13. PMID: 36108935	Study conducted on non-target group of patients (non-diabetic, diabetes type 1 etc.)
Wu Q, Li D, Huang C, Zhang G, Wang Z, Liu J, Yu H, Song B, Zhang N, Li B, Chu X. Glucose control independent mechanisms involved in the cardiovascular benefits of glucagon-like peptide-1 receptor agonists. Biomed Pharmacother. 2022 Sep;153:113517. doi: 10.1016/j.biopha.2022.113517. Epub 2022 Aug 10. PMID: 36076602	Ineligible publication type
Ntanyane Phasha MA, Soma P, Rooy MV, Phulukdaree A. MicroRNA 155, Factor XIII and Type 2 Diabetes Mellitus and Coronary Heart Disease. Curr Diabetes Rev. 2023;19(6):e190822207740. doi: 10.2174/1573399819999220819144402. PMID: 35993471	Ineligible publication type
Song J, Ni J, Yin X. The genetic side of diabetic kidney disease: a review. Int Urol Nephrol. 2023 Feb;55(2):335-343. doi: 10.1007/s11255-022-03319-w. Epub 2022 Aug 16. PMID: 35974289	Ineligible publication type
Klisic A, Radoman Vujacic I, Munjas J, Ninic A, Kotur-Stevuljevic J. Micro-ribonucleic acid modulation with oxidative stress and inflammation in patients with type 2 diabetes mellitus - a review article. Arch Med Sci. 2022 Apr 10;18(4):870-880. doi: 10.5114/aoms/146796. eCollection 2022. PMID: 35832702	Ineligible publication type
Rath P, Ranjan A, Chauhan A, Verma NK, Bhargava A, Prasad R, Jindal T. A Critical Review on Role of Available Synthetic Drugs and Phytochemicals in Insulin Resistance Treatment by Targeting PTP1B. Appl Biochem Biotechnol. 2022 Oct;194(10):4683-4701. doi: 10.1007/s12010-022-04028-x. Epub 2022 Jul 11. PMID: 35819691	Ineligible publication type
Shah MA, Haris M, Faheem HI, Hamid A, Yousaf R, Rasul A, Shah GM, Khalil AAK, Wahab A, Khan H, Alhasani RH, Althobaiti NA. Cross-Talk between Obesity and Diabetes: Introducing Polyphenols as an Effective Phytomedicine to Combat the Dual Sword Diabesity. Curr Pharm Des. 2022;28(19):1523-1542. doi: 10.2174/1381612828666220628123224. PMID: 35762558	Ineligible publication type
Garg SS, Gupta J. Polyol pathway and redox balance in diabetes. Pharmacol Res. 2022 Aug;182:106326. doi: 10.1016/j.phrs.2022.106326. Epub 2022 Jun 22. PMID: 35752357	Ineligible publication type
Ghosh C, Das N, Saha S, Kundu T, Sircar D, Roy P. Involvement of Cdkal1 in the etiology of type 2 diabetes mellitus and microvascular diabetic complications: a	Ineligible publication type

review. J Diabetes Metab Disord. 2022 Jan 13;21(1):991-1001. doi: 10.1007/s40200-021-00953-6. eCollection 2022 Jun. PMID: 35673487	
Bushra S, Al-Sadeq DW, Bari R, Sahara A, Fadel A, Rizk N. Adiponectin Ameliorates Hyperglycemia-Induced Retinal Endothelial Dysfunction, Highlighting Pathways, Regulators, and Networks. J Inflamm Res. 2022 May 27;15:3135-3166. doi: 10.2147/JIR.S358594. eCollection 2022. PMID: 35662872	Study conducted on non-target group of patients (non-diabetic, diabetes type 1 etc.)
Liu J, Sun M, Xia Y, Cui X, Jiang J. Phloretin ameliorates diabetic nephropathy by inhibiting nephrin and podocin reduction through a non-hypoglycemic effect. Food Funct. 2022 Jun 20;13(12):6613-6622. doi: 10.1039/d2fo00570k. PMID: 35622066	Study conducted on non-target group of patients (non-diabetic, diabetes type 1 etc.)
Kasinathan D, Matrougui K, Elango S, Belmandani S, Srinivas B, Muthusamy K, Narayanasamy Marimuthu P. Mitochondrial ATP6 and ND3 genes are associated with type 2 diabetic peripheral neuropathy. Diabetes Metab Syndr. 2022 Jun;16(6):102501. doi: 10.1016/j.dsx.2022.102501. Epub 2022 May 16. PMID: 35613490	Ineligible publication type
Wang J, Huang X, Liu H, Chen Y, Li P, Liu L, Li J, Ren Y, Huang J, Xiong E, Tian Z, Dai X. Empagliflozin Ameliorates Diabetic Cardiomyopathy via Attenuating Oxidative Stress and Improving Mitochondrial Function. Oxid Med Cell Longev. 2022 May 9;2022:1122494. doi: 10.1155/2022/1122494. eCollection 2022. PMID: 35585884	Study conducted on non-target group of patients (non-diabetic, diabetes type 1 etc.)
Kafeel S, Fawwad A, Basit A, Nawab SN. Clinical Association of Biochemical Variations Among Multilocus Genotypes of Antioxidant Enzymes with Susceptibility of Cataract in Hyperglycemia. Appl Biochem Biotechnol. 2022 Sep;194(9):3871-3889. doi: 10.1007/s12010-022-03957-x. Epub 2022 May 12. PMID: 35556207	Study conducted on non-target group of patients (non-diabetic, diabetes type 1 etc.)
Bhatti JS, Sehrawat A, Mishra J, Sidhu IS, Navik U, Khullar N, Kumar S, Bhatti GK, Reddy PH. Oxidative stress in the pathophysiology of type 2 diabetes and related complications: Current therapeutics strategies and future perspectives. Free Radic Biol Med. 2022 May 1;184:114-134. doi: 10.1016/j.freeradbiomed.2022.03.019. Epub 2022 Apr 7. PMID: 35398495	Ineligible publication type
Adeshara KA, Bangar N, Diwan AG, Tupe RS. Plasma glycation adducts and various RAGE isoforms are intricately associated with oxidative stress and inflammatory markers in type 2 diabetes patients with vascular complications. Diabetes Metab Syndr. 2022 Mar;16(3):102441. doi: 10.1016/j.dsx.2022.102441. Epub 2022 Feb 24. PMID: 35247657	Study did not fulfil eligibility criteria
Singh A, Kukreti R, Saso L, Kukreti S. Mechanistic Insight into Oxidative Stress-Triggered Signaling Pathways and Type 2 Diabetes. Molecules. 2022 Jan 30;27(3):950. doi: 10.3390/molecules27030950. PMID: 35164215	Ineligible publication type
Lima JEBF, Moreira NCS, Sakamoto-Hojo ET. Mechanisms underlying the pathophysiology of type 2 diabetes: From risk factors to oxidative stress, metabolic dysfunction, and hyperglycemia. Mutat Res Genet Toxicol Environ Mutagen. 2022 Feb-Mar;874-875:503437. doi: 10.1016/j.mrgentox.2021.503437. Epub 2021 Dec 14. PMID: 35151421	Ineligible publication type
Qiu D, Song S, Wang Y, Bian Y, Wu M, Wu H, Shi Y, Duan H. NAD(P)H: quinone oxidoreductase 1 attenuates oxidative stress and apoptosis by regulating Sirt1 in diabetic nephropathy. J Transl Med. 2022 Jan 28;20(1):44. doi: 10.1186/s12967-021-03197-3. PMID: 35090502	Study conducted on non-target group of patients (non-diabetic, diabetes type 1 etc.)

Kalai FZ, Boulaaba M, Ferdousi F, Isoda H. Effects of Isorhamnetin on Diabetes and Its Associated Complications: A Review of In Vitro and In Vivo Studies and a Post Hoc Transcriptome Analysis of Involved Molecular Pathways. Int J Mol Sci. 2022 Jan 9;23(2):704. doi: 10.3390/ijms23020704. PMID: 35054888	Ineligible publication type
Huang G, Li M, Tian X, Jin Q, Mao Y, Li Y. The Emerging Roles of IL-36, IL-37, and IL-38 in Diabetes Mellitus and its Complications. Endocr Metab Immune Disord Drug Targets. 2022;22(10):997-1008. doi: 10.2174/1871530322666220113142533. PMID: 35049442	Ineligible publication type
Patel R, Parmar N, Pramanik Palit S, Rathwa N, Ramachandran AV, Begum R. Diabetes mellitus and melatonin: Where are we?. Biochimie. 2022 Nov;202:2-14. doi: 10.1016/j.biochi.2022.01.001. Epub 2022 Jan 7. PMID: 35007648	Ineligible publication type
Xu Y, Tang G, Zhang C, Wang N, Feng Y. Gallic Acid and Diabetes Mellitus: Its Association with Oxidative Stress. Molecules. 2021 Nov 24;26(23):7115. doi: 10.3390/molecules26237115. PMID: 34885698	Ineligible publication type
Zhu D, Zhang X, Wang F, Ye Q, Yang C, Liu D. Irisin rescues diabetic cardiac microvascular injury via ERK1/2/Nrf2/HO-1 mediated inhibition of oxidative stress. Diabetes Res Clin Pract. 2022 Jan;183:109170. doi: 10.1016/j.diabres.2021.109170. Epub 2021 Dec 2. PMID: 34863716	Study conducted on non-target group of patients (non-diabetic, diabetes type 1 etc.)
Larsen EL, Kjær LK, Lundby-Christensen L, Boesgaard TW, Breum L, Gluud C, Hedetoft C, Krarup T, Lund SS, Mathiesen ER, Perrild H, Sneppen SB, Tarnow L, Thorsteinsson B, Vestergaard H, Poulsen HE, Madsbad S, Almdal TP. Effects of 18-months metformin versus placebo in combination with three insulin regimens on RNA and DNA oxidation in individuals with type 2 diabetes: A post-hoc analysis of a randomized clinical trial. Free Radic Biol Med. 2022 Jan;178:18-25. doi: 10.1016/j.freeradbiomed.2021.11.028. Epub 2021 Nov 22. PMID: 34823018	Study conducted on non-target group of patients (non-diabetic, diabetes type 1 etc.)
Ma T, Huang X, Zheng H, Huang G, Li W, Liu X, Liang J, Cao Y, Hu Y, Huang Y. SFRP2 Improves Mitochondrial Dynamics and Mitochondrial Biogenesis, Oxidative Stress, and Apoptosis in Diabetic Cardiomyopathy. Oxid Med Cell Longev. 2021 Nov 8;2021:9265016. doi: 10.1155/2021/9265016. eCollection 2021. PMID: 34790288	Study conducted on non-target group of patients (non-diabetic, diabetes type 1 etc.)
Zhou Z, Collado A, Sun C, Tratsiakovich Y, Mahdi A, Winter H, Chernogubova E, Seime T, Narayanan S, Jiao T, Jin H, Alvarsson M, Zheng X, Yang J, Hedin U, Catrina SB, Maegdefessel L, Pernow J. Downregulation of Erythrocyte miR-210 Induces Endothelial Dysfunction in Type 2 Diabetes. Diabetes. 2022 Feb 1;71(2):285-297. doi: 10.2337/db21-0093. PMID: 34753800	Study conducted on non-target group of patients (non-diabetic, diabetes type 1 etc.)
Yousri NA, Suhre K, Yassin E, Al-Shakaki A, Robay A, Elshafei M, Chidiac O, Hunt SC, Crystal RG, Fakhro KA. Metabolic and Metabo-Clinical Signatures of Type 2 Diabetes, Obesity, Retinopathy, and Dyslipidemia. Diabetes. 2022 Feb 1;71(2):184-205. doi: 10.2337/db21-0490. PMID: 34732537	Study did not fulfil eligibility criteria
He M, Long P, Chen T, Li K, Wei D, Zhang Y, Wang W, Hu Y, Ding Y, Wen A. ALDH2/SIRT1 Contributes to Type 1 and Type 2 Diabetes-Induced Retinopathy through Depressing Oxidative Stress. Oxid Med Cell Longev. 2021 Oct 23;2021:1641717. doi: 10.1155/2021/1641717. eCollection 2021. PMID: 34725563	Study conducted on non-target group of patients (non-diabetic, diabetes type 1 etc.)
Milluzzo A, Maugeri A, Barchitta M, Sciacca L, Agodi A. Epigenetic Mechanisms in Type 2 Diabetes Retinopathy: A Systematic Review. Int J Mol Sci. 2021 Sep 28;22(19):10502. doi: 10.3390/ijms221910502. PMID: 34638838	Ineligible publication type

Wong YH, Wong SH, Wong XT, Yap QY, Yip KY, Wong LZ, Chellappan DK, Bhattamisra SK, Candasamy M. Genetic associated complications of type 2 diabetes mellitus. Panminerva Med. 2022 Jun;64(2):274-288. doi: 10.23736/S0031-0808.21.04285-3. Epub 2021 Oct 5. PMID: 34609116	Ineligible publication type
Chen N, Song S, Yang Z, Wu M, Mu L, Zhou T, Shi Y. ChREBP deficiency alleviates apoptosis by inhibiting TXNIP/oxidative stress in diabetic nephropathy. J Diabetes Complications. 2021 Dec;35(12):108050. doi: 10.1016/j.jdiacomp.2021.108050. Epub 2021 Sep 23. PMID: 34600826	Study conducted on non-target group of patients (non-diabetic, diabetes type 1 etc.)
Sobha SP, Ebenezar K. Susceptibility of Glutathione-S-Transferase Polymorphism to CVD Develo- pment in Type 2 Diabetes Mellitus - A Review. Endocr Metab Immune Disord Drug Targets. 2022;22(2):225-234. doi: 10.2174/1871530321666210908115222. PMID: 34496736	Ineligible publication type
Nie P, Bai X, Lou Y, Zhu Y, Jiang S, Zhang L, Tian N, Luo P, Li B. Human umbilical cord mesenchymal stem cells reduce oxidative damage and apoptosis in diabetic nephropathy by activating Nrf2. Stem Cell Res Ther. 2021 Aug 11;12(1):450. doi: 10.1186/s13287-021-02447-x. PMID: 34380544	Study conducted on non-target group of patients (non-diabetic, diabetes type 1 etc.)
Das RR, Rahman MA, Al-Araby SQ, Islam MS, Rashid MM, Babteen NA, Alnajeebi AM, Alharbi HFH, Jeandet P, Rafi MKJ, Siddique TA, Uddin MN, Zakaria ZA. The Antioxidative Role of Natural Compounds from a Green Coconut Mesocarp Undeniably Contributes to Control Diabetic Complications as Evidenced by the Associated Genes and Biochemical Indexes. Oxid Med Cell Longev. 2021 Jul 27;2021:9711176. doi: 10.1155/2021/9711176. eCollection 2021. PMID: 34367469	Study did not fulfil eligibility criteria
Felisbino K, Granzotti JG, Bello-Santos L, Guiloski IC. Nutrigenomics in Regulating the Expression of Genes Related to Type 2 Diabetes Mellitus. Front Physiol. 2021 Jul 21;12:699220. doi: 10.3389/fphys.2021.699220. eCollection 2021. PMID: 34366888	Ineligible publication type
Wang H, Su X, Zhang QQ, Zhang YY, Chu ZY, Zhang JL, Ren Q. MicroRNA-93-5p participates in type 2 diabetic retinopathy through targeting Sirt1. Int Ophthalmol. 2021 Nov;41(11):3837-3848. doi: 10.1007/s10792-021-01953-4. Epub 2021 Jul 27. PMID: 34313929	Study conducted on non-target group of patients (non-diabetic, diabetes type 1 etc.)
Yao J, Li Y, Jin Y, Chen Y, Tian L, He W. Synergistic cardioptotection by tilianin and syringin in diabetic cardiomyopathy involves interaction of TLR4/NF-κB/NLRP3 and PGC1a/SIRT3 pathways. Int Immunopharmacol. 2021 Jul;96:107728. doi: 10.1016/j.intimp.2021.107728. Epub 2021 May 7. PMID: 33971494	Study conducted on non-target group of patients (non-diabetic, diabetes type 1 etc.)
Mahjabeen W, Khan DA, Mirza SA, Pervez MA. Effects of delta-tocotrienol supplementation on Glycemic Control, oxidative stress, inflammatory biomarkers and miRNA expression in type 2 diabetes mellitus: A randomized control trial. Phytother Res. 2021 Jul;35(7):3968-3976. doi: 10.1002/ptr.7113. Epub 2021 Apr 25. PMID: 33899292	Study did not fulfil eligibility criteria
Su X, Miao W, Li L, Zheng H, Hao G, Du L. Inhibition of Type-2 Diabetes Mellitus Development by Sophocarpine through Targeting PPARy-Regulated Gene Expression. Dokl Biochem Biophys. 2021 Mar;497(1):137-143. doi: 10.1134/S1607672921020150. Epub 2021 Apr 24. PMID: 33895930	Study conducted on non-target group of patients (non-diabetic, diabetes type 1 etc.)
Li Z, Deng X, Lan Y. Identification of a potentially functional circRNA-miRNA-mRNA regulatory network in type 2 diabetes mellitus by integrated microarray analysis. Minerva Endocrinol (Torino). 2021 Apr 1. doi: 10.23736/S2724-6507.21.03370-8. Online ahead of print. PMID: 33792237	Study conducted on non-target group of patients (non-diabetic, diabetes type 1 etc.)

Hammer SS, Vieira CP, McFarland D, Sandler M, Levitsky Y, Dorweiler TF, Lydic TA, Asare-Bediako B, Adu-Agyeiwaah Y, Sielski MS, Dupont M, Longhini AL, Li Calzi S, Chakraborty D, Seigel GM, Proshlyakov DA, Grant MB, Busik JV. Fasting and fasting-mimicking treatment activate SIRT1/LXRα and alleviate diabetes-induced systemic and microvascular dysfunction. Diabetologia. 2021 Jul;64(7):1674-1689. doi: 10.1007/s00125-021-05431-5. Epub 2021 Mar 26. PMID: 33770194	Study conducted on non-target group of patients (non-diabetic, diabetes type 1 etc.)
Jakubik D, Fitas A, Eyileten C, Jarosz-Popek J, Nowak A, Czajka P, Wicik Z, Sourij H, Siller-Matula JM, De Rosa S, Postula M. MicroRNAs and long non-coding RNAs in the pathophysiological processes of diabetic cardiomyopathy: emerging biomarkers and potential therapeutics. Cardiovasc Diabetol. 2021 Feb 27;20(1):55. doi: 10.1186/s12933-021-01245-2. PMID: 33639953	Ineligible publication type
Yao R, Cao Y, Wang C, Xu L, Zhang X, Deng Y, Li F, Wang S. Taohuajing reduces oxidative stress and inflammation in diabetic cardiomyopathy through the sirtuin 1/nucleotide-binding oligomerization domain-like receptor protein 3 pathway. BMC Complement Med Ther. 2021 Feb 26;21(1):78. doi: 10.1186/s12906-021-03218-0. PMID: 33637069	Study conducted on non-target group of patients (non-diabetic, diabetes type 1 etc.)
Zaibi N, Li P, Xu SZ. Protective effects of dapagliflozin against oxidative stress-induced cell injury in human proximal tubular cells. PLoS One. 2021 Feb 19;16(2):e0247234. doi: 10.1371/journal.pone.0247234. eCollection 2021. PMID: 33606763	Study conducted on non-target group of patients (non-diabetic, diabetes type 1 etc.)
Sávio-Silva C, Soinski-Sousa PE, Simplício-Filho A, Bastos RMC, Beyerstedt S, Rangel ÉB. Therapeutic Potential of Mesenchymal Stem Cells in a Pre-Clinical Model of Diabetic Kidney Disease and Obesity. Int J Mol Sci. 2021 Feb 4;22(4):1546. doi: 10.3390/ijms22041546. PMID: 33557007	Study conducted on non-target group of patients (non-diabetic, diabetes type 1 etc.)
Karamian M, Moossavi M, Hemmati M. From diabetes to renal aging: the therapeutic potential of adiponectin. J Physiol Biochem. 2021 May;77(2):205-214. doi: 10.1007/s13105-021-00790-4. Epub 2021 Feb 8. PMID: 33555532	Ineligible publication type
Ghoshal K, Chatterjee T, Chowdhury S, Sengupta S, Bhattacharyya M. Adiponectin Genetic Variant and Expression Coupled with Lipid Peroxidation Reveal New Signatures in Diabetic Dyslipidemia. Biochem Genet. 2021 Jun;59(3):781-798. doi: 10.1007/s10528-021-10030-5. Epub 2021 Feb 4. PMID: 33543406	Study conducted on non-target group of patients (non-diabetic, diabetes type 1 etc.)
Prasun P. Role of mitochondria in pathogenesis of type 2 diabetes mellitus. J Diabetes Metab Disord. 2020 Nov 2;19(2):2017-2022. doi: 10.1007/s40200-020-00679-x. eCollection 2020 Dec. PMID: 33520874	Ineligible publication type
Bokhary K, Aljaser F, Abudawood M, Tabassum H, Bakhsh A, Alhammad S, Aleyadhi R, Almajed F, Alsubki R. Role of Oxidative Stress and Severity of Diabetic Retinopathy in Type 1 and Type 2 Diabetes. Ophthalmic Res. 2021;64(4):613-621. doi: 10.1159/000514722. Epub 2021 Jan 26. PMID: 33498043	Study did not fulfil eligibility criteria
Natarajan R. Epigenetic Mechanisms in Diabetic Vascular Complications and Metabolic Memory: The 2020 Edwin Bierman Award Lecture. Diabetes. 2021 Feb;70(2):328-337. doi: 10.2337/dbi20-0030. PMID: 33472942	Ineligible publication type
Snegarova V, Naydenova D. Vitamin D: a Review of its Effects on Epigenetics and Gene Regulation. Folia Med (Plovdiv). 2020 Dec 31;62(4):662-667. doi: 10.3897/folmed.62.e50204.. PMID: 33415918	Ineligible publication type

Darmayanti S, Lesmana R, Meiliana A, Abdulah R. Genomics, Proteomics and Metabolomics Approaches for Predicting Diabetic Nephropathy in Type 2 Diabetes Mellitus Patients. Curr Diabetes Rev. 2021;17(6):e123120189796. doi: 10.2174/1573399817666210101105253. PMID: 33393899	Ineligible publication type
Behl T, Kaur I, Sehgal A, Sharma E, Kumar A, Grover M, Bungau S. Unfolding Nrf2 in diabetes mellitus. Mol Biol Rep. 2021 Jan;48(1):927-939. doi: 10.1007/s11033-020-06081-3. Epub 2021 Jan 3. PMID: 33389540	Ineligible publication type
Victor P, Umapathy D, George L, Juttada U, Ganesh GV, Amin KN, Viswanathan V, Ramkumar KM. Crosstalk between endoplasmic reticulum stress and oxidative stress in the progression of diabetic nephropathy. Cell Stress Chaperones. 2021 Mar;26(2):311-321. doi: 10.1007/s12192-020-01176-z. Epub 2020 Nov 7. PMID: 33161510	Study did not fulfil eligibility criteria
Man AWC, Xia N, Li H. Circadian Rhythm in Adipose Tissue: Novel Antioxidant Target for Metabolic and Cardiovascular Diseases. Antioxidants (Basel). 2020 Oct 9;9(10):968. doi: 10.3390/antiox9100968. PMID: 33050331	Ineligible publication type
Hashiesh HM, Meeran MFN, Sharma C, Sadek B, Kaabi JA, Ojha SK. Therapeutic Potential of β-Caryophyllene: A Dietary Cannabinoid in Diabetes and Associated Complications. Nutrients. 2020 Sep 28;12(10):2963. doi: 10.3390/nu12102963. PMID: 32998300	Ineligible publication type
Li Z, Guo H, Li J, Ma T, Zhou S, Zhang Z, Miao L, Cai L. Sulforaphane prevents type 2 diabetes-induced nephropathy via AMPK-mediated activation of lipid metabolic pathways and Nrf2 antioxidative function. Clin Sci (Lond). 2020 Sep 30;134(18):2469-2487. doi: 10.1042/CS20191088. PMID: 32940670	Study conducted on non-target group of patients (non-diabetic, diabetes type 1 etc.)
Pang M, Li Y, Gu W, Sun Z, Wang Z, Li L. Recent Advances in Epigenetics of Macrovascular Complications in Diabetes Mellitus. Heart Lung Circ. 2021 Feb;30(2):186-196. doi: 10.1016/j.hlc.2020.07.015. Epub 2020 Aug 30. PMID: 32873490	Ineligible publication type
Chatterjee T, De D, Chowdhury S, Bhattacharyya M. Nuclear factor NF-κB1 functional promoter polymorphism and its expression conferring the risk of Type 2 diabetes-associated dyslipidemia. Mamm Genome. 2020 Aug;31(7-8):252-262. doi: 10.1007/s00335-020-09846-0. Epub 2020 Aug 26. PMID: 32851488	Study conducted on non-target group of patients (non-diabetic, diabetes type 1 etc.)
Huang W, Man Y, Gao C, Zhou L, Gu J, Xu H, Wan Q, Long Y, Chai L, Xu Y, Xu Y. Short-Chain Fatty Acids Ameliorate Diabetic Nephropathy via GPR43-Mediated Inhibition of Oxidative Stress and NF-κB Signaling. Oxid Med Cell Longev. 2020 Aug 1;2020:4074832. doi: 10.1155/2020/4074832. eCollection 2020. PMID: 32831998	Study conducted on non-target group of patients (non-diabetic, diabetes type 1 etc.)
Hameed A, Galli M, Adamska-Patruno E, Krętowski A, Ciborowski M. Select Polyphenol-Rich Berry Consumption to Defer or Deter Diabetes and Diabetes-Related Complications. Nutrients. 2020 Aug 21;12(9):2538. doi: 10.3390/nu12092538. PMID: 32825710	Ineligible publication type
Hussain S, Khan AW, Akhmedov A, Suades R, Costantino S, Paneni F, Caidahl K, Mohammed SA, Hage C, Gkolfos C, Björck H, Pernow J, Lund LH, Lüscher TF, Cosentino F. Hyperglycemia Induces Myocardial Dysfunction via Epigenetic Regulation of JunD. Circ Res. 2020 Oct 23;127(10):1261-1273. doi: 10.1161/CIRCRESAHA.120.317132. Epub 2020 Aug 20. PMID: 32815777	Study conducted on non-target group of patients (non-diabetic, diabetes type 1 etc.)
Singh R, Chandel S, Dey D, Ghosh A, Roy S, Ravichandiran V, Ghosh D. Epigenetic modification and therapeutic targets of diabetes mellitus. Biosci Rep. 2020 Sep 30;40(9):BSR20202160. doi: 10.1042/BSR20202160. PMID: 32815547	Ineligible publication type

Rosta V, Trentini A, Passaro A, Zuliani G, Sanz JM, Bosi C, Bonaccorsi G, Bellini T, Cervellati C. Sex Difference Impacts on the Relationship between Paraoxonase-1 (PON1) and Type 2 Diabetes. Antioxidants (Basel). 2020 Jul 29;9(8):683. doi: 10.3390/antiox9080683. PMID: 32751395	Study conducted on non-target group of patients (non-diabetic, diabetes type 1 etc.)
Wingard MC, Frasier CR, Singh M, Singh K. Heart failure and diabetes: role of ATM. Curr Opin Pharmacol. 2020 Oct;54:27-35. doi: 10.1016/j.coph.2020.06.007. Epub 2020 Aug 1. PMID: 32745970	Ineligible publication type
Wang P, Wang D, Yang Y, Hou J, Wan J, Ran F, Dai X, Zhou P, Yang Y. Tom70 protects against diabetic cardiomyopathy through its antioxidant and antiapoptotic properties. Hypertens Res. 2020 Oct;43(10):1047-1056. doi: 10.1038/s41440-020-0518-x. Epub 2020 Jul 28. PMID: 32724135	Study conducted on non-target group of patients (non-diabetic, diabetes type 1 etc.)
Cai R, Jiang J. LncRNA ANRIL Silencing Alleviates High Glucose-Induced Inflammation, Oxidative Stress, and Apoptosis via Upregulation of MME in Podocytes. Inflammation. 2020 Dec;43(6):2147-2155. doi: 10.1007/s10753-020-01282-1. PMID: 32617859	Study conducted on non-target group of patients (non-diabetic, diabetes type 1 etc.)
Qiu F, Ma X, Shin YH, Chen J, Chen Q, Zhou K, Wu W, Liang W, Wu Y, Song Q, Ma JX. Pathogenic role of human C-reactive protein in diabetic retinopathy. Clin Sci (Lond). 2020 Jul 17;134(13):1613-1629. doi: 10.1042/CS20200085. PMID: 32602547	Study conducted on non-target group of patients (non-diabetic, diabetes type 1 etc.)
Packer M. Role of ketogenic starvation sensors in mediating the renal protective effects of SGLT2 inhibitors in type 2 diabetes. J Diabetes Complications. 2020 Sep;34(9):107647. doi: 10.1016/j.jdiacomp.2020.107647. Epub 2020 Jun 5. PMID: 32534886	Ineligible publication type
Gaonkar B, Prabhu K, Rao P, Kamat A, Rao Addoor K, Varma M. Plasma angiogenesis and oxidative stress markers in patients with diabetic retinopathy. Biomarkers. 2020 Jul;25(5):397-401. doi: 10.1080/1354750X.2020.1774654. Epub 2020 Jun 12. PMID: 32529845	Study did not fulfil eligibility criteria
Shao K, Xi L, Cang Z, Chen C, Huang S. Knockdown of NEAT1 exerts suppressive effects on diabetic retinopathy progression via inactivating TGF-β1 and VEGF signaling pathways. J Cell Physiol. 2020 Dec;235(12):9361-9369. doi: 10.1002/jcp.29740. Epub 2020 Apr 30. PMID: 32356340	Study conducted on non-target group of patients (non-diabetic, diabetes type 1 etc.)
Latini A, Borgiani P, De Benedittis G, D'Amato C, Greco C, Lauro D, Novelli G, Spallone V, Ciccacci C. Mitochondrial DNA Copy Number in Peripheral Blood Is Reduced in Type 2 Diabetes Patients with Polyneuropathy and Associated with a MIR499A Gene Polymorphism. DNA Cell Biol. 2020 Aug;39(8):1467-1472. doi: 10.1089/dna.2019.5326. Epub 2020 Apr 20. PMID: 32311290	Study did not fulfil eligibility criteria
Paramasivan S, Adav SS, Ngan SC, Dalan R, Leow MK, Ho HH, Sze SK. Serum albumin cysteine trioxidation is a potential oxidative stress biomarker of type 2 diabetes mellitus. Sci Rep. 2020 Apr 15;10(1):6475. doi: 10.1038/s41598-020-62341-z. PMID: 32296090	Study conducted on non-target group of patients (non-diabetic, diabetes type 1 etc.)
Rius-Pérez S, Torres-Cuevas I, Millán I, Ortega ÁL, Pérez S. PGC-1α, Inflammation, and Oxidative Stress: An Integrative View in Metabolism. Oxid Med Cell Longev. 2020 Mar 9;2020:1452696. doi: 10.1155/2020/1452696. eCollection 2020. PMID: 32215168	Ineligible publication type
Dworzański J, Strycharz-Dudziak M, Kliszczewska E, Kiełczykowska M, Dworzańska A, Drop B, Polz-Dacewicz M. Glutathione peroxidase (GPx) and superoxide	Study conducted on non-target group of patients

dismutase (SOD) activity in patients with diabetes mellitus type 2 infected with Epstein-Barr virus. PLoS One. 2020 Mar 25;15(3):e0230374. doi: 10.1371/journal.pone.0230374. eCollection 2020. PMID: 32210468	(non-diabetic, diabetes type 1 etc.)
Golpour P, Nourbakhsh M, Mazaherioun M, Janani L, Nourbakhsh M, Yaghmaei P. Improvement of NRF2 gene expression and antioxidant status in patients with type 2 diabetes mellitus after supplementation with omega-3 polyunsaturated fatty acids: A double-blind randomised placebo-controlled clinical trial. Diabetes Res Clin Pract. 2020 Apr;162:108120. doi: 10.1016/j.diabres.2020.108120. Epub 2020 Mar 16. PMID: 32194222	Study conducted on non-target group of patients (non-diabetic, diabetes type 1 etc.)
Xu J, Kitada M, Koya D. The impact of mitochondrial quality control by Sirtuins on the treatment of type 2 diabetes and diabetic kidney disease. Biochim Biophys Acta Mol Basis Dis. 2020 Jun 1;1866(6):165756. doi: 10.1016/j.bbadis.2020.165756. Epub 2020 Mar 5. PMID: 32147421	Ineligible publication type
Prakoso D, De Blasio MJ, Tate M, Kiriazis H, Donner DG, Qian H, Nash D, Deo M, Weeks KL, Parry LJ, Gregorevic P, McMullen JR, Ritchie RH. Gene therapy targeting cardiac phosphoinositide 3-kinase (p110α) attenuates cardiac remodeling in type 2 diabetes. Am J Physiol Heart Circ Physiol. 2020 Apr 1;318(4):H840-H852. doi: 10.1152/ajpheart.00632.2019. Epub 2020 Mar 6. PMID: 32142359	Study conducted on non-target group of patients (non-diabetic, diabetes type 1 etc.)
Luc K, Schramm-Luc A, Guzik TJ, Mikolajczyk TP. Oxidative stress and inflammatory markers in prediabetes and diabetes. J Physiol Pharmacol. 2019 Dec;70(6). doi: 10.26402/jpp.2019.6.01. Epub 2020 Feb 19. PMID: 32084643	Ineligible publication type
Baig S, Shabeer M, Parvaresh Rizi E, Agarwal M, Lee MH, Ooi DSQ, Chia C, Aung N, Ng G, Teo Y, Chhay V, Magkos F, Vidal-Puig A, Seet RCS, Toh SA. Heredity of type 2 diabetes confers increased susceptibility to oxidative stress and inflammation. BMJ Open Diabetes Res Care. 2020 Jan;8(1):e000945. doi: 10.1136/bmjdrc-2019-000945. PMID: 32049633	Study conducted on non-target group of patients (non-diabetic, diabetes type 1 etc.)
Khanam A, Ahmad S, Husain A, Rehman S, Farooqui A, Yusuf MA. Glycation and Antioxidants: Hand in the Glove of Antiglycation and Natural Antioxidants. Curr Protein Pept Sci. 2020;21(9):899-915. doi: 10.2174/1389203721666200210103304. PMID: 32039678	Ineligible publication type
Ravi R, Ragavachetty Nagaraj N, Subramaniam Rajesh B. Effect of advanced glycation end product on paraoxonase 2 expression: Its impact on endoplasmic reticulum stress and inflammation in HUVECs. Life Sci. 2020 Apr 1;246:117397. doi: 10.1016/j.lfs.2020.117397. Epub 2020 Feb 4. PMID: 32032646	Study conducted on non-target group of patients (non-diabetic, diabetes type 1 etc.)
Ramprasath T, Freddy AJ, Velmurugan G, Tomar D, Rekha B, Suvekbala V, Ramasamy S. Context-Dependent Regulation of Nrf2/ARE Axis on Vascular Cell Function during Hyperglycemic Condition. Curr Diabetes Rev. 2020;16(8):797-806. doi: 10.2174/1573399816666200130094512. PMID: 32000646	Ineligible publication type
Hussain T, Tan B, Murtaza G, Liu G, Rahu N, Saleem Kalhoro M, Hussain Kalhoro D, Adebowale TO, Usman Mazhar M, Rehman ZU, Martínez Y, Akber Khan S, Yin Y. Flavonoids and type 2 diabetes: Evidence of efficacy in clinical and animal studies and delivery strategies to enhance their therapeutic efficacy. Pharmacol Res. 2020 Feb;152:104629. doi: 10.1016/j.phrs.2020.104629. Epub 2020 Jan 7. PMID: 31918019	Ineligible publication type
Qadir MMF, Klein D, Álvarez-Cubela S, Domínguez-Bendala J, Pastori RL. The Role of MicroRNAs in Diabetes-Related Oxidative Stress. Int J Mol Sci. 2019 Oct 31;20(21):5423. doi: 10.3390/ijms20215423. PMID: 31683538	Ineligible publication type

Alouffi S, Khan MWA. Dicarbonyls Generation, Toxicities, Detoxifications and Potential Roles in Diabetes Complications. Curr Protein Pept Sci. 2020;21(9):890-898. doi: 10.2174/1389203720666191010155145. PMID: 31660813	Ineligible publication type
Gunawardena HP, Silva R, Sivakanesan R, Ranasinghe P, Katulanda P. Poor Glycaemic Control Is Associated with Increased Lipid Peroxidation and Glutathione Peroxidase Activity in Type 2 Diabetes Patients. Oxid Med Cell Longev. 2019 Aug 5;2019:9471697. doi: 10.1155/2019/9471697. eCollection 2019. PMID: 31467640	Study conducted on non-target group of patients (non-diabetic, diabetes type 1 etc.)
Lee JY, Lee YJ, Jeon HY, Han ET, Park WS, Hong SH, Kim YM, Ha KS. The vicious cycle between transglutaminase 2 and reactive oxygen species in hyperglycemic memory-induced endothelial dysfunction. FASEB J. 2019 Nov;33(11):12655-12667. doi: 10.1096/fj.201901358RR. Epub 2019 Aug 28. PMID: 31462079	Study conducted on non-target group of patients (non-diabetic, diabetes type 1 etc.)
Nazem MR, Asadi M, Jabbari N, Allameh A. Effects of zinc supplementation on superoxide dismutase activity and gene expression, and metabolic parameters in overweight type 2 diabetes patients: A randomized, double-blind, controlled trial. Clin Biochem. 2019 Jul;69:15-20. doi: 10.1016/j.clinbiochem.2019.05.008. Epub 2019 May 23. PMID: 31129183	Study conducted on non-target group of patients (non-diabetic, diabetes type 1 etc.)
Wimalawansa SJ. Vitamin D Deficiency: Effects on Oxidative Stress, Epigenetics, Gene Regulation, and Aging. Biology (Basel). 2019 May 11;8(2):30. doi: 10.3390/biology8020030. PMID: 31083546	Ineligible publication type
Baig S, Parvaresh Rizi E, Chia C, Shabeer M, Aung N, Loh TP, Magkos F, Vidal-Puig A, Seet RCS, Khoo CM, Toh SA. Genes Involved in Oxidative Stress Pathways Are Differentially Expressed in Circulating Mononuclear Cells Derived From Obese Insulin-Resistant and Lean Insulin-Sensitive Individuals Following a Single Mixed-Meal Challenge. Front Endocrinol (Lausanne). 2019 Apr 24;10:256. doi: 10.3389/fendo.2019.00256. eCollection 2019. PMID: 31068904	Study conducted on non-target group of patients (non-diabetic, diabetes type 1 etc.)
Wang XH, Yan CY, Liu JR. Hyperinsulinemia-induced KLF5 mediates endothelial angiogenic dysfunction in diabetic endothelial cells. J Mol Histol. 2019 Jun;50(3):239-251. doi: 10.1007/s10735-019-09821-3. Epub 2019 May 2. PMID: 31049798	Study conducted on non-target group of patients (non-diabetic, diabetes type 1 etc.)
Lei XW, Li Q, Zhang JZ, Zhang YM, Liu Y, Yang KH. The Protective Roles of Folic Acid in Preventing Diabetic Retinopathy Are Potentially Associated with Suppressions on Angiogenesis, Inflammation, and Oxidative Stress. Ophthalmic Res. 2019;62(2):80-92. doi: 10.1159/000499020. Epub 2019 Apr 24. PMID: 31018207	Study conducted on non-target group of patients (non-diabetic, diabetes type 1 etc.)
Herder C, Roden M, Ziegler D. Novel Insights into Sensorimotor and Cardiovascular Autonomic Neuropathy from Recent-Onset Diabetes and Population-Based Cohorts. Trends Endocrinol Metab. 2019 May;30(5):286-298. doi: 10.1016/j.tem.2019.02.007. Epub 2019 Mar 30. PMID: 30935671	Ineligible publication type
Sjöblad S. Could the high consumption of high glycaemic index carbohydrates and sugars, associated with the nutritional transition to the Western type of diet, be the common cause of the obesity epidemic and the worldwide increasing incidences of Type 1 and Type 2 diabetes?. Med Hypotheses. 2019 Apr;125:41-50. doi: 10.1016/j.mehy.2019.02.027. Epub 2019 Feb 10. PMID: 30902150	Ineligible publication type
Litvinova L, Zatolokin P, Vulf M, Mazunin I, Skuratovskaia D. The relationship between the mtDNA copy number in insulin-dependent tissues and markers of endothelial dysfunction and inflammation in obese patients. BMC Med Genomics. 2019 Mar 13;12(Suppl 2):41. doi: 10.1186/s12920-019-0486-7. PMID: 30871567	Study conducted on non-target group of patients (non-diabetic, diabetes type 1 etc.)

Ahmed SM, Johar D, Ali MM, El-Badri N. Insights into the Role of DNA Methylation and Protein Misfolding in Diabetes Mellitus. Endocr Metab Immune Disord Drug Targets. 2019;19(6):744-753. doi: 10.2174/1871530319666190305131813. PMID: 30834843	Ineligible publication type
Zhou P, Xie W, He S, Sun Y, Meng X, Sun G, Sun X. Ginsenoside Rb1 as an Anti-Diabetic Agent and Its Underlying Mechanism Analysis. Cells. 2019 Feb 28;8(3):204. doi: 10.3390/cells8030204. PMID: 30823412	Ineligible publication type
La Sala L, Mrakic-Sposta S, Tagliabue E, Prattichizzo F, Micheloni S, Sangalli E, Specchia C, Uccellatore AC, Lupini S, Spinetti G, de Candia P, Ceriello A. Circulating microRNA-21 is an early predictor of ROS-mediated damage in subjects with high risk of developing diabetes and in drug-naïve T2D. Cardiovasc Diabetol. 2019 Feb 25;18(1):18. doi: 10.1186/s12933-019-0824-2. PMID: 30803440	Study conducted on non-target group of patients (non-diabetic, diabetes type 1 etc.)
Spallone V. Update on the Impact, Diagnosis and Management of Cardiovascular Autonomic Neuropathy in Diabetes: What Is Defined, What Is New, and What Is Unmet. Diabetes Metab J. 2019 Feb;43(1):3-30. doi: 10.4093/dmj.2018.0259. PMID: 30793549	Ineligible publication type
Xourgia E, Papazafiropoulou A, Melidonis A. Circulating microRNAs as biomarkers for diabetic neuropathy: A novel approach. World J Exp Med. 2018 Nov 30;8(3):18-23. doi: 10.5493/wjem.v8.i3.18. eCollection 2018 Nov 30. PMID: 30596030	Ineligible publication type
Yaribeygi H, Atkin SL, Sahebkar A. Mitochondrial dysfunction in diabetes and the regulatory roles of antidiabetic agents on the mitochondrial function. J Cell Physiol. 2019 Jun;234(6):8402-8410. doi: 10.1002/jcp.27754. Epub 2018 Nov 11. PMID: 30417488	Ineligible publication type
Parim B, Sathibabu Uddandrao VV, Saravanan G. Diabetic cardiomyopathy: molecular mechanisms, detrimental effects of conventional treatment, and beneficial effects of natural therapy. Heart Fail Rev. 2019 Mar;24(2):279-299. doi: 10.1007/s10741-018-9749-1. PMID: 30349977	Ineligible publication type
Costantino S, Ambrosini S, Paneni F. The epigenetic landscape in the cardiovascular complications of diabetes. J Endocrinol Invest. 2019 May;42(5):505-511. doi: 10.1007/s40618-018-0956-3. Epub 2018 Oct 5. PMID: 30291588	Ineligible publication type
Kaur R, Kaur M, Singh J. Endothelial dysfunction and platelet hyperactivity in type 2 diabetes mellitus: molecular insights and therapeutic strategies. Cardiovasc Diabetol. 2018 Aug 31;17(1):121. doi: 10.1186/s12933-018-0763-3. PMID: 30170601	Ineligible publication type
Mafi A , Namazi G , Soleimani A , Bahmani F , Aghadavod E , Asemi Z . Metabolic and genetic response to probiotics supplementation in patients with diabetic nephropathy: a randomized, double-blind, placebo-controlled trial. Food Funct. 2018 Sep 19;9(9):4763-4770. doi: 10.1039/c8fo00888d. PMID: 30113051	Study conducted on non-target group of patients (non-diabetic, diabetes type 1 etc.)
Waldman M, Cohen K, Yadin D, Nudelman V, Gorfil D, Laniado-Schwartzman M, Kornwoski R, Aravot D, Abraham NG, Arad M, Hochhauser E. Regulation of diabetic cardiomyopathy by caloric restriction is mediated by intracellular signaling pathways involving 'SIRT1 and PGC-1α'. Cardiovasc Diabetol. 2018 Aug 2;17(1):111. doi: 10.1186/s12933-018-0754-4. PMID: 30071860	Study conducted on non-target group of patients (non-diabetic, diabetes type 1 etc.)
Li Z, Li Y, Overstreet JM, Chung S, Niu A, Fan X, Wang S, Wang Y, Zhang MZ, Harris RC. Inhibition of Epidermal Growth Factor Receptor Activation Is Associated With Improved Diabetic Nephropathy and Insulin Resistance in Type 2 Diabetes. Diabetes. 2018 Sep;67(9):1847-1857. doi: 10.2337/db17-1513. Epub 2018 Jun 29. PMID: 29959129	Study conducted on non-target group of patients (non-diabetic, diabetes type 1 etc.)

Gholami M, Zarei P, Sadeghi Sedeh B, Rafiei F, Khosrowbeygi A. Effects of coenzyme Q10 supplementation on serum values of adiponectin, leptin, 8-isoprostane and malondialdehyde in women with type 2 diabetes. Gynecol Endocrinol. 2018 Dec;34(12):1059-1063. doi: 10.1080/09513590.2018.1481944. Epub 2018 Jun 22. PMID: 29933718	Study conducted on non-target group of patients (non-diabetic, diabetes type 1 etc.)
Dalan R, Liuh Ling G. The protean role of haptoglobin and haptoglobin genotypes on vascular complications in diabetes mellitus. Eur J Prev Cardiol. 2018 Sep;25(14):1502-1519. doi: 10.1177/2047487318776829. Epub 2018 May 25. PMID: 29799294	Ineligible publication type
Nandi SS, Mishra PK. Targeting miRNA for Therapy of Juvenile and Adult Diabetic Cardiomyopathy. Adv Exp Med Biol. 2018;1056:47-59. doi: 10.1007/978-3-319-74470-4_4. PMID: 29754174	Ineligible publication type
Prabodha LBL, Sirisena ND, Dissanayake VHW. Susceptible and Prognostic Genetic Factors Associated with Diabetic Peripheral Neuropathy: A Comprehensive Literature Review. Int J Endocrinol. 2018 Mar 15;2018:8641942. doi: 10.1155/2018/8641942. eCollection 2018. PMID: 29736170	Ineligible publication type
Tang W, Chen X, Liu H, Lv Q, Zou J, Shi Y, Liu Z. Expression of Nrf2 Promotes Schwann Cell-Mediated Sciatic Nerve Recovery in Diabetic Peripheral Neuropathy. Cell Physiol Biochem. 2018;46(5):1879-1894. doi: 10.1159/000489373. Epub 2018 Apr 26. PMID: 29719281	Study conducted on non-target group of patients (non-diabetic, diabetes type 1 etc.)
Bene J, Hadzsiev K, Melegh B. Role of carnitine and its derivatives in the development and management of type 2 diabetes. Nutr Diabetes. 2018 Mar 7;8(1):8. doi: 10.1038/s41387-018-0017-1. PMID: 29549241	Ineligible publication type
Sarkar P, Bhowmick A, Banu S. Comparative analysis of different dietary antioxidants on oxidative stress pathway genes in L6 myotubes under oxidative stress. Cytotechnology. 2018 Aug;70(4):1177-1192. doi: 10.1007/s10616-018-0209-5. Epub 2018 Mar 14. PMID: 29541961	Study conducted on non-target group of patients (non-diabetic, diabetes type 1 etc.)
Petrie JR, Guzik TJ, Touyz RM. Diabetes, Hypertension, and Cardiovascular Disease: Clinical Insights and Vascular Mechanisms. Can J Cardiol. 2018 May;34(5):575-584. doi: 10.1016/j.cjca.2017.12.005. Epub 2017 Dec 11. PMID: 29459239	Ineligible publication type
Yang H, Feng A, Lin S, Yu L, Lin X, Yan X, Lu X, Zhang C. Fibroblast growth factor-21 prevents diabetic cardiomyopathy via AMPK-mediated antioxidation and lipid-lowering effects in the heart. Cell Death Dis. 2018 Feb 14;9(2):227. doi: 10.1038/s41419-018-0307-5. PMID: 29445083	Study conducted on non-target group of patients (non-diabetic, diabetes type 1 etc.)
Haybar H, Jalali MT, Zayeri ZD. What Genetics Tells us about Cardiovascular Disease in Diabetic Patients?. Cardiovasc Hematol Disord Drug Targets. 2018;18(2):147-152. doi: 10.2174/1871529X18666180212114305. PMID: 29437019	Article could not be accessed
Patel B, Mann GE, Chapple SJ. Concerted redox modulation by sulforaphane alleviates diabetes and cardiometabolic syndrome. Free Radic Biol Med. 2018 Jul;122:150-160. doi: 10.1016/j.freeradbiomed.2018.02.004. Epub 2018 Feb 7. PMID: 29427794	Ineligible publication type
Dandona P, Ghanim H, Abuaysheh S, Green K, Dhindsa S, Makdissi A, Batra M, Kuhadiya ND, Chaudhuri A. Exenatide Increases IL-1RA Concentration and Induces Nrf-2–Keap-1–Regulated Antioxidant Enzymes: Relevance to β-Cell Function. J Clin Endocrinol Metab. 2018 Mar 1;103(3):1180-1187. doi: 10.1210/jc.2017-02343. PMID: 29346597	Study conducted on non-target group of patients (non-diabetic, diabetes type 1 etc.)

Fadini GP, Albiero M, Bonora BM, Poncina N, Vigili de Kreutzenberg S, Avogaro A. p66Shc gene expression in peripheral blood mononuclear cells and progression of diabetic complications. Cardiovasc Diabetol. 2018 Jan 17;17(1):16. doi: 10.1186/s12933-018-0660-9. PMID: 29343271	Study did not fulfil eligibility criteria
Bai X, Geng J, Li X, Wan J, Liu J, Zhou Z, Liu X. Long Noncoding RNA LINC01619 Regulates MicroRNA-27a/Forkhead Box Protein O1 and Endoplasmic Reticulum Stress-Mediated Podocyte Injury in Diabetic Nephropathy. Antioxid Redox Signal. 2018 Aug 1;29(4):355-376. doi: 10.1089/ars.2017.7278. Epub 2018 Mar 12. PMID: 29334763	Study conducted on non-target group of patients (non-diabetic, diabetes type 1 etc.)
Zhou H, Wang S, Zhu P, Hu S, Chen Y, Ren J. Empagliflozin rescues diabetic myocardial microvascular injury via AMPK-mediated inhibition of mitochondrial fission. Redox Biol. 2018 May;15:335-346. doi: 10.1016/j.redox.2017.12.019. Epub 2017 Dec 30. PMID: 29306791	Drug effect study
Cai X, Li J, Wang M, She M, Tang Y, Li J, Li H, Hui H. GLP-1 Treatment Improves Diabetic Retinopathy by Alleviating Autophagy through GLP-1R-ERK1/2-HDAC6 Signaling Pathway. Int J Med Sci. 2017 Sep 19;14(12):1203-1212. doi: 10.7150/ijms.20962. eCollection 2017. PMID: 29104476	Study conducted on non-target group of patients (non-diabetic, diabetes type 1 etc.)
Deng X, Huang W, Peng J, Zhu TT, Sun XL, Zhou XY, Yang H, Xiong JF, He HQ, Xu YH, He YZ. Irisin Alleviates Advanced Glycation End Products-Induced Inflammation and Endothelial Dysfunction via Inhibiting ROS-NLRP3 Inflammasome Signaling. Inflammation. 2018 Feb;41(1):260-275. doi: 10.1007/s10753-017-0685-3. PMID: 29098483	Drug effect study
Wang XX, Wang D, Luo Y, Myakala K, Dobrinskikh E, Rosenberg AZ, Levi J, Kopp JB, Field A, Hill A, Lucia S, Qiu L, Jiang T, Peng Y, Orlicky D, Garcia G, Herman-Edelstein M, D'Agati V, Henriksen K, Adorini L, Pruzanski M, Xie C, Krausz KW, Gonzalez FJ, Ranjit S, Dvornikov A, Gratton E, Levi M. FXR/TGR5 Dual Agonist Prevents Progression of Nephropathy in Diabetes and Obesity. J Am Soc Nephrol. 2018 Jan;29(1):118-137. doi: 10.1681/ASN.2017020222. Epub 2017 Oct 31. PMID: 29089371	Study conducted on non-target group of patients (non-diabetic, diabetes type 1 etc.)
Kjær LK, Cejvanovic V, Henriksen T, Petersen KM, Hansen T, Pedersen O, Christensen CK, Torp-Pedersen C, Gerds TA, Brandslund I, Mandrup-Poulsen T, Poulsen HE. Cardiovascular and All-Cause Mortality Risk Associated With Urinary Excretion of 8-oxoGuo, a Biomarker for RNA Oxidation, in Patients With Type 2 Diabetes: A Prospective Cohort Study. Diabetes Care. 2017 Dec;40(12):1771-1778. doi: 10.2337/dc17-1150. Epub 2017 Oct 23. PMID: 29061564	Study did not fulfil eligibility criteria
Pinzón-Cortés JA, Perna-Chaux A, Rojas-Villamizar NS, Díaz-Basabe A, Polanía-Villanueva DC, Jácome MF, Mendivil CO, Groot H, López-Segura V. Effect of diabetes status and hyperglycemia on global DNA methylation and hydroxymethylation. Endocr Connect. 2017 Nov;6(8):708-725. doi: 10.1530/EC-17-0199. Epub 2017 Oct 9. PMID: 28993426	Study did not fulfil eligibility criteria
Hathaway QA, Pinti MV, Durr AJ, Waris S, Shepherd DL, Hollander JM. Regulating microRNA expression: at the heart of diabetes mellitus and the mitochondrion. Am J Physiol Heart Circ Physiol. 2018 Feb 1;314(2):H293-H310. doi: 10.1152/ajpheart.00520.2017. Epub 2017 Oct 6. PMID: 28986361	Ineligible publication type
Liu Y, Li H, Liu J, Han P, Li X, Bai H, Zhang C, Sun X, Teng Y, Zhang Y, Yuan X, Chu Y, Zhao B. Variations in MicroRNA-25 Expression Influence the Severity of Diabetic	Study conducted on non-target group of patients

Kidney Disease. J Am Soc Nephrol. 2017 Dec;28(12):3627-3638. doi: 10.1681/ASN.2015091017. Epub 2017 Sep 18. PMID: 28923913	(non-diabetic, diabetes type 1 etc.)
Saumya M, Subin EK, Suchithra TV. Network Analysis of MPO and Other Relevant Proteins Involved in Diabetic Foot Ulcer and Other Diabetic Complications. Interdiscip Sci. 2019 Jun;11(2):180-190. doi: 10.1007/s12539-017-0258-z. Epub 2017 Sep 13. PMID: 28905321	Study did not fulfil eligibility criteria
Yu SS, Du JL. Selenoprotein S: a therapeutic target for diabetes and macroangiopathy?. Cardiovasc Diabetol. 2017 Aug 10;16(1):101. doi: 10.1186/s12933-017-0585-8. PMID: 28797256	Ineligible publication type
Toma L, Sanda GM, Niculescu LS, Deleanu M, Stancu CS, Sima AV. Caffeic acid attenuates the inflammatory stress induced by glycated LDL in human endothelial cells by mechanisms involving inhibition of AGE-receptor, oxidative, and endoplasmic reticulum stress. Biofactors. 2017 Sep 10;43(5):685-697. doi: 10.1002/biof.1373. Epub 2017 Jul 28. PMID: 28753257	Study conducted on non-target group of patients (non-diabetic, diabetes type 1 etc.)
Costantino S, Paneni F, Battista R, Castello L, Capretti G, Chiandotto S, Tanese L, Russo G, Pitocco D, Lanza GA, Volpe M, Lüscher TF, Cosentino F. Impact of Glycemic Variability on Chromatin Remodeling, Oxidative Stress, and Endothelial Dysfunction in Patients With Type 2 Diabetes and With Target HbA(1c) Levels. Diabetes. 2017 Sep;66(9):2472-2482. doi: 10.2337/db17-0294. Epub 2017 Jun 20. PMID: 28634176	Study conducted on non-target group of patients (non-diabetic, diabetes type 1 etc.)
Schwartz SS, Epstein S, Corkey BE, Grant SFA, Gavin Iii JR, Aguilar RB, Herman ME. A Unified Pathophysiological Construct of Diabetes and its Complications. Trends Endocrinol Metab. 2017 Sep;28(9):645-655. doi: 10.1016/j.tem.2017.05.005. Epub 2017 Jun 16. PMID: 28629897	Ineligible publication type
Bitarafan F, Khodaeian M, Tabatabaei-Malazy O, Amoli MM. Influence of antioxidants' gene variants on risk of diabetes mellitus and its complications: a systematic review. Minerva Endocrinol. 2019 Sep;44(3):310-325. doi: 10.23736/S0391-1977.17.02632-3. Epub 2017 May 26. PMID: 28548478	Study did not fulfil eligibility criteria
Hochberg I, Berinstein EM, Milman U, Shapira C, Levy AP. Interaction Between the Haptoglobin Genotype and Vitamin E on Cardiovascular Disease in Diabetes. Curr Diab Rep. 2017 Jun;17(6):42. doi: 10.1007/s11892-017-0868-1. PMID: 28451949	Ineligible publication type
Rogulj D, El Aklouk I, Konjevoda P, Ljubić S, Pibernik Okanović M, Barbir A, Luburić M, Radman M, Budinski N, Vučić Lovrenčić M. Age-dependent systemic DNA damage in early Type 2 Diabetes mellitus. Acta Biochim Pol. 2017;64(2):233-238. doi: 10.18388/abp.2016_1313. Epub 2017 Mar 30. PMID: 28350403	Study conducted on non-target group of patients (non-diabetic, diabetes type 1 etc.)
Rojas-Carranza CA, Bustos-Cruz RH, Pino-Pinzon CJ, Ariza-Marquez YV, Gomez-Bello RM, Canadas-Garre M. Diabetes-Related Neurological Implications and Pharmacogenomics. Curr Pharm Des. 2018;24(15):1695-1710. doi: 10.2174/1381612823666170317165350. PMID: 28322157	Article could not be accessed
Rosique-Esteban N, Díaz-López A, Martínez-González MA, Corella D, Goday A, Martínez JA, Romaguera D, Vioque J, Arós F, Garcia-Rios A, Tinahones F, Estruch R, Fernández-García JC, Lapetra J, Serra-Majem L, Pinto X, Tur JA, Bueno-Cavanillas A, Vidal J, Delgado-Rodríguez M, Daimiel L, Vázquez C, Rubio MÁ, Ros E, Salas-Salvadó J; PREDIMED-PLUS investigators. Leisure-time physical activity, sedentary behaviors, sleep, and cardiometabolic risk factors at baseline in the PREDIMED-PLUS intervention trial: A cross-sectional analysis. PLoS One. 2017 Mar 8;12(3):e0172253. doi: 10.1371/journal.pone.0172253. eCollection 2017. PMID: 28273154	Study conducted on non-target group of patients (non-diabetic, diabetes type 1 etc.)

Li GY, Li ZB, Li F, Dong LP, Tang L, Xiang J, Li JM, Bao MH. Meta-Analysis on the Association of ALDH2 Polymorphisms and Type 2 Diabetic Mellitus, Diabetic Retinopathy. Int J Environ Res Public Health. 2017 Feb 8;14(2):165. doi: 10.3390/ijerph14020165. PMID: 28208752	Study did not fulfil eligibility criteria
Kamalden TA, Macgregor-Das AM, Kannan SM, Dunkerly-Eyring B, Khaliddin N, Xu Z, Fusco AP, Yazib SA, Chow RC, Duh EJ, Halushka MK, Steenbergen C, Das S. Exosomal MicroRNA-15a Transfer from the Pancreas Augments Diabetic Complications by Inducing Oxidative Stress. Antioxid Redox Signal. 2017 Nov 1;27(13):913-930. doi: 10.1089/ars.2016.6844. Epub 2017 Mar 17. PMID: 28173719	Study conducted on non-target group of patients (non-diabetic, diabetes type 1 etc.)
Kumar S, Kim YR, Vikram A, Naqvi A, Li Q, Kassan M, Kumar V, Bachschmid MM, Jacobs JS, Kumar A, Irani K. Sirtuin1-regulated lysine acetylation of p66Shc governs diabetes-induced vascular oxidative stress and endothelial dysfunction. Proc Natl Acad Sci U S A. 2017 Feb 14;114(7):1714-1719. doi: 10.1073/pnas.1614112114. Epub 2017 Jan 30. PMID: 28137876	Study conducted on non-target group of patients (non-diabetic, diabetes type 1 etc.)
Tsai FJ, Ho TJ, Cheng CF, Shiao YT, Chien WK, Chen JH, Liu X, Tsang H, Lin TH, Liao CC, Huang SM, Li JP, Lin CW, Lin JG, Lan YC, Liu YH, Hung CH, Lin JC, Lin CC, Lai CH, Liang WM, Lin YJ. Characteristics of Chinese herbal medicine usage in ischemic heart disease patients among type 2 diabetes and their protection against hydrogen peroxide-mediated apoptosis in H9C2 cardiomyoblasts. Oncotarget. 2017 Feb 28;8(9):15470-15489. doi: 10.18632/oncotarget.14657. PMID: 28099940	Study did not fulfil eligibility criteria
Peleli M, Carlstrom M. Adenosine signaling in diabetes mellitus and associated cardiovascular and renal complications. Mol Aspects Med. 2017 Jun;55:62-74. doi: 10.1016/j.mam.2016.12.001. Epub 2017 Jan 12. PMID: 28089906	Ineligible publication type
Rubin A, Salzberg AC, Imamura Y, Grivitishvilli A, Tombran-Tink J. Identification of novel targets of diabetic nephropathy and PEDF peptide treatment using RNA-seq. BMC Genomics. 2016 Nov 17;17(1):936. doi: 10.1186/s12864-016-3199-8. PMID: 27855634	Study conducted on non-target group of patients (non-diabetic, diabetes type 1 etc.)
Soroush N, Radfar M, Hamidi AK, Abdollahi M, Qorbani M, Razi F, Esfahani EN, Amoli MM. Vitamin D receptor gene FokI variant in diabetic foot ulcer and its relation with oxidative stress. Gene. 2017 Jan 30;599:87-91. doi: 10.1016/j.gene.2016.11.012. Epub 2016 Nov 9. PMID: 27836663	Study did not fulfil eligibility criteria
Dwinovan J, Colella AD, Chegeni N, Chataway TK, Sokoya EM. Proteomic analysis reveals downregulation of housekeeping proteins in the diabetic vascular proteome. Acta Diabetol. 2017 Feb;54(2):171-190. doi: 10.1007/s00592-016-0929-y. Epub 2016 Oct 28. PMID: 27796656	Study conducted on non-target group of patients (non-diabetic, diabetes type 1 etc.)
Shahzad K, Bock F, Al-Dabet MM, Gadi I, Nazir S, Wang H, Kohli S, Ranjan S, Mertens PR, Nawroth PP, Isermann B. Stabilization of endogenous Nrf2 by minocycline protects against Nlrp3-inflammasome induced diabetic nephropathy. Sci Rep. 2016 Oct 10;6:34228. doi: 10.1038/srep34228. PMID: 27721446	Study conducted on non-target group of patients (non-diabetic, diabetes type 1 etc.)
Qi X, Xu A, Gao Y, Shi Y, Sun X, Xu J, Liu J, Lan Q, Chang L, Zhang C, Yu H. Cardiac damage and dysfunction in diabetic cardiomyopathy are ameliorated by Grx1. Genet Mol Res. 2016 Sep 19;15(3). doi: 10.4238/gmr.15039000. PMID: 27706757	Study conducted on non-target group of patients (non-diabetic, diabetes type 1 etc.)
Zhou Y, Simmons D, Hambly BD, McLachlan CS. Interactions between UCP2 SNPs and telomere length exist in the absence of diabetes or pre-diabetes. Sci Rep. 2016 Sep 12;6:33147. doi: 10.1038/srep33147. PMID: 27615599	Study conducted on non-target group of patients

	(non-diabetic, diabetes type 1 etc.)
Palomino OM, Gouveia NM, Ramos S, Martín MA, Goya L. Protective Effect of Silybum marianum and Silibinin on Endothelial Cells Submitted to High Glucose Concentration. Planta Med. 2017 Jan;83(1-02):97-103. doi: 10.1055/s-0042-113135. Epub 2016 Aug 15. PMID: 27525510	Study conducted on non-target group of patients (non-diabetic, diabetes type 1 etc.)
Mergani A, Mansour AA, Askar T, Zahran RN, Mustafa AM, Mohammed MA, Saleh OM. Glutathione S-Transferase Pi-Ile 105 Val Polymorphism and Susceptibility to T2DM in Population from Turabah Region of Saudi Arabia. Biochem Genet. 2016 Aug;54(4):544-551. doi: 10.1007/s10528-016-9740-2. Epub 2016 Jul 1. PMID: 27368697	Study conducted on non-target group of patients (non-diabetic, diabetes type 1 etc.)
Muller CJF, Malherbe CJ, Chellan N, Yagasaki K, Miura Y, Joubert E. Potential of rooibos, its major C-glucosyl flavonoids, and Z-2-(β-D-glucopyranosyloxy)-3-phenylpropenoic acid in prevention of metabolic syndrome. Crit Rev Food Sci Nutr. 2018 Jan 22;58(2):227-246. doi: 10.1080/10408398.2016.1157568. Epub 2017 Jun 2. PMID: 27305453	Study conducted on non-target group of patients (non-diabetic, diabetes type 1 etc.)
Masi S, D'Aiuto F, Cooper J, Salpea K, Stephens JW, Hurel SJ, Deanfield JE, Humphries SE. Telomere length, antioxidant status and incidence of ischaemic heart disease in type 2 diabetes. Int J Cardiol. 2016 Aug 1;216:159-64. doi: 10.1016/j.ijcard.2016.04.130. Epub 2016 Apr 22. PMID: 27156058	Study did not fulfil eligibility criteria
Chen SY, Hsu YM, Lin YJ, Huang YC, Chen CJ, Lin WD, Liao WL, Chen YT, Lin WY, Liu YH, Yang JS, Sheu JC, Tsai FJ. Current concepts regarding developmental mechanisms in diabetic retinopathy in Taiwan. Biomedicine (Taipei). 2016 Jun;6(2):7. doi: 10.7603/s40681-016-0007-3. Epub 2016 May 5. PMID: 27154195	Ineligible publication type
Kisic B, Miric D, Dragojevic I, Rasic J, Popovic L. Role of Myeloperoxidase in Patients with Chronic Kidney Disease. Oxid Med Cell Longev. 2016;2016:1069743. doi: 10.1155/2016/1069743. Epub 2016 Apr 3. PMID: 27127544	Ineligible publication type
Wu H, Deng X, Shi Y, Su Y, Wei J, Duan H. PGC-1α, glucose metabolism and type 2 diabetes mellitus. J Endocrinol. 2016 Jun;229(3):R99-R115. doi: 10.1530/JOE-16-0021. Epub 2016 Apr 19. PMID: 27094040	Study conducted on non-target group of patients (non-diabetic, diabetes type 1 etc.)
Tibaut M, Petrovič D. Oxidative Stress Genes, Antioxidants and Coronary Artery Disease in Type 2 Diabetes Mellitus. Cardiovasc Hematol Agents Med Chem. 2016;14(1):23-38. doi: 10.2174/1871525714666160407143416. PMID: 27052028	Ineligible publication type
Fetterman JL, Holbrook M, Westbrook DG, Brown JA, Feeley KP, Bretón-Romero R, Linder EA, Berk BD, Weisbrod RM, Widlansky ME, Gokce N, Ballinger SW, Hamburg NM. Mitochondrial DNA damage and vascular function in patients with diabetes mellitus and atherosclerotic cardiovascular disease. Cardiovasc Diabetol. 2016 Mar 31;15:53. doi: 10.1186/s12933-016-0372-y. PMID: 27036979	Study did not fulfil eligibility criteria
Tamura Y, Takubo K, Aida J, Araki A, Ito H. Telomere attrition and diabetes mellitus. Geriatr Gerontol Int. 2016 Mar;16 Suppl 1:66-74. doi: 10.1111/ggi.12738. PMID: 27018285	Ineligible publication type
Zhou Y, Ning Z, Lee Y, Hambly BD, McLachlan CS. Shortened leukocyte telomere length in type 2 diabetes mellitus: genetic polymorphisms in mitochondrial uncoupling proteins and telomeric pathways. Clin Transl Med. 2016 Mar;5(1):8. doi: 10.1186/s40169-016-0089-2. Epub 2016 Mar 7. PMID: 26951191	Ineligible publication type

Breimer LH, Mikhailidis DP. Does bilirubin protect against developing diabetes mellitus?. J Diabetes Complications. 2016 May-Jun;30(4):728-37. doi: 10.1016/j.jdiacomp.2016.01.019. Epub 2016 Jan 26. PMID: 26922581	Ineligible publication type
Rani V, Deep G, Singh RK, Palle K, Yadav UC. Oxidative stress and metabolic disorders: Pathogenesis and therapeutic strategies. Life Sci. 2016 Mar 1;148:183-93. doi: 10.1016/j.lfs.2016.02.002. Epub 2016 Feb 3. PMID: 26851532	Ineligible publication type
Conserva F, Gesualdo L, Papale M. A Systems Biology Overview on Human Diabetic Nephropathy: From Genetic Susceptibility to Post-Transcriptional and Post-Translational Modifications. J Diabetes Res. 2016;2016:7934504. doi: 10.1155/2016/7934504. Epub 2015 Dec 20. PMID: 26798653	Ineligible publication type
Pradhan P, Upadhyay N, Tiwari A, Singh LP. Genetic and epigenetic modifications in the pathogenesis of diabetic retinopathy: a molecular link to regulate gene expression. New Front Ophthalmol. 2016;2(5):192-204. doi: 10.15761/NFO.1000145. Epub 2016 Oct 24. PMID: 28691104	Ineligible publication type
Kuo YR, Chien CM, Kuo MJ, Wang FS, Huang EY, Wang CJ. Endothelin-1 Expression Associated with Lipid Peroxidation and Nuclear Factor-κB Activation in Type 2 Diabetes Mellitus Patients with Angiopathy and Limb Amputation. Plast Reconstr Surg. 2016 Jan;137(1):187e-195e. doi: 10.1097/PRS.0000000000001886. PMID: 26710051	Study did not fulfil eligibility criteria
Long M, Rojo de la Vega M, Wen Q, Bharara M, Jiang T, Zhang R, Zhou S, Wong PK, Wondrak GT, Zheng H, Zhang DD. An Essential Role of NRF2 in Diabetic Wound Healing. Diabetes. 2016 Mar;65(3):780-93. doi: 10.2337/db15-0564. Epub 2015 Dec 30. PMID: 26718502	Study conducted on non-target group of patients (non-diabetic, diabetes type 1 etc.)
Liang X, Zhang T, Shi L, Kang C, Wan J, Zhou Y, Zhu J, Mi M. Ampelopsin protects endothelial cells from hyperglycemia-induced oxidative damage by inducing autophagy via the AMPK signaling pathway. Biofactors. 2015 Nov-Dec;41(6):463-75. doi: 10.1002/biof.1248. Epub 2015 Dec 8. PMID: 26644014	Study conducted on non-target group of patients (non-diabetic, diabetes type 1 etc.)
Yamagishi S, Nakamura N, Suematsu M, Kaseda K, Matsui T. Advanced Glycation End Products: A Molecular Target for Vascular Complications in Diabetes. Mol Med. 2015 Oct 27;21 Suppl 1(Suppl 1):S32-40. doi: 10.2119/molmed.2015.00067. PMID: 26605646	Ineligible publication type
Idewaki Y, Iwase M, Fujii H, Ohkuma T, Ide H, Kaizu S, Jodai T, Kikuchi Y, Hirano A, Nakamura U, Kubo M, Kitazono T. Association of Genetically Determined Aldehyde Dehydrogenase 2 Activity with Diabetic Complications in Relation to Alcohol Consumption in Japanese Patients with Type 2 Diabetes Mellitus: The Fukuoka Diabetes Registry. PLoS One. 2015 Nov 23;10(11):e0143288. doi: 10.1371/journal.pone.0143288. eCollection 2015. PMID: 26599441	Study conducted on non-target group of patients (non-diabetic, diabetes type 1 etc.)
Tatsch E, De Carvalho JA, Hausen BS, Bollick YS, Torbitz VD, Duarte T, Scolari R, Duarte MM, Londero SW, Vaucher RA, Premaor MO, Comim FV, Moresco RN. Oxidative DNA damage is associated with inflammatory response, insulin resistance and microvascular complications in type 2 diabetes. Mutat Res. 2015 Dec;782:17-22. doi: 10.1016/j.mrfmmm.2015.10.003. Epub 2015 Oct 20. PMID: 26520687	Study did not fulfil eligibility criteria
Bagul PK, Dinda AK, Banerjee SK. Effect of resveratrol on sirtuins expression and cardiac complications in diabetes. Biochem Biophys Res Commun. 2015 Dec 4-11;468(1-2):221-7. doi: 10.1016/j.bbrc.2015.10.126. Epub 2015 Oct 27. PMID: 26518647	Study conducted on non-target group of patients (non-diabetic, diabetes type 1 etc.)

Wu H, Kong L, Cheng Y, Zhang Z, Wang Y, Luo M, Tan Y, Chen X, Miao L, Cai L. Metallothionein plays a prominent role in the prevention of diabetic nephropathy by sulforaphane via up-regulation of Nrf2. Free Radic Biol Med. 2015 Dec;89:431-42. doi: 10.1016/j.freeradbiomed.2015.08.009. Epub 2015 Sep 28. PMID: 26415026	Study conducted on non-target group of patients (non-diabetic, diabetes type 1 etc.)
Nikooyeh B, Neyestani TR. Oxidative stress, type 2 diabetes and vitamin D: past, present and future. Diabetes Metab Res Rev. 2016 Mar;32(3):260-7. doi: 10.1002/dmrr.2718. Epub 2015 Nov 2. PMID: 26409185	Ineligible publication type
Abaidi H, Denden S, Ghazouani A, Trimèche A, Snoussi C, Haj Khelil A, Ben Chibani J, Hamdaoui MH. Mn-SOD 47 CC genotype in combination with high tea consumption may prevent complications in Tunisian type-2 diabetes. Genet Mol Res. 2015 Jul 31;14(3):8613-22. doi: 10.4238/2015.July.31.9. PMID: 26345792	Study did not fulfil eligibility criteria
Mohamed R, Jayakumar C, Chen F, Fulton D, Stepp D, Gansevoort RT, Ramesh G. Low-Dose IL-17 Therapy Prevents and Reverses Diabetic Nephropathy, Metabolic Syndrome, and Associated Organ Fibrosis. J Am Soc Nephrol. 2016 Mar;27(3):745-65. doi: 10.1681/ASN.2014111136. Epub 2015 Sep 2. PMID: 26334030	Study conducted on non-target group of patients (non-diabetic, diabetes type 1 etc.)
Zhu D, Wang H, Zhang J, Zhang X, Xin C, Zhang F, Lee Y, Zhang L, Lian K, Yan W, Ma X, Liu Y, Tao L. Irisin improves endothelial function in type 2 diabetes through reducing oxidative/nitrative stresses. J Mol Cell Cardiol. 2015 Oct;87:138-47. doi: 10.1016/j.yjmcc.2015.07.015. Epub 2015 Jul 28. PMID: 26225842	Study conducted on non-target group of patients (non-diabetic, diabetes type 1 etc.)
Lambers Heerspink HJ, Oberbauer R, Perco P, Heinzel A, Heinze G, Mayer G, Mayer B. Drugs meeting the molecular basis of diabetic kidney disease: bridging from molecular mechanism to personalized medicine. Nephrol Dial Transplant. 2015 Aug;30 Suppl 4:iv105-112. doi: 10.1093/ndt/gfv210. PMID: 26209732	Ineligible publication type
El-Refaei MF, Abduljawad SH, Alghamdi AH. Alternative Medicine in Diabetes - Role of Angiogenesis, Oxidative Stress, and Chronic Inflammation. Rev Diabet Stud. 2014 Fall-Winter;11(3-4):231-44. doi: 10.1900/RDS.2014.11.231. Epub 2015 Feb 10. PMID: 26177484	Ineligible publication type
Prattichizzo F, Giuliani A, Ceka A, Rippo MR, Bonfigli AR, Testa R, Procopio AD, Olivieri F. Epigenetic mechanisms of endothelial dysfunction in type 2 diabetes. Clin Epigenetics. 2015 May 23;7(1):56. doi: 10.1186/s13148-015-0090-4. eCollection 2015. PMID: 26015812	Ineligible publication type
Haldar SR, Chakrabarty A, Chowdhury S, Haldar A, Sengupta S, Bhattacharyya M. Oxidative stress-related genes in type 2 diabetes: association analysis and their clinical impact. Biochem Genet. 2015 Jun;53(4-6):93-119. doi: 10.1007/s10528-015-9675-z. Epub 2015 May 20. PMID: 25991559	Study conducted on non-target group of patients (non-diabetic, diabetes type 1 etc.)
Bagul PK, Banerjee SK. Application of resveratrol in diabetes: rationale, strategies and challenges. Curr Mol Med. 2015;15(4):312-30. doi: 10.2174/1566524015666150505155702. PMID: 25941821	Ineligible publication type
García-Fontana B, Morales-Santana S, Longobardo V, Reyes-García R, Rozas-Moreno P, García-Salcedo JA, Muñoz-Torres M. Relationship between Proinflammatory and Antioxidant Proteins with the Severity of Cardiovascular Disease in Type 2 Diabetes Mellitus. Int J Mol Sci. 2015 Apr 27;16(5):9469-83. doi: 10.3390/ijms16059469. PMID: 25923078	Study conducted on non-target group of patients (non-diabetic, diabetes type 1 etc.)

Tangvarasittichai S. Oxidative stress, insulin resistance, dyslipidemia and type 2 diabetes mellitus. World J Diabetes. 2015 Apr 15;6(3):456-80. doi: 10.4239/wjd.v6.i3.456. PMID: 25897356	Ineligible publication type
Prasad M, Bronson SC, Warrier T, Badarinath A, Rai S, Baid K, Sitaraman S, George A, Moses A, Saraswathy R, Vasuki R, Shanmugam A. Evaluation of DNA damage in Type 2 diabetes mellitus patients with and without peripheral neuropathy: A study in South Indian population. J Nat Sci Biol Med. 2015 Jan-Jun;6(1):80-4. doi: 10.4103/0976-9668.149096. PMID: 25810640	Study conducted on non-target group of patients (non-diabetic, diabetes type 1 etc.)
Abdali D, Samson SE, Grover AK. How effective are antioxidant supplements in obesity and diabetes?. Med Princ Pract. 2015;24(3):201-15. doi: 10.1159/000375305. Epub 2015 Mar 14. PMID: 25791371	Ineligible publication type
Liu ZZ, Zhao XZ, Zhang XS, Zhang M. Promoter DNA demethylation of Keap1 gene in diabetic cardiomyopathy. Int J Clin Exp Pathol. 2014 Dec 1;7(12):8756-62. eCollection 2014. PMID: 25674242	Study conducted on non-target group of patients (non-diabetic, diabetes type 1 etc.)
Paneni F, Costantino S, Battista R, Castello L, Capretti G, Chiandotto S, Scavone G, Villano A, Pitocco D, Lanza G, Volpe M, Lüscher TF, Cosentino F. Adverse epigenetic signatures by histone methyltransferase Set7 contribute to vascular dysfunction in patients with type 2 diabetes mellitus. Circ Cardiovasc Genet. 2015 Feb;8(1):150-8. doi: 10.1161/CIRCGENETICS.114.000671. Epub 2014 Dec 3. PMID: 25472959	Study did not fulfil eligibility criteria
Ergen A, Karagedik H, Karaali ZE, Isbir T. An association between MPO -463 G/A polymorphism and type 2 diabetes. Folia Biol (Praha). 2014;60(3):108-12. PMID: 25056433	Study conducted on non-target group of patients (non-diabetic, diabetes type 1 etc.)
Wang Y, Zhang Z, Sun W, Tan Y, Liu Y, Zheng Y, Liu Q, Cai L, Sun J. Sulforaphane attenuation of type 2 diabetes-induced aortic damage was associated with the upregulation of Nrf2 expression and function. Oxid Med Cell Longev. 2014;2014:123963. doi: 10.1155/2014/123963. Epub 2014 Feb 23. PMID: 24707343	Study conducted on non-target group of patients (non-diabetic, diabetes type 1 etc.)
Kohen Avramoglu R, Laplante MA, Le Quang K, Deshaies Y, Després JP, Larose E, Mathieu P, Poirier P, Pérusse L, Vohl MC, Sweeney G, Ylä-Herttuala S, Laakso M, Uusitupa M, Marette A. The genetic and metabolic determinants of cardiovascular complications in type 2 diabetes: recent insights from animal models and clinical investigations. Can J Diabetes. 2013 Oct;37(5):351-8. doi: 10.1016/j.jcjd.2013.08.262. PMID: 24500564	Ineligible publication type
Zeadin MG, Petlura CI, Werstuck GH. Molecular mechanisms linking diabetes to the accelerated development of atherosclerosis. Can J Diabetes. 2013 Oct;37(5):345-50. doi: 10.1016/j.jcjd.2013.06.001. PMID: 24500563	Ineligible publication type
Andrews Guzmán M, Arredondo Olguín M, Olivares Gronhert M. Glycemic control and oxidative stress markers and their relationship with the thioredoxin interacting protein (TXNIP) gene in type 2 diabetic patients. Nutr Hosp. 2014 Sep 12;31(3):1129-33. doi: 10.3305/nh.2015.31.3.7955. PMID: 25726203	Study conducted on non-target group of patients (non-diabetic, diabetes type 1 etc.)
Kamiyama M, Urushihara M, Morikawa T, Konishi Y, Imanishi M, Nishiyama A, Kobori H. Oxidative stress/angiotensinogen/renin-angiotensin system axis in patients with diabetic nephropathy. Int J Mol Sci. 2013 Nov 21;14(11):23045-62. doi: 10.3390/ijms141123045. PMID: 24284398	Study did not fulfil eligibility criteria

Okada-Iwabu M, Yamauchi T, Iwabu M, Honma T, Hamagami K, Matsuda K, Yamaguchi M, Tanabe H, Kimura-Someya T, Shirouzu M, Ogata H, Tokuyama K, Ueki K, Nagano T, Tanaka A, Yokoyama S, Kadowaki T. A small-molecule AdipoR agonist for type 2 diabetes and short life in obesity. Nature. 2013 Nov 28;503(7477):493-9. doi: 10.1038/nature12656. Epub 2013 Oct 30. PMID: 24172895	Study conducted on non-target group of patients (non-diabetic, diabetes type 1 etc.)
Pinheiro DS, Rocha Filho CR, Mundim CA, Júnior Pde M, Ulhoa CJ, Reis AA, Ghedini PC. Evaluation of glutathione S-transferase GSTM1 and GSTT1 deletion polymorphisms on type-2 diabetes mellitus risk. PLoS One. 2013 Oct 3;8(10):e76262. doi: 10.1371/journal.pone.0076262. eCollection 2013. PMID: 24098457	Study conducted on non-target group of patients (non-diabetic, diabetes type 1 etc.)
Paneni F, Costantino S, Volpe M, Lüscher TF, Cosentino F. Epigenetic signatures and vascular risk in type 2 diabetes: a clinical perspective. Atherosclerosis. 2013 Oct;230(2):191-7. doi: 10.1016/j.atherosclerosis.2013.07.003. Epub 2013 Jul 16. PMID: 24075743	Ineligible publication type
Binici DN, Karaman A, Coşkun M, Oğlu AU, Uçar F. Genomic damage in patients with type-2 diabetes mellitus. Genet Couns. 2013;24(2):149-56. PMID: 24032284	Study conducted on non-target group of patients (non-diabetic, diabetes type 1 etc.)
Gupta S, Gambhir JK, Kalra O, Gautam A, Shukla K, Mehndiratta M, Agarwal S, Shukla R. Association of biomarkers of inflammation and oxidative stress with the risk of chronic kidney disease in Type 2 diabetes mellitus in North Indian population. J Diabetes Complications. 2013 Nov-Dec;27(6):548-52. doi: 10.1016/j.jdiacomp.2013.07.005. Epub 2013 Sep 6. PMID: 24012111	Study did not fulfil eligibility criteria
Roberts AC, Porter KE. Cellular and molecular mechanisms of endothelial dysfunction in diabetes. Diab Vasc Dis Res. 2013 Nov;10(6):472-82. doi: 10.1177/1479164113500680. Epub 2013 Sep 3. PMID: 24002671	Ineligible publication type
Ramprasath T, Selvam GS. Potential impact of genetic variants in Nrf2 regulated antioxidant genes and risk prediction of diabetes and associated cardiac complications. Curr Med Chem. 2013;20(37):4680-93. doi: 10.2174/09298673113209990154. PMID: 23834171	Ineligible publication type
Kato M, Castro NE, Natarajan R. MicroRNAs: potential mediators and biomarkers of diabetic complications. Free Radic Biol Med. 2013 Sep;64:85-94. doi: 10.1016/j.freeradbiomed.2013.06.009. Epub 2013 Jun 12. PMID: 23770198	Ineligible publication type
Velmurugan GV, Sundaresan NR, Gupta MP, White C. Defective Nrf2-dependent redox signalling contributes to microvascular dysfunction in type 2 diabetes. Cardiovasc Res. 2013 Oct 1;100(1):143-50. doi: 10.1093/cvr/cvt125. Epub 2013 May 27. PMID: 23715558	Study conducted on non-target group of patients (non-diabetic, diabetes type 1 etc.)
Kotani K, Tsuzaki K, Taniguchi N, Sakane N. Correlation between reactive oxygen metabolites & atherosclerotic risk factors in patients with type 2 diabetes mellitus. Indian J Med Res. 2013 Apr;137(4):742-8. PMID: 23703342	Study conducted on non-target group of patients (non-diabetic, diabetes type 1 etc.)
Bahadoran Z, Mirmiran P, Azizi F. Potential efficacy of broccoli sprouts as a unique supplement for management of type 2 diabetes and its complications. J Med Food. 2013 May;16(5):375-82. doi: 10.1089/jmf.2012.2559. Epub 2013 Apr 30. PMID: 23631497	Ineligible publication type

Ruiz S, Pergola PE, Zager RA, Vaziri ND. Targeting the transcription factor Nrf2 to ameliorate oxidative stress and inflammation in chronic kidney disease. Kidney Int. 2013 Jun;83(6):1029-41. doi: 10.1038/ki.2012.439. Epub 2013 Jan 16. PMID: 23325084	Ineligible publication type
Forbes JM, Cooper ME. Mechanisms of diabetic complications. Physiol Rev. 2013 Jan;93(1):137-88. doi: 10.1152/physrev.00045.2011. PMID: 23303908	Ineligible publication type
Mandavia CH, Aroor AR, Demarco VG, Sowers JR. Molecular and metabolic mechanisms of cardiac dysfunction in diabetes. Life Sci. 2013 Mar 28;92(11):601-8. doi: 10.1016/j.lfs.2012.10.028. Epub 2012 Nov 9. PMID: 23147391	Ineligible publication type
Osmenda G, Matusik PT, Sliwa T, Czesnikiewicz-Guzik M, Skupien J, Malecki MT, Siedlinski M. Nicotinamide adenine dinucleotide phosphate (NADPH) oxidase p22phox subunit polymorphisms, systemic oxidative stress, endothelial dysfunction, and atherosclerosis in type 2 diabetes mellitus. Pol Arch Intern Med. 2021 May 25;131(5):447-454. doi: 10.20452/pamw.15937. PMID: 33851807.	Study did not fulfil eligibility criteria
Gusti AMT, Qusti SY, Bahijri SM, Toraih EA, Bokhari S, Attallah SM, Alzahrani A, Alshehri WMA, Alotaibi H, Fawzy MS. Glutathione S-Transferase (GSTT1 rs17856199) and Nitric Oxide Synthase (NOS2 rs2297518) Genotype Combination as Potential Oxidative Stress-Related Molecular Markers for Type 2 Diabetes Mellitus. Diabetes Metab Syndr Obes. 2021 Mar 25;14:1385-1403. doi: 10.2147/DMSO.S300525. PMID: 33790606.	Study did not fulfil eligibility criteria
Etemad A, Vasudevan R, Aziz AF, Yusof AK, Khazaei S, Fawzi N, Jamalpour S, Arkani M, Mohammad NA, Ismail P. Analysis of selected glutathione S-transferase gene polymorphisms in Malaysian type 2 diabetes mellitus patients with and without cardiovascular disease. Genet Mol Res. 2016 Apr 7;15(2). doi: 10.4238/gmr.15025845. PMID: 27173202.	Study did not fulfil eligibility criteria
Chawla D, Bansal S, Banerjee BD, Madhu SV, Kalra OP, Tripathi AK. Role of advanced glycation end product (AGE)-induced receptor (RAGE) expression in diabetic vascular complications. Microvasc Res. 2014 Sep;95:1-6. doi: 10.1016/j.mvr.2014.06.010. Epub 2014 Jun 28. PMID: 24984291.	Study did not fulfil eligibility criteria
Mokhtary N, Mousavi SN, Sotoudeh G, Qorbani M, Dehghani M, Koohdani F. Deletion allele of Apo B gene is associated with higher inflammation, oxidative stress and dyslipidemia in obese type 2 diabetic patients: an analytical cross-sectional study. BMC Endocr Disord. 2022 Mar 22;22(1):73. doi: 10.1186/s12902-022-00991-y. PMID: 35317787;	Study did not fulfil eligibility criteria
Jafari Azad B, Yaseri M, Daneshzad E, Koohdani F. Interaction between Apo A-II -265T > C polymorphism and dietary total antioxidant capacity on some oxidative stress and inflammatory markers in patients with type 2 diabetes mellitus. Br J Nutr. 2022 Jul 14;128(1):13-29. doi: 10.1017/S0007114521002993. Epub 2021 Aug 10. PMID: 34372957.	Study did not fulfil eligibility criteria
Koohdani F, Sadrzadeh-Yeganeh H, Djalali M, Eshraghian M, Keramat L, Mansournia MA, Zamani E. Association between ApoA-II -265T/C polymorphism and oxidative stress in patients with type 2 diabetes mellitus. J Diabetes Complications. 2015 Sep-Oct;29(7):908-12. doi: 10.1016/j.jdiacomp.2015.05.024. Epub 2015 Jun 10. PMID: 26104730.	Study did not fulfil eligibility criteria
