# Supplementary material for: Genetic and Transcriptomic Background of Oxidative Stress and Antioxidative Therapies in Late Complications of Type 2 Diabetes Mellitus: A Systematic Review
Source: Antioxidants (Basel). 2024 Feb 24;13(3):277. doi: 10.3390/antiox13030277 (PMC10967328; doi:10.3390/antiox13030277)
Supplement: Supplementary file 1 [file antioxidants-13-00277-s001.zip › Supplementary file 3_excluded studies (2nd search).pdf]

**Supplementary file 3:** Studies excluded from the 2nd search	**Exclusion criteria**
Xia J, Yu J, Xu H, Zhou Y, Li H, Yin S, Xu D, Wang Y, Xia H, Liao W, Wang S, Sun G. 2023. Comparative effects of vitamin and mineral supplements in the management of type 2 diabetes in primary care: A systematic review and network meta-analysis of randomized controlled trials. PMID: 36638933. doi:10.1016/j.phrs.2023.106647	Ineligible study type
Karimzadeh L, Behrouz V, Sohrab G, Hedayati M, Emami G. 2022. A randomized clinical trial of beetroot juice consumption on inflammatory markers and oxidative stress in patients with type 2 diabetes. PMID: 36342289. doi:10.1111/1750-3841.16365	Study did not include predetermined outcomes, related to diabetic complications
Amini P, Sajedi F, Mirjalili M, Mohammadi Y, Mehrpooya M. 2022. Coenzyme Q10 as a potential add-on treatment for patients suffering from painful diabetic neuropathy: results of a placebo-controlled randomized trial. PMID: 36273066. doi:10.1007/s00228-022-03407-x	Article could not be accessed
Karimzadeh L, Sohrab G, Hedayati M, Ebrahimof S, Emami G, Razavion T. 2023. Effects of concentrated beetroot juice consumption on glycemic control, blood pressure, and lipid profile in type 2 diabetes patients: randomized clinical trial study. PMID: 35869311. doi:10.1007/s11845-022-03090-y	Study did not include predetermined outcomes, related to diabetic complications
Huo J, Xue Y, Dong X, Lv J, Wu L, Gao H, Yang X, Liu H, Gao Q. 2023. Efficacy of vitamin and antioxidant supplements for treatment of diabetic peripheral neuropathy: systematic review and meta-analysis of randomized controlled trials. PMID: 35816410. doi:10.1080/1028415X.2022.2090606	Ineligible study type
Ou T, Wang W, Yong H, Hao H, Wang R, Dai X, Wang D, Li Y. 2022. Liraglutide Plus Dapagliflozin for High Uric Acid and Microalbuminuria in Diabetes Mellitus Complicated With Metabolic Syndrome. PMID: 35751892. doi:	Study did not include predetermined outcomes, related to diabetic complications
Guo Y, Liu S, Xu H. 2022. Uric Acid and Diabetic Retinopathy: A Systematic Review and Meta-Analysis. PMID: 35712295. doi:10.3389/fpubh.2022.906760	Ineligible study type
Kharitonova T, Shvarts YG, Verbovoy AF, Orlova NS, Puzyreva VP, Strokov IA. 2022. Efficacy and safety of the combined metabolic medication, containing inosine, nicotinamide, riboflavin and succinic acid, for the treatment of diabetic neuropathy: a multicenter randomized, double-blind, placebo-controlled parallel group clinical trial (CYLINDER). PMID: 35680173. doi:10.1136/bmjdrc-2022-002785	Study did not include predetermined outcomes, related to diabetic complications
Dai Y, Quan J, Xiong L, Luo Y, Yi B. 2022. Probiotics improve renal function, glucose, lipids, inflammation and oxidative stress in diabetic kidney disease: a systematic review and meta-analysis. PMID: 35611435. doi:10.1080/0886022X.2022.2079522	Ineligible study type
Segar MW, Kolkailah AA, Frederich R, Pong A, Cannon CP, Cosentino F, Dagogo-Jack S, McGuire DK, Pratley RE, Liu CC, Maldonado M, Liu J, Cater NB, Pandey A, Cherney DZI. 2022. Mediators of ertugliflozin effects on heart failure and kidney outcomes among patients with type 2 diabetes mellitus. PMID: 35603908. doi:10.1111/dom.14769	Study did not include predetermined outcomes, related to diabetic complications
Macena ML, Nunes LFDS, da Silva AF, Pureza IROM, Praxedes DRS, Santos JCF, Bueno NB. 2022. Effects of dietary polyphenols in the glycemic, renal, inflammatory, and oxidative stress biomarkers in diabetic nephropathy: a	Ineligible study type

systematic review with meta-analysis of randomized controlled trials. PMID: 35595310. doi:10.1093/nutrit/nuac035	
Ali Sangouni A, Abdollahi S, Mozaffari-Khosravi H. 2022. Effect of resveratrol supplementation on hepatic steatosis and cardiovascular indices in overweight subjects with type 2 diabetes: a double-blind, randomized controlled trial. PMID: 35538431. doi:10.1186/s12872-022-02637-2	Study did not include predetermined outcomes, related to diabetic complications
Bakris GL, Mikami H, Hirata M, Nakajima A, Cressman MD. 2021. A Non-purine Xanthine Oxidoreductase Inhibitor Reduces Albuminuria in Patients with DKD: A Randomized Controlled Trial. PMID: 35369650. doi:10.34067/KID.0001672021	Drug effect study
Talari HR, Tabatabaei SMH, Soleimani A, Sayyah M, Bozorgi M. 2022. The effects of melatonin administration on carotid intima-media thickness and pulse wave velocity in diabetic nephropathy: A randomized, double-blind, placebo-controlled trial. PMID: 35331538. doi:10.1016/j.clnesp.2022.01.002	Article could not be accessed
Suijk DLS, van Baar MJB, van Bommel EJM, Iqbal Z, Krebber MM, Vallon V, Touw D, Hoorn EJ, Nieuwdorp M, Kramer MMH, Joles JA, Bjornstad P, van Raalte DH. 2022. SGLT2 Inhibition and Uric Acid Excretion in Patients with Type 2 Diabetes and Normal Kidney Function. PMID: 35322793. doi:10.2215/CJN.11480821	Study did not include predetermined outcomes, related to diabetic complications
Zhong O, Hu J, Wang J, Tan Y, Hu L, Lei X. 2022. Antioxidant for treatment of diabetic complications: A meta-analysis and systematic review. PMID: 35307907. doi:10.1002/jbt.23038	Ineligible study type
Ji P, Zhu J, Feng J, Li H, Yu Q, Qin H, Wei L, Zhang J. 2022. Serum uric acid levels and diabetic kidney disease in patients with type 2 diabetes mellitus: A dose-response meta-analysis. PMID: 35305901. doi:10.1016/j.pcd.2022.03.003	Ineligible study type
Tabaei BS, Mousavi SN, Rahimian A, Rostamkhani H, Mellati AA, Jameshorani M. 2022. Co-Administration of Vitamin E and Atorvastatin Improves Insulin Sensitivity and Peroxisome Proliferator-Activated Receptor-γ Expression in Type 2 Diabetic Patients: A Randomized Double-Blind Clinical Trial. PMID: 35291435. doi:10.30476/ijms.2021.89102.1981	Study did not include predetermined outcomes, related to diabetic complications
Grabež M, Škrbić R, Stojiljković MP, Vučić V, Rudić Grujić V, Jakovljević V, Djuric DM, Suručić R, Šavikin K, Bigović D, Vasiljević N. 2022. A prospective, randomized, double-blind, placebo-controlled trial of polyphenols on the outcomes of inflammatory factors and oxidative stress in patients with type 2 diabetes mellitus. PMID: 35229548. doi:10.31083/j.rcm2302057	Study did not include predetermined outcomes, related to diabetic complications
Wu B, Chen L, Xu Y, Duan Q, Zheng Z, Zheng Z, He D. 2022. The Effect of Allopurinol on Renal Outcomes in Patients with Diabetic Kidney Disease: A Systematic Review and Meta-Analysis. PMID: 35130544. doi:10.1159/000522248	Ineligible study type
Xiong R, Yuan Y, Zhu Z, Wu Y, Ha J, Han X, Wang W, He M. 2022. Micronutrients and Diabetic Retinopathy: Evidence From The National Health and Nutrition Examination Survey and a Meta-analysis. PMID: 35033539. doi:10.1016/j.ajo.2022.01.005	Article could not be accessed
Abdelhaleem IA, Brakat AM, Adayel HM, Asla MM, Rizk MA, Aboalfetoh AY. 2022. The effects of resveratrol on glycemic control and cardiometabolic parameters in patients with T2DM: A systematic review and meta-analysis. PMID: 34666902. doi:10.1016/j.medcli.2021.06.028	Ineligible study type

Cojic M, Kocic R, Klisic A, Kocic G. 2021. The Effects of Vitamin D Supplementation on Metabolic and Oxidative Stress Markers in Patients With Type 2 Diabetes: A 6-Month Follow Up Randomized Controlled Study. PMID: 34489860. doi:10.3389/fendo.2021.610893	Study did not include predetermined outcomes, related to diabetic complications
Sun F, Jiang D, Cai J. 2021. Effects of valsartan combined with α-lipoic acid on renal function in patients with diabetic nephropathy: a systematic review and meta-analysis. PMID: 34465338. doi:10.1186/s12902-021-00844-0	Ineligible study type
Leehey DJ, Carlson K, Reda DJ, Craig I, Clise C, Conner TA, Agarwal R, Kaufman JS, Anderson RJ, Lammie D, Huminik J, Polzin L, McBurney C, Huang GD, Emanuele NV. 2021. Pentoxifylline in diabetic kidney disease (VA PTXRx): protocol for a pragmatic randomised controlled trial. PMID: 34400461. doi:10.1136/bmjopen-2021-053019	Drug effect study
Shokri M, Sajedi F, Mohammadi Y, Mehrpooya M. 2021. Adjuvant use of melatonin for relieving symptoms of painful diabetic neuropathy: results of a randomized, double-blinded, controlled trial. PMID: 34121140. doi:10.1007/s00228-021-03170-5	Study did not include predetermined outcomes, related to diabetic complications
Mahjabeen W, Khan DA, Mirza SA, Pervez MA. 2021. Effects of delta-tocotrienol supplementation on Glycemic Control, oxidative stress, inflammatory biomarkers and miRNA expression in type 2 diabetes mellitus: A randomized control trial. PMID: 33899292. doi:10.1002/ptr.7113	Study did not include predetermined outcomes, related to diabetic complications
Behrouz V, Sohrab G, Hedayati M, Sedaghat M. 2021. Inflammatory markers response to crocin supplementation in patients with type 2 diabetes mellitus: A randomized controlled trial. PMID: 33856733. doi:10.1002/ptr.7124	Study did not include predetermined outcomes, related to diabetic complications
Venugopal V, Geethanjali S, Poonguzhali S, Padmavathi R, Mahadevan S, Silambanan S, Maheshkumar K. 2022. Effect of Yoga on Oxidative Stress in Type 2 Diabetes Mellitus: A Systematic Review and Meta-analysis. PMID: 33820522. doi:10.2174/1573399817666210405104335	Ineligible study type
Bazyar H, Zare Javid A, Bavi Behbahani H, Moradi F, Moradi Poode B, Amiri P. 2021. Consumption of melatonin supplement improves cardiovascular disease risk factors and anthropometric indices in type 2 diabetes mellitus patients: a double-blind, randomized, placebo-controlled trial. PMID: 33766084. doi:10.1186/s13063-021-05174-z	Study did not include predetermined outcomes, related to diabetic complications
Satari M, Bahmani F, Reiner Z, Soleimani A, Aghadavod E, Kheiripour N, Asemi Z. 2021. Metabolic and Anti-inflammatory Response to Melatonin Administration in Patients with Diabetic Nephropathy. PMID: 33492301. doi:	Study did not include predetermined outcomes, related to diabetic complications
Mason SA, Keske MA, Wadley GD. 2021. Effects of Vitamin C Supplementation on Glycemic Control and Cardiovascular Risk Factors in People With Type 2 Diabetes: A GRADE-Assessed Systematic Review and Meta-analysis of Randomized Controlled Trials. PMID: 33472962. doi:10.2337/dc20-1893	Ineligible study type
Guan H, Ye M, Fang C, Zhang L, Han P, Qiu S, Fang X, Li L. 2020. The clinical effectiveness and safety of alprostadil combined with alpha lipoic acid in the treatment of diabetic peripheral neuropathy: A protocol for systematic review and meta-analysis. PMID: 33327292. doi:10.1097/MD.0000000000023507	Ineligible study type

AbdelQadir YH, Hamdallah A, Sibaey EA, Hussein AS, Abdelaziz M, AbdelAzim A, Ragab KM, Helmy SK, Nourelden AZ. 2020. Efficacy of probiotic supplementation in patients with diabetic nephropathy: A systematic review and meta-analysis. PMID: 33183573. doi:10.1016/j.clnesp.2020.06.019	Ineligible study type
Zitouni K, Steyn MRCP, Lyka E, Kelly FJ, Cook P, Ster IC, Earle KA. 2020. Derepression of glomerular filtration, renal blood flow and antioxidant defence in patients with type 2 diabetes at high-risk of cardiorenal disease. PMID: 33039650. doi:10.1016/j.freeradbiomed.2020.10.005	Study did not include predetermined outcomes, related to diabetic complications
Gunton JE, Girgis CM, Lau T, Vicaretti M, Begg L, Flood V. 2021. Vitamin C improves healing of foot ulcers: a randomised, double-blind, placebo-controlled trial. PMID: 32981536. doi:10.1017/S0007114520003815	Study conducted on non-target group of patients (non-diabetic, diabetes type 1 etc.)
Shimizu W, Kubota Y, Hoshika Y, Mozawa K, Tara S, Tokita Y, Yodogawa K, Iwasaki YK, Yamamoto T, Takano H, Tsukada Y, Asai K, Miyamoto M, Miyauchi Y, Kodani E, Ishikawa M, Maruyama M, Ogano M, Tanabe J; EMBODY trial investigators. 2020. Effects of empagliflozin versus placebo on cardiac sympathetic activity in acute myocardial infarction patients with type 2 diabetes mellitus: the EMBODY trial. PMID: 32977831. doi:10.1186/s12933-020-01127-z	Drug effect study
Poulsen NB, Lambert MNT, Jeppesen PB. 2020. The Effect of Plant Derived Bioactive Compounds on Inflammation: A Systematic Review and Meta-Analysis. PMID: 32761736. doi:10.1002/mnfr.202000473	Ineligible study type
Pingali U, Sukumaran D, Nutalapati C. 2020. Effect of an aqueous extract of Terminalia chebula on endothelial dysfunction, systemic inflammation, and lipid profile in type 2 diabetes mellitus: A randomized double-blind, placebo-controlled clinical study. PMID: 32618037. doi:10.1002/ptr.6771	Study did not include predetermined outcomes, related to diabetic complications
Shi J, Cheng Z, Qiu S, Cui H, Gu Y, Zhao Q, Ren Y, Zhang H, Sun H, Liu Y, Li Y, Qiao Y, Hu Y, Liu Y, Cheng Y. 2020. ε2 allele and ε2-involved genotypes (ε2/ε2, ε2/ε3, and ε2/ε4) may confer the association of APOE genetic polymorphism with risks of nephropathy in type 2 diabetes: a meta-analysis. PMID: 32534589. doi:10.1186/s12944-020-01307-6	Study did not include predetermined outcomes, related to diabetic complications
Batista-Jorge GC, Barcala-Jorge AS, Silveira MF, Lelis DF, Andrade JMO, de Paula AMB, Guimarães ALS, Santos SHS. 2020. Oral resveratrol supplementation improves Metabolic Syndrome features in obese patients submitted to a lifestyle-changing program. PMID: 32534040. doi:10.1016/j.lfs.2020.117962	Study did not include predetermined outcomes, related to diabetic complications
El-Nahas MR, Elkannishy G, Abdelhafez H, Elkhamisy ET, El-Sehrawy AA. 2020. Oral Alpha Lipoic Acid Treatment for Symptomatic Diabetic Peripheral Neuropathy: A Randomized Double-Blinded Placebo-Controlled Study. PMID: 32370731. doi:10.2174/1871530320666200506081407	Article could not be accessed
Golpour P, Nourbakhsh M, Mazaherioun M, Janani L, Nourbakhsh M, Yaghmaei P. 2020. Improvement of NRF2 gene expression and antioxidant status in patients with type 2 diabetes mellitus after supplementation with omega-3 polyunsaturated fatty acids: A double-blind randomised placebo-controlled clinical trial. PMID: 32194222. doi:10.1016/j.diabres.2020.108120	Study did not include predetermined outcomes, related to diabetic complications
Dludla PV, Silvestri S, Orlando P, Gabuza KB, Mazibuko-Mbeje SE, Nyambuya TM, Mxinwa V, Mokgalaboni K, Johnson R, Muller CJF, Tiano L, Louw J, Nkambule BB. 2020. Exploring the Comparative Efficacy of Metformin and Resveratrol in the	Ineligible study type

Management of Diabetes-associated Complications: A Systematic Review of Preclinical Studies. PMID: 32168855. doi:10.3390/nu12030739	
Çetinkalp Ş, Gökçe EH, Şimşir I, Tuncay Tanrıverdi S, Doğan F, Biray Avcı Ç, Eroğlu İ, Utku T, Gündüz C, Özer Ö. 2021. Comparative Evaluation of Clinical Efficacy and Safety of Collagen Laminin-Based Dermal Matrix Combined With Resveratrol Microparticles (Dermalix) and Standard Wound Care for Diabetic Foot Ulcers. PMID: 32131656. doi:10.1177/1534734620907773	Study did not include predetermined outcomes, related to diabetic complications
Tanaka M, Yamakage H, Inoue T, Odori S, Kusakabe T, Shimatsu A, Satoh-Asahara N. 2020. Beneficial Effects of Ipragliflozin on the Renal Function and Serum Uric Acid Levels in Japanese Patients with Type 2 Diabetes: A Randomized, 12-week, Open-label, Active-controlled Trial. PMID: 32115517. doi:10.2169/internalmedicine.3473-19	Drug effect study
Verma S, Ji Q, Bhatt DL, Mazer CD, Al-Omran M, Inzucchi SE, Wanner C, Ofstad AP, Zwiener I, George JT, Zinman B, Fitchett D. 2020. Association between uric acid levels and cardio-renal outcomes and death in patients with type 2 diabetes: A subanalysis of EMPA-REG OUTCOME. PMID: 32030863. doi:10.1111/dom.13991	Study did not include predetermined outcomes, related to diabetic complications
Matter RM, Elbarbary NS, Ismail EAR, Darwish YW, Nada AS, Banoub VP. 2020. Zinc supplementation improves glucose homeostasis in patients with β-thalassemia major complicated with diabetes mellitus: A randomized controlled trial. PMID: 32007694. doi:10.1016/j.nut.2019.110702	Study did not include predetermined outcomes, related to diabetic complications
Gallelli G, Cione E, Serra R, Leo A, Citraro R, Matricardi P, Di Meo C, Bisceglia F, Caroleo MC, Basile S, Gallelli L. 2020. Nano-hydrogel embedded with quercetin and oleic acid as a new formulation in the treatment of diabetic foot ulcer: A pilot study. PMID: 31876118. doi:10.1111/iwj.13299	Study did not include predetermined outcomes, related to diabetic complications
Farhangi MA, Dehghan P, Namazi N. 2020. Prebiotic supplementation modulates advanced glycation end-products (AGEs), soluble receptor for AGEs (sRAGE), and cardiometabolic risk factors through improving metabolic endotoxemia: a randomized-controlled clinical trial. PMID: 31728681. doi:10.1007/s00394-019-02140-z	Study did not include predetermined outcomes, related to diabetic complications
Jiang Z, Tan Z, Meng F, Li X. 2019. Curative Effects of Valsartan Alone or Combined with Alpha-lipoic Acid on Inflammatory Cytokines and Renal Function in Early-stage Diabetic Kidney Disease. PMID: 31564281. doi:10.29271/jcpsp.2019.10.1009	Article could not be accessed
Shao Y, Shao H, Sawhney MS, Shi L. 2019. Serum uric acid as a risk factor of all-cause mortality and cardiovascular events among type 2 diabetes population: Meta-analysis of correlational evidence. PMID: 31439471. doi:10.1016/j.jdiacomp.2019.07.006	Study did not include predetermined outcomes, related to diabetic complications
Srinivasan A, Selvarajan S, Kamalanathan S, Kadhiravan T, Prasanna Lakshmi NC, Adithan S. 2019. Effect of Curcuma longa on vascular function in native Tamilians with type 2 diabetes mellitus: A randomized, double-blind, parallel arm, placebo-controlled trial. PMID: 31155769. doi:10.1002/ptr.6381	Study did not include predetermined outcomes, related to diabetic complications
Nazem MR, Asadi M, Jabbari N, Allameh A. 2019. Effects of zinc supplementation on superoxide dismutase activity and gene expression, and metabolic parameters in overweight type 2 diabetes patients: A randomized, double-blind, controlled trial. PMID: 31129183. doi:10.1016/j.clinbiochem.2019.05.008	Study did not include predetermined outcomes, related to diabetic complications

Farajbakhsh A, Mazloomi SM, Mazidi M, Rezaie P, Akbarzadeh M, Ahmad SP, Ferns GA, Ofori-Asenso R, Babajafari S. 2019. Sesame oil and vitamin E co-administration may improve cardiometabolic risk factors in patients with metabolic syndrome: a randomized clinical trial. PMID: 31089253. doi:10.1038/s41430-019-0438-5	Study did not include predetermined outcomes, related to diabetic complications
Dong L, Li J, Lian Y, Tang ZX, Zen Z, Yu P, Li Y. 2019. Long-Term Intensive Lifestyle Intervention Promotes Improvement of Stage III Diabetic Nephropathy. PMID: 31022160. doi:10.12659/MSM.913512	Study conducted on non-target group of patients (non-diabetic, diabetes type 1 etc.)
de Morales AM, Goicoechea M, Verde E, Carbayo J, Barbieri D, Delgado A, Verdalles U, de Jose AP, Luño J. 2019. Pentoxifylline, progression of chronic kidney disease (CKD) and cardiovascular mortality: long-term follow-up of a randomized clinical trial. PMID: 30949987. doi:10.1007/s40620-019-00607-0	Drug effect study
Afsharpour F, Javadi M, Hashemipour S, Koushan Y, Haghighian HK. 2019. Propolis supplementation improves glycemic and antioxidant status in patients with type 2 diabetes: A randomized, double-blind, placebo-controlled study. PMID: 30935545. doi:10.1016/j.ctim.2019.03.001	Study did not include predetermined outcomes, related to diabetic complications
Adibian M, Hodaei H, Nikpayam O, Sohrab G, Hekmatdoost A, Hedayati M. 2019. The effects of curcumin supplementation on high-sensitivity C-reactive protein, serum adiponectin, and lipid profile in patients with type 2 diabetes: A randomized, double-blind, placebo-controlled trial. PMID: 30864188. doi:10.1002/ptr.6328	Study did not include predetermined outcomes, related to diabetic complications
Aslanabadi N, Afsar Gharebagh R, Moharramzadeh S, Entezari-Maleki T. 2019. Pentoxifylline for the prevention of contrast-induced nephropathy in diabetic patients undergoing angioplasty: a randomized controlled trial. PMID: 30830654. doi:10.1007/s11255-019-02104-6	Drug effect study
Afzali H, Jafari Kashi AH, Momen-Heravi M, Razzaghi R, Amirani E, Bahmani F, Gilasi HR, Asemi Z. 2019. The effects of magnesium and vitamin E co-supplementation on wound healing and metabolic status in patients with diabetic foot ulcer: A randomized, double-blind, placebo-controlled trial. PMID: 30693609. doi:10.1111/wrr.12701	Study did not include predetermined outcomes, related to diabetic complications
Van den Eynde MDG, Geleijnse JM, Scheijen JLJM, Hanssen NMJ, Dower JI, Afman LA, Stehouwer CDA, Hollman PCH, Schalkwijk CG. 2018. Quercetin, but Not Epicatechin, Decreases Plasma Concentrations of Methylglyoxal in Adults in a Randomized, Double-Blind, Placebo-Controlled, Crossover Trial with Pure Flavonoids. PMID: 30398646. doi:10.1093/jn/nxy236	Study conducted on non-target group of patients (non-diabetic, diabetes type 1 etc.)
Karkabounas S, Papadopoulos N, Anastasiadou C, Gubili C, Peschos D, Daskalou T, Fikioris N, Simos YV, Kontargiris E, Gianakopoulos X, Ragos V, Chatzidimitriou M. 2018. Effects of α-Lipoic Acid, Carnosine, and Thiamine Supplementation in Obese Patients with Type 2 Diabetes Mellitus: A Randomized, Double-Blind Study. PMID: 30311825. doi:10.1089/jmf.2018.0007	Study did not include predetermined outcomes, related to diabetic complications
Zarezadeh M, Saedisomeolia A, Khorshidi M, Kord Varkane H, Makhdoomi Arzati M, Abdollahi M, Yekaninejad MS, Hashemi R, Effatpanah M, Mohammadzadeh Honarvar N. 2018. Asymmetric dimethylarginine and soluble inter-cellular adhesion molecule-1 serum levels alteration following ginger supplementation in patients with type 2 diabetes: a randomized double-blind, placebo-controlled clinical trial. PMID: 30099412. doi:10.1515/jcim-2018-0019	Study did not include predetermined outcomes, related to diabetic complications

Wang J, Yu Y, Li X, Li D, Xu C, Yuan J, Wei S, Li X, Yang K, Zheng D, Tang Y, Yang H, Wu T, He M. 2018. Serum uric acid levels and decreased estimated glomerular filtration rate in patients with type 2 diabetes: A cohort study and meta-analysis. PMID: 30003679. doi:10.1002/dmrr.3046	Study did not include predetermined outcomes, related to diabetic complications
Jiang DQ, Xu LC, Jiang LL, Li MX, Wang Y. 2018. Fasudil combined with methylcobalamin or lipoic acid can improve the nerve conduction velocity in patients with diabetic peripheral neuropathy: A meta-analysis. PMID: 29979431. doi:10.1097/MD.0000000000011390	Drug effect study
Khodabandehloo H, Seyyedebrahimi S, Esfahani EN, Razi F, Meshkani R. 2018. Resveratrol supplementation decreases blood glucose without changing the circulating CD14(+)CD16(+) monocytes and inflammatory cytokines in patients with type 2 diabetes: a randomized, double-blind, placebo-controlled study. PMID: 29914666. doi:10.1016/j.nutres.2018.03.015	Study did not include predetermined outcomes, related to diabetic complications
Navarro-González JF, Sánchez-Niño MD, Donate-Correa J, Martín-Núñez E, Ferri C, Pérez-Delgado N, Górriz JL, Martínez-Castelao A, Ortiz A, Mora-Fernández C. 2018. Effects of Pentoxifylline on Soluble Klotho Concentrations and Renal Tubular Cell Expression in Diabetic Kidney Disease. PMID: 29866645. doi:10.2337/dc18-0078	Drug effect study
Rabizadeh S, Dehghani Firouzabadi F, Noshad S, Esteghamati S, Afarideh M, Ghajar A, Ganji M, Saadat M, Heidari B, Najafi MT, Nakhjavani M, Esteghamati A. 2018. Beneficial Effects of Pentoxifylline Plus Losartan Dual Therapy in Type 2 Diabetes with Nephropathy. PMID: 29753374. doi:10.1016/j.amjms.2017.12.015	Drug effect study
Fayh APT, Borges K, Cunha GS, Krause M, Rocha R, de Bittencourt PIH Jr, Moreira JCF, Friedman R, da Silva Rossato J, Fernandes JR, Reischak-Oliveira A. 2018. Effects of n-3 fatty acids and exercise on oxidative stress parameters in type 2 diabetic: a randomized clinical trial. PMID: 29713249. doi:10.1186/s12970-018-0222-2	Study did not include predetermined outcomes, related to diabetic complications
Edmonds ME, Bodansky HJ, Boulton AJM, Chadwick PJ, Dang CN, D'Costa R, Johnston A, Kennon B, Leese G, Rajbhandari SM, Serena TE, Young MJ, Stewart JE, Tucker AT, Carter MJ. 2018. Multicenter, randomized controlled, observer-blinded study of a nitric oxide generating treatment in foot ulcers of patients with diabetes-ProNOx1 study. PMID: 29617058. doi:10.1111/wrr.12630	Study design did not include administration of antioxidants
Carlström M, Larsson SC. 2018. Coffee consumption and reduced risk of developing type 2 diabetes: a systematic review with meta-analysis. PMID: 29590460. doi:10.1093/nutrit/nuy014	Ineligible study type
El-Aal AA, El-Ghffar EAA, Ghali AA, Zughbur MR, Sirdah MM. 2018. The effect of vitamin C and/or E supplementations on type 2 diabetic adult males under metformin treatment: A single-blinded randomized controlled clinical trial. PMID: 29571976. doi:10.1016/j.dsx.2018.03.013	Study did not include predetermined outcomes, related to diabetic complications
Sepahi S, Mohajeri SA, Hosseini SM, Khodaverdi E, Shoeibi N, Namdari M, Tabassi SAS. 2018. Effects of Crocin on Diabetic Maculopathy: A Placebo-Controlled Randomized Clinical Trial. PMID: 29550187. doi:10.1016/j.ajo.2018.03.007	Study conducted on non-target group of patients (non-diabetic, diabetes type 1 etc.)
Homayouni F, Haidari F, Hedayati M, Zakerkish M, Ahmadi K. 2018. Blood pressure lowering and anti-inflammatory effects of hesperidin in type 2 diabetes; a randomized double-blind controlled clinical trial. PMID: 29468764. doi:10.1002/ptr.6046	Study did not include predetermined outcomes, related to diabetic complications

Panahi Y, Khalili N, Sahebi E, Namazi S, Simental-Mendía LE, Majeed M, Sahebkar A. 2018. Effects of Curcuminoids Plus Piperine on Glycemic, Hepatic and Inflammatory Biomarkers in Patients with Type 2 Diabetes Mellitus: A Randomized Double-Blind Placebo-Controlled Trial. PMID: 29458218. doi:10.1055/s-0044-101752	Study did not include predetermined outcomes, related to diabetic complications
Raygan F, Ostadmohammadi V, Bahmani F, Asemi Z. 2018. The effects of vitamin D and probiotic co-supplementation on mental health parameters and metabolic status in type 2 diabetic patients with coronary heart disease: A randomized, double-blind, placebo-controlled trial. PMID: 29432877. doi:10.1016/j.pnpbp.2018.02.007	Study design did not include administration of antioxidants
Mashhadi NS, Zakerkish M, Mohammadiasl J, Zarei M, Mohammadshahi M, Haghighizadeh MH. 2018. Astaxanthin improves glucose metabolism and reduces blood pressure in patients with type 2 diabetes mellitus. PMID: 29384321. doi:10.6133/apjcn.052017.11	Study did not include predetermined outcomes, related to diabetic complications
Vitamin E in Neuroprotection Study (VENUS) Investigators; Hor CP, Fung WY, Ang HA, Lim SC, Kam LY, Sim SW, Lim LH, Choon WY, Wong JW, Ch'ng ASH, Beh KKM, Wee HC, Ong LM, Khan NAK, Sulaiman SAS, Shuaib IL, Bakar A, Yusof Y, Yusof YM, Abu Bakar F, Tang WS, Teh HL, Wahid NA, Saaidin S, Idris N, Yoon CK, Ong HN, Ganapathy JT, Loo CE, Samy MM, Zainal H, Dharan SCS, Ooi BY, Teoh PY, Tye YL, Yeoh CA, Low DW, Looi I, Yuen KH. 2018. Efficacy of Oral Mixed Tocotrienols in Diabetic Peripheral Neuropathy: A Randomized Clinical Trial. PMID: 29379943. doi:10.1001/jamaneurol.2017.4609	Study conducted on non-target group of patients (non-diabetic, diabetes type 1 etc.)
Wada T, Hosoya T, Honda D, Sakamoto R, Narita K, Sasaki T, Okui D, Kimura K. 2018. Uric acid-lowering and renoprotective effects of topiroxostat, a selective xanthine oxidoreductase inhibitor, in patients with diabetic nephropathy and hyperuricemia: a randomized, double-blind, placebo-controlled, parallel-group study (UPWARD study). PMID: 29372470. doi:10.1007/s10157-018-1530-1	Drug effect study
Fogacci F, Tocci G, Presta V, Fratter A, Borghi C, Cicero AFG. 2019. Effect of resveratrol on blood pressure: A systematic review and meta-analysis of randomized, controlled, clinical trials. PMID: 29359958. doi:10.1080/10408398.2017.1422480	Ineligible study type
Dandona P, Ghanim H, Abuaysheh S, Green K, Dhindsa S, Makdissi A, Batra M, Kuhadiya ND, Chaudhuri A. 2018. Exenatide Increases IL-1RA Concentration and Induces Nrf-2–Keap-1–Regulated Antioxidant Enzymes: Relevance to β-Cell Function. PMID: 29346597. doi:10.1210/jc.2017-02343	Drug effect study
Pilemann-Lyberg S, Persson F, Frystyk J, Rossing P. 2018. The effect of uric acid lowering treatment on albuminuria and renal function in Type 1 diabetes: a randomized clinical trial. PMID: 29315768. doi:10.1111/dme.13577	Study conducted on non-target group of patients (non-diabetic, diabetes type 1 etc.)
Mohseni S, Bayani M, Bahmani F, Tajabadi-Ebrahimi M, Bayani MA, Jafari P, Asemi Z. 2018. The beneficial effects of probiotic administration on wound healing and metabolic status in patients with diabetic foot ulcer: A randomized, double-blind, placebo-controlled trial. PMID: 29193662. doi:10.1002/dmrr.2970	Study conducted on non-target group of patients (non-diabetic, diabetes type 1 etc.)
Wang XT, Lin HX, Xu SA, Lu YK. 2017. Lipoic Acid Combined with Epalrestat versus Lipoic Acid in Treating Diabetic Peripheral Neuropathy:A Meta-analysis. PMID: 29125108. doi:10.3881/j.issn.1000-503X.2017.05.010	Article could not be accessed

Kandhare AD, Mukherjee A, Bodhankar SL. 2017. Antioxidant for treatment of diabetic nephropathy: A systematic review and meta-analysis. PMID: 29108777. doi:10.1016/j.cbi.2017.10.031	Ineligible study type
Voroneanu L, Siriopol D, Dumea R, Badarau S, Kanbay M, Afsar B, Gavrilovici C, Covic A. 2017. Addition of silymarin to renin-angiotensin system blockers in normotensive patients with type 2 diabetes mellitus and proteinuria: a prospective randomized trial. PMID: 28948420. doi:10.1007/s11255-017-1697-5	Study design did not include administration of antioxidants
Gillani SW, Sulaiman SAS, Abdul MIM, Baig MR. 2017. Combined effect of metformin with ascorbic acid versus acetyl salicylic acid on diabetes-related cardiovascular complication; a 12-month single blind multicenter randomized control trial. PMID: 28807030. doi:10.1186/s12933-017-0584-9	Drug effect study
Mirmiran P, Hosseinpour-Niazi S, Azizi F. 2018. Therapeutic lifestyle change diet enriched in legumes reduces oxidative stress in overweight type 2 diabetic patients: a crossover randomised clinical trial. PMID: 28722030. doi:10.1038/ejcn.2017.113	Study did not include predetermined outcomes, related to diabetic complications
Bolignano D, Cernaro V, Gembillo G, Baggetta R, Buemi M, D'Arrigo G. 2017. Antioxidant agents for delaying diabetic kidney disease progression: A systematic review and meta-analysis. PMID: 28570649. doi:10.1371/journal.pone.0178699	Ineligible study type
Razzaghi R, Pidar F, Momen-Heravi M, Bahmani F, Akbari H, Asemi Z. 2018. Magnesium Supplementation and the Effects on Wound Healing and Metabolic Status in Patients with Diabetic Foot Ulcer: a Randomized, Double-Blind, Placebo-Controlled Trial. PMID: 28540570. doi:10.1007/s12011-017-1056-5	Study conducted on non-target group of patients (non-diabetic, diabetes type 1 etc.)
Momen-Heravi M, Barahimi E, Razzaghi R, Bahmani F, Gilasi HR, Asemi Z. 2017. The effects of zinc supplementation on wound healing and metabolic status in patients with diabetic foot ulcer: A randomized, double-blind, placebo-controlled trial. PMID: 28395131. doi:10.1111/wrr.12537	Study conducted on non-target group of patients (non-diabetic, diabetes type 1 etc.)
Sucher S, Markova M, Hornemann S, Pivovarova O, Rudovich N, Thomann R, Schneeweiss R, Rohn S, Pfeiffer AFH. 2017. Comparison of the effects of diets high in animal or plant protein on metabolic and cardiovascular markers in type 2 diabetes: A randomized clinical trial. PMID: 28181738. doi:10.1111/dom.12901	Study did not include predetermined outcomes, related to diabetic complications
Zhu B, Wu X, Bi Y, Yang Y. 2017. Effect of bilirubin concentration on the risk of diabetic complications: A meta-analysis of epidemiologic studies. PMID: 28134328. doi:10.1038/srep41681	Study did not include predetermined outcomes, related to diabetic complications
Sun H, Yao W, Tang Y, Zhuang W, Wu D, Huang S, Sheng H. 2017. Urinary exosomes as a novel biomarker for evaluation of α-lipoic acid's protective effect in early diabetic nephropathy. PMID: 28116765. doi:10.1002/jcla.22129	Study conducted on non-target group of patients (non-diabetic, diabetes type 1 etc.)
Gasca-Lozano LE, Lucano-Landeros S, Ruiz-Mercado H, Salazar-Montes A, Sandoval-Rodríguez A, Garcia-Bañuelos J, Santos-Garcia A, Davila-Rodriguez JR, Navarro-Partida J, Bojórquez-Sepúlveda H, Castañeda-Gomez J, Domínguez-Rosales J, Ruiz-Arcos MA, Sánchez-Parada MG, Armendariz-Borunda J. 2017. Pirfenidone Accelerates Wound Healing in Chronic Diabetic Foot Ulcers: A Randomized, Double-Blind Controlled Trial. PMID: 29675430. doi:10.1155/2017/3159798	Drug effect study

Borgi L, McMullan C, Wohlhueter A, Curhan GC, Fisher ND, Forman JP. 2017. Effect of Uric Acid-Lowering Agents on Endothelial Function: A Randomized, Double-Blind, Placebo-Controlled Trial. PMID: 28028194. doi:10.1161/HYPERTENSIONAHA.116.08488	Drug effect study
Soleimani A, Zarrati Mojarrad M, Bahmani F, Taghizadeh M, Ramezani M, Tajabadi-Ebrahimi M, Jafari P, Esmaillzadeh A, Asemi Z. 2017. Probiotic supplementation in diabetic hemodialysis patients has beneficial metabolic effects. PMID: 27927601. doi:10.1016/j.kint.2016.09.040	Study conducted on non-target group of patients (non-diabetic, diabetes type 1 etc.)
Han Y, Wang M, Shen J, Zhang Z, Zhao M, Huang J, Chen Y, Chen Z, Hu Y, Wang Y. 2018. Differential efficacy of methylcobalamin and alpha-lipoic acid treatment on symptoms of diabetic peripheral neuropathy. PMID: 27901334. doi:10.23736/S0391-1977.16.02505-0	Study design did not include administration of antioxidants
Lafuente M, Ortín L, Argente M, Guindo JL, López-Bernal MD, López-Román FJ, García MJ, Domingo JC, Lajara J. 2017. COMBINED INTRAVITREAL RANIBIZUMAB AND ORAL SUPPLEMENTATION WITH DOCOSAHEXAENOIC ACID AND ANTIOXIDANTS FOR DIABETIC MACULAR EDEMA: Two-Year Randomized Single-Blind Controlled Trial Results. PMID: 27787443. doi:10.1097/IAE.0000000000001363	Study did not include predetermined outcomes, related to diabetic complications
Stonehouse W, Brinkworth GD, Thompson CH, Abeywardena MY. 2016. Short term effects of palm-tocotrienol and palm-carotenes on vascular function and cardiovascular disease risk: A randomised controlled trial. PMID: 27760402. doi:10.1016/j.atherosclerosis.2016.10.027	Study did not include predetermined outcomes, related to diabetic complications
Du L, Ma J, Zhang X. 2017. Higher Serum Uric Acid May Contribute to Cerebral Infarction in Patients with Type 2 Diabetes Mellitus: a Meta-Analysis. PMID: 27696108. doi:10.1007/s12031-016-0848-y	Study design did not include administration of antioxidants
Bahmani F, Kia M, Soleimani A, Mohammadi AA, Asemi Z. 2016. The effects of selenium supplementation on biomarkers of inflammation and oxidative stress in patients with diabetic nephropathy: a randomised, double-blind, placebo-controlled trial. PMID: 27647263. doi:10.1017/S0007114516003251	Study did not include predetermined outcomes, related to diabetic complications
Khatami PG, Soleimani A, Sharifi N, Aghadavod E, Asemi Z. 2016. The effects of high-dose vitamin E supplementation on biomarkers of kidney injury, inflammation, and oxidative stress in patients with diabetic nephropathy: A randomized, double-blind, placebo-controlled trial. PMID: 27578124. doi:10.1016/j.jacl.2016.02.021	Study conducted on non-target group of patients (non-diabetic, diabetes type 1 etc.)
Jin SM, Han KA, Yu JM, Sohn TS, Choi SH, Chung CH, Park IeB, Rhee EJ, Baik SH, Park TS, Lee IK, Ko SH, Hwang YC, Cha BS, Lee HW, Nam MS, Lee MK. 2016. Probucol in Albuminuric Type 2 Diabetes Mellitus Patients on Renin-Angiotensin System Blockade: A 16-Week, Randomized, Double-Blind, Placebo-Controlled Trial. PMID: 27493100. doi:10.1161/ATVBAHA.116.308034	Drug effect study
Voroneanu L, Nistor I, Dumea R, Apetrii M, Covic A. 2016. Silymarin in Type 2 Diabetes Mellitus: A Systematic Review and Meta-Analysis of Randomized Controlled Trials. PMID: 27340676. doi:10.1155/2016/5147468	Ineligible study type
Borges CM, Papadimitriou A, Duarte DA, Lopes de Faria JM, Lopes de Faria JB. 2016. The use of green tea polyphenols for treating residual albuminuria in diabetic nephropathy: A double-blind randomised clinical trial. PMID: 27320846. doi:10.1038/srep28282	Study conducted on non-target group of patients (non-diabetic, diabetes type 1 etc.)

Kato S, Ando M, Mizukoshi T, Nagata T, Katsuno T, Kosugi T, Tsuboi N, Maruyama S. 2016. Randomized control trial for the assessment of the anti-albuminuric effects of topiroxostat in hyperuricemic patients with diabetic nephropathy (the ETUDE study). PMID: 27303100. doi:	Drug effect study
Atkin M, Laight D, Cummings MH. 2016. The effects of garlic extract upon endothelial function, vascular inflammation, oxidative stress and insulin resistance in adults with type 2 diabetes at high cardiovascular risk. A pilot double blind randomized placebo controlled trial. PMID: 26954484. doi:10.1016/j.jdiacomp.2016.01.003	Study did not include predetermined outcomes, related to diabetic complications
Rafraf M, Bazyun B, Sarabchian MA, Safaeiyan A, Gargari BP. 2016. Vitamin E Improves Serum Paraoxonase-1 Activity and Some Metabolic Factors in Patients with Type 2 Diabetes: No Effects on Nitrite/Nitrate Levels. PMID: 26886716. doi:10.1080/07315724.2015.1116896	Study did not include predetermined outcomes, related to diabetic complications
Bahmani F, Kia M, Soleimani A, Asemi Z, Esmaillzadeh A. 2016. Effect of Selenium Supplementation on Glycemic Control and Lipid Profiles in Patients with Diabetic Nephropathy. PMID: 26686847. doi:10.1007/s12011-015-0600-4	Study did not include predetermined outcomes, related to diabetic complications
Ziegler D, Low PA, Freeman R, Tritschler H, Vinik AI. 2016. Predictors of improvement and progression of diabetic polyneuropathy following treatment with α-lipoic acid for 4 years in the NATHAN 1 trial. PMID: 26651260. doi:10.1016/j.jdiacomp.2015.10.018	Study conducted on non-target group of patients (non-diabetic, diabetes type 1 etc.)
Zhu H, Chen X, Cai G, Zheng Y, Liu M, Liu W, Yao H, Wang Y, Li W, Wu H, Lun L, Zhang J, Guan X, Yin S, Zhuang X, Li J, Liu Y, Zhou C. 2016. Telmisartan combined with probucol effectively reduces urinary protein in patients with type 2 diabetes: A randomized double-blind placebo-controlled multicenter clinical study. PMID: 26458146. doi:10.1111/1753-0407.12347	Drug effect study
Zemestani M, Rafraf M, Asghari-Jafarabadi M. 2016. Chamomile tea improves glycemic indices and antioxidants status in patients with type 2 diabetes mellitus. PMID: 26437613. doi:10.1016/j.nut.2015.07.011	Study did not include predetermined outcomes, related to diabetic complications
Singh RK, Gupta B, Tripathi K, Singh SK. 2016. Anti oxidant potential of Metformin and Pioglitazone in Type 2 Diabetes Mellitus: Beyond their anti glycemic effect. PMID: 26341927. doi:10.1016/j.dsx.2015.08.016	Drug effect study
Rodríguez-Carrizalez AD, Castellanos-González JA, Martínez-Romero EC, Miller-Arrevillaga G, Román-Pintos LM, Pacheco-Moisés FP, Miranda-Díaz AG. 2016. The antioxidant effect of ubiquinone and combined therapy on mitochondrial function in blood cells in non-proliferative diabetic retinopathy: A randomized, double-blind, phase IIa, placebo-controlled study. PMID: 26207797. doi:10.1179/1351000215Y.0000000032	Study did not include predetermined outcomes, related to diabetic complications
Chous AP, Richer SP, Gerson JD, Kowluru RA. 2016. The Diabetes Visual Function Supplement Study (DiVFuSS). PMID: 26089210. doi:10.1136/bjophthalmol-2014-306534	Study conducted on non-target group of patients (non-diabetic, diabetes type 1 etc.)
Tian ML, Shen Y, Sun ZL, Zha Y. 2015. Efficacy and safety of combining pentoxifylline with angiotensin-converting enzyme inhibitor or angiotensin II receptor blocker in diabetic nephropathy: a meta-analysis. PMID: 25862237. doi:10.1007/s11255-015-0968-2	Drug effect study

Ebrahimpour Koujan S, Gargari BP, Mobasseri M, Valizadeh H, Asghari-Jafarabadi M. 2015. Effects of Silybum marianum (L.) Gaertn. (silymarin) extract supplementation on antioxidant status and hs-CRP in patients with type 2 diabetes mellitus: a randomized, triple-blind, placebo-controlled clinical trial. PMID: 25765835. doi:10.1016/j.phymed.2014.12.010	Study conducted on non-target group of patients (non-diabetic, diabetes type 1 etc.)
Jiang DQ, Li MX, Wang Y, Wang Y. 2015. Effects of prostaglandin E1 plus methylcobalamin alone and in combination with lipoic acid on nerve conduction velocity in patients with diabetic peripheral neuropathy: A meta-analysis. PMID: 25800109. doi:10.1016/j.neulet.2015.03.037	Study conducted on non-target group of patients (non-diabetic, diabetes type 1 etc.)
Palma-Duran SA, Vlassopoulos A, Lean M, Govan L, Combet E. 2017. Nutritional intervention and impact of polyphenol on glycohemoglobin (HbA1c) in non-diabetic and type 2 diabetic subjects: Systematic review and meta-analysis. PMID: 25746842. doi:10.1080/10408398.2014.973932	Ineligible study type
Yubero-Serrano EM, Woodward M, Poretsky L, Vlassara H, Striker GE; AGE-less Study Group. 2015. Effects of sevelamer carbonate on advanced glycation end products and antioxidant/pro-oxidant status in patients with diabetic kidney disease. PMID: 25710801. doi:10.2215/CJN.07750814	Drug effect study
Kaatabi H, Bamosa AO, Badar A, Al-Elq A, Abou-Hozaifa B, Lebda F, Al-Khadra A, Al-Almaie S. 2015. Nigella sativa improves glycemic control and ameliorates oxidative stress in patients with type 2 diabetes mellitus: placebo controlled participant blinded clinical trial. PMID: 25706772. doi:10.1371/journal.pone.0113486	Study did not include predetermined outcomes, related to diabetic complications
Davies MJ, Trujillo A, Vijapurkar U, Damaraju CV, Meininger G. 2015. Effect of canagliflozin on serum uric acid in patients with type 2 diabetes mellitus. PMID: 25600248. doi:10.1111/dom.12439	Drug effect study
Aliasgharzadeh A, Dehghan P, Gargari BP, Asghari-Jafarabadi M. 2015. Resistant dextrin, as a prebiotic, improves insulin resistance and inflammation in women with type 2 diabetes: a randomised controlled clinical trial. PMID: 27028002. doi:10.1017/S0007114514003675	Study did not include predetermined outcomes, related to diabetic complications
Silva S, Bronze MR, Figueira ME, Siwy J, Mischak H, Combet E, Mullen W. 2015. Impact of a 6-wk olive oil supplementation in healthy adults on urinary proteomic biomarkers of coronary artery disease, chronic kidney disease, and diabetes (types 1 and 2): a randomized, parallel, controlled, double-blind study. PMID: 25527749. doi:10.3945/ajcn.114.094219	Study conducted on non-target group of patients (non-diabetic, diabetes type 1 etc.)
Navarro-González JF, Mora-Fernández C, Muros de Fuentes M, Chahin J, Méndez ML, Gallego E, Macía M, del Castillo N, Rivero A, Getino MA, García P, Jarque A, García J. 2015. Effect of pentoxifylline on renal function and urinary albumin excretion in patients with diabetic kidney disease: the PREDIAN trial. PMID: 24970885. doi:10.1681/ASN.2014010012	Drug effect study
Rajanandh MG, Kosey S, Prathiksha G. 2014. Assessment of antioxidant supplementation on the neuropathic pain score and quality of life in diabetic neuropathy patients - a randomized controlled study. PMID: 24905305. doi:10.1016/j.pharep.2013.08.003	Study did not include predetermined outcomes, related to diabetic complications
Martynyuk L, Martynyuk L, Ruzhitska O, Martynyuk O. 2014. Effect of the herbal combination Canephron N on diabetic nephropathy in patients with diabetes mellitus: results of a comparative cohort study. PMID: 24738695. doi:10.1089/acm.2013.0400	Study design did not include administration of antioxidants

Schneider MP, Schneider A, Jumar A, Kistner I, Ott C, Schmieder RE. 2014. Effects of folic acid on renal endothelial function in patients with diabetic nephropathy: results from a randomized trial. PMID: 24724807. doi:10.1042/CS20140111	Study did not include predetermined outcomes, related to diabetic complications
Raghavan RP, Laight DW, Cummings MH. 2014. Aspirin in type 2 diabetes, a randomised controlled study: effect of different doses on inflammation, oxidative stress, insulin resistance and endothelial function. PMID: 24372992. doi:10.1111/ijcp.12310	Drug effect study
Nikooyeh B, Neyestani TR, Tayebinejad N, Alavi-Majd H, Shariatzadeh N, Kalayi A, Zahedirad M, Heravifard S, Salekzamani S. 2014. Daily intake of vitamin D- or calcium-vitamin D-fortified Persian yogurt drink (doogh) attenuates diabetes-induced oxidative stress: evidence for antioxidative properties of vitamin D. PMID: 23829785. doi:10.1111/jhn.12142	Study design did not include administration of antioxidants
Gutierrez AD, Duran-Valdez E, Robinson I, de Serna DG, Schade DS. 2013. Does short-term vitamin C reduce cardiovascular risk in type 2 diabetes?. PMID: 23757614. doi:10.4158/EP12431.OR	Study did not include predetermined outcomes, related to diabetic complications
Mahjabeen W, Khan DA, Mirza SA. 2022. Role of resveratrol supplementation in regulation of glucose hemostasis, inflammation and oxidative stress in patients with diabetes mellitus type 2: A randomized, placebo-controlled trial. PMID: 36638933. doi:10.1016/j.ctim.2022.102819	Study conducted on non-target group of patients (non-diabetic, diabetes type 1 etc.)
Nyambuya TM, Nkambule BB, Mazibuko-Mbeje SE, Mxinwa V, Mokgalaboni K, Orlando P, Silvestri S, Louw J, Tiano L, Dludla PV. 2020. A Meta-Analysis of the Impact of Resveratrol Supplementation on Markers of Renal Function and Blood Pressure in Type 2 Diabetic Patients on Hypoglycemic Therapy. PMID: 33266114. doi:10.3390/molecules25235645	Study conducted on non-target group of patients (non-diabetic, diabetes type 1 etc.)
Xu Q, Pan J, Yu J, Liu X, Liu L, Zuo X, Wu P, Deng H, Zhang J, Ji A. 2013. Meta-analysis of methylcobalamin alone and in combination with lipoic acid in patients with diabetic peripheral neuropathy. PMID: 23664235. doi:10.1016/j.diabres.2013.03.033	Ineligible study type
Domanico D, Fragiotta S, Cutini A, Carnevale C, Zompatori L, Vingolo EM. 2015. Circulating levels of reactive oxygen species in patients with nonproliferative diabetic retinopathy and the influence of antioxidant supplementation: 6-month follow-up. PMID: 25686055. doi:10.4103/0301-4738.151455	Ineligible study type
